# Catalytic Oxidation of Carbon–Halogen Bonds by Water with H_2_ Liberation

**DOI:** 10.1021/jacs.5c11295

**Published:** 2025-08-09

**Authors:** Cai You, Lijun Lu, David Milstein

**Affiliations:** † Department of Molecular Chemistry and Materials Science, 34976 Weizmann Institute of Science, Rehovot 76100, Israel; ‡ Departmentof Chemistry, School of Sciences, Great Bay University, Dongguan 523000, People’sRepublic of China

## Abstract

The unprecedented catalytic oxidation of carbon–halogen bonds to carboxylic acids using water as the oxidant is disclosed. Compared to previous traditional oxidation reactions, this transformation avoids the use of sacrificial oxidants and liberates useful hydrogen gas as byproduct, presenting an efficient method. Catalyzed by an acridine-based PNP-Ru pincer complex, a series of primary aliphatic and benzylic halides were successfully converted into carboxylic acids in high yields. The oxidation of secondary halides, which yields ketones, was also accomplished efficiently. Moreover, the oxidation of challenging C–F bonds, aliphatic chlorides, and bromides has been achieved for the first time. With further improvement, this method could be effectively utilized in the efficient scale-up synthesis of the phenoxybutyric herbicide, MCPB. Furthermore, a formal anti-Markovnikov oxidation of nonactivated olefins to carboxylic acids has also been demonstrated through a two-step sequence involving anti-Markovnikov hydrobromination followed by oxidation of the resulting alkyl halides.

## Graphical abstract

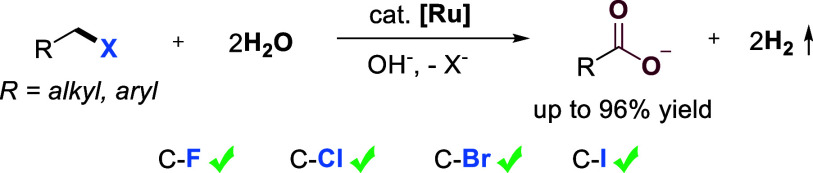

## Introduction

Halides are extremely versatile building blocks in organic synthesis, enabling the construction of complex molecules and facilitating the development of new materials, pharmaceuticals, and agrochemicals.^1,2^ However, their extensive use has led to the release of halogenated organic pollutants (HOPs) into the environment. Therefore, the development of efficient transformation or degradation of halogenated organic compounds are in demand.^3–8^ Among the various transformations of halides, their oxidation to carbonyl compounds, which are very important both in academia and industry, has received considerable attention by synthetic organic chemists.^9,10^ For example, the oxidation of aromatic and aliphatic halides to aldehydes or ketones has long been achieved with various methods, such as Kornblum oxidation, Sommelet oxidation, Ganem oxidation, Hass–Bender Oxidation and so on.^9,10^ However, direct, selective oxidation of carbon–halogen bonds to carboxylic acids, which has long been considered to be an important but challenging transformation, has been rarely reported. To date, most of the methods developed rely on the use of stoichiometric amounts of strong and toxic oxidants, such as H_2_O_2_, *tert*-butyl hydroperoxide (TBHP), oxone etc.^11–16^ Under these conditions, oxidation-sensitive functional groups are usually not tolerated. Due to the limitations in these reported transformation mechanisms, only the oxidation of aromatic halides into aromatic acids have been realized, and the oxidation of aliphatic halides into aliphatic acids is exceedingly rare. To the best of our knowledge, there is only a single example involving oxidation of 1-iodobutane into butyric acid, in only 7% yield.^11^ Besides, the oxidation of aliphatic chlorides and aliphatic bromides to carboxylic acids remains unexplored. Moreover, because of the high bond energy, the oxidation of carbon–fluorine bonds into either carboxylic acids or other carbonyl compounds has not yet been achieved. Thus, there is a strong demand for the catalytic oxidation of halides to carboxylic acids without the use of oxidants, accommodating a wide range of substrates, including both aromatic and aliphatic halides, while also demonstrating tolerance toward oxidation-sensitive functional groups.

The utilization of water as an oxidant in catalytic processes for organic synthesis, accompanied by the liberation of H_2_, represents a challenging yet ideal method, which stands out as one of the most environmentally friendly means for selective oxidation of organic compounds.^17–29^ In this research area, our group has achieved a series of catalytic oxidation reactions by water with hydrogen gas liberation, facilitated by ruthenium pincer complexes.^30^ In 2013, we reported the catalytic oxidation of primary alcohols to carboxylic acid salts using alkaline water along with liberation of hydrogen gas, using a bipyridine-based PNN-Ru complex **Ru-2** as the catalyst.^17^ In 2020, our acridine-based PNP-Ru complex **Ru-3** enabled the oxidation of primary amines to carboxylates using only water as the oxidant with H_2_ liberation.^21^ Very recently, by using **Ru-3** as the catalyst, we achieved the oxidation of the biomass-derived renewable aldehydes furfural and 5-hydroxymethylfurfural to furoic acid and furandicarboxylic acid, respectively, with alkaline water as the oxidant, liberating H_2_.^23^

Although the oxidation of alcohols, aldehydes and amines using water as the oxidant has been achieved by us and others, to the best of our knowledge, the catalytic oxidation of carbon–halogen bonds to carboxylic acids with water as the oxidant remains unreported. The catalytic oxidation of carbon–halogen bonds faces several challenges: (1) direct hydrolysis of halides into alcohols is not easy, especially for aliphatic halides;^31–35^ (2) under basic conditions, several side reactions may potentially occur, such as ether formation via the reaction between halides and alcohols produced during hydrolysis,^31–33,36^ elimination reactions of halides,^34,35^ and α-alkylation of carbonyl products;^29^ (3) catalysts must tolerate C–X bonds. Moreover, the requirement of a water-resistant nature, which is necessary for the utilization of water for hydrolysis and as an oxidant, makes this catalytic transformation further challenging. Herein, we present the unprecedented example of an efficient method for catalytic oxidation of carbon–halogen bonds, utilizing water as the oxidant with H_2_ liberation (Scheme 1c). Using an acridine-based PNP-Ru complex as the catalyst, this method is applicable to the oxidation of both benzylic and aliphatic halides into carboxylic acids with high selectivity. Furthermore, oxidation of the challenging C–F bonds has been successfully achieved for the first time. Notably, this strategy eliminates the need for any sacrificial oxidant. In addition, this catalytic oxidation protocol has been successfully extended to a formal anti-Markovnikov oxidation of nonactivated olefins to carboxylic acids via a two-step sequence involving hydrobromination followed by halide oxidation.

**Scheme 1:**
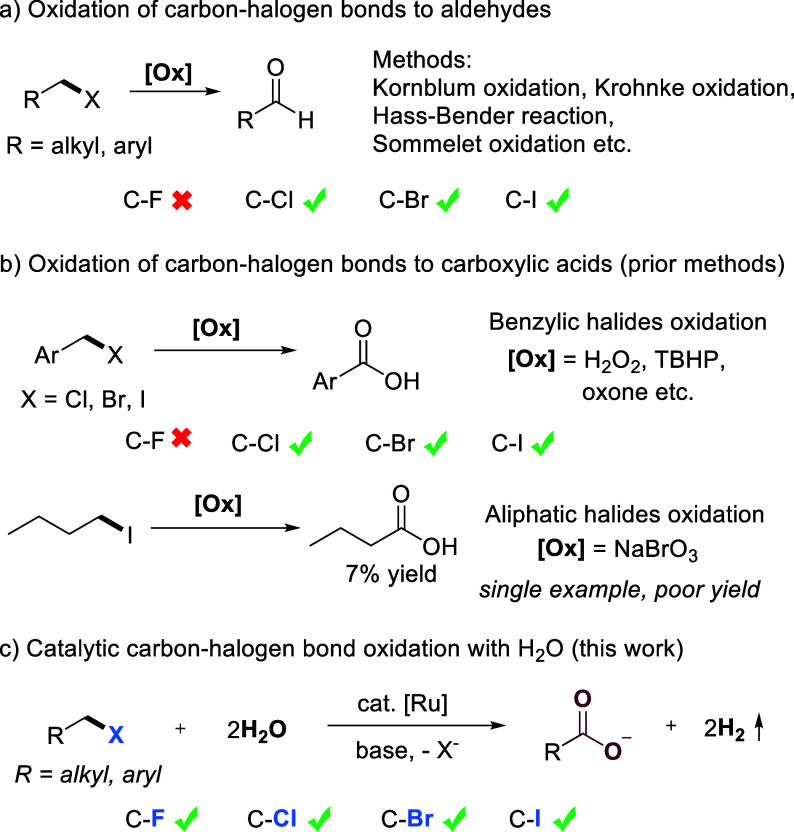
Oxidation of Carbon–Halogen Bonds

## Results and Discussion

### Condition Optimization

We selected 1-chlorooctane (**1a**), an aliphatic alkyl chloride representing a type of substrate that remains unexplored, as a model substrate to investigate the targeted oxidation of carbon–halogen bonds (Figure 1). First, a series of Ru-pincer complexes developed in our group were screened using NaOH as the base, with water and 1,4- dioxane as the cosolvent (Figure 1a).

**Figure 1:**
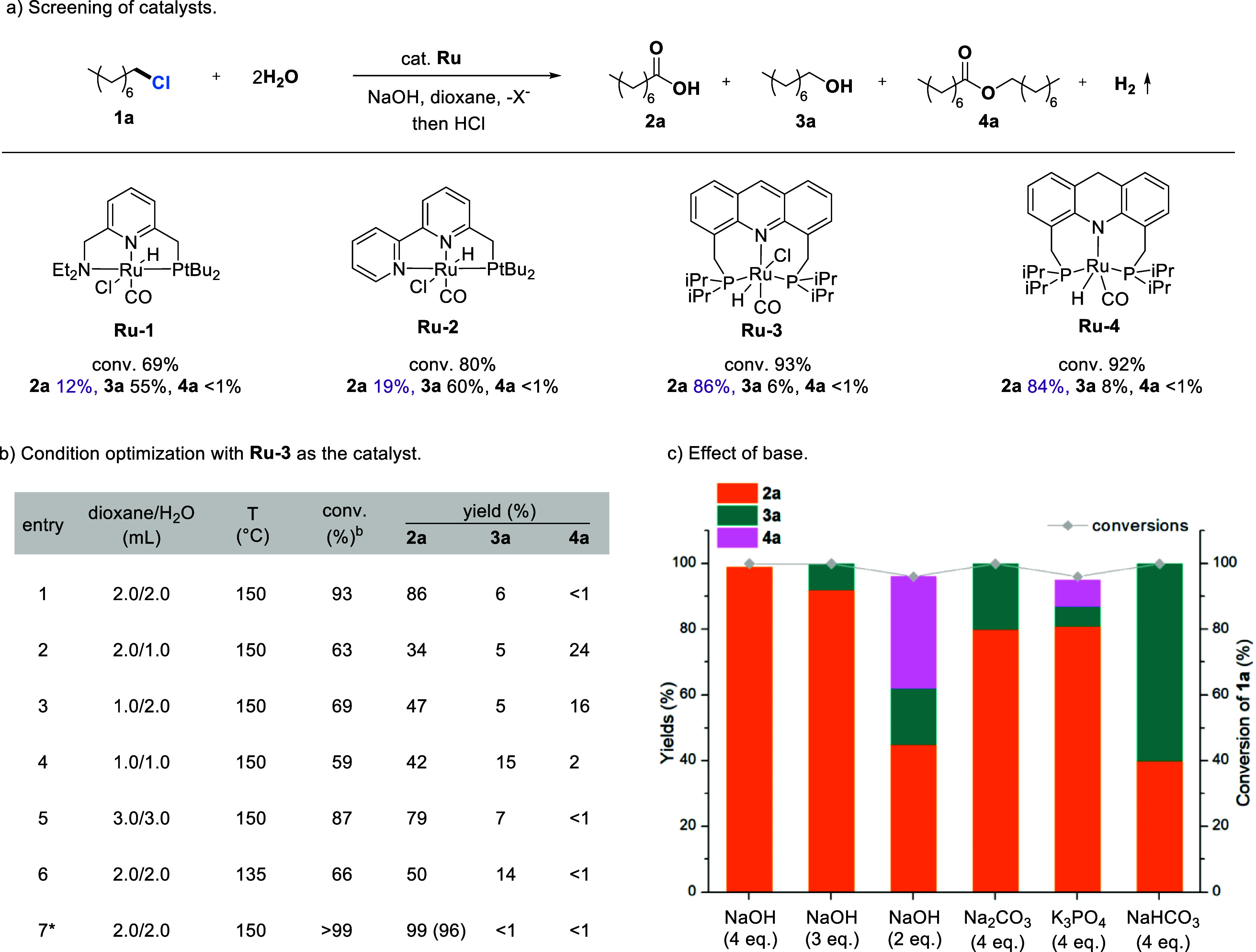
Catalytic oxidation of primary halides to carboxylates using H_2_O with H_2_ liberation. (a) Screening of catalysts. (b) Condition optimization. (c) Effect of base. Reactions in (a) were conducted using 0.5 mmol of **1a**, 1.5 mol % catalyst, and NaOH (2.0 mmol) in 1,4-dioxane (2 mL)/water (2 mL), heated in a sealed tube at 150 °C (silicon oil bath temperature) for 20 h. Reactions in (b) were conducted using 0.5 mmol of **1a**, 1.5 mol % **Ru-3**, and NaOH (2.0 mmol) in 1,4-dioxane/water, heated in a sealed tube at 150 or 135 °C (silicon oil bath temperature) for 20 h. *48 h. Reactions in (c) were conducted using 0.5 mmol of **1a**, 1.5 mol % **Ru-3**, and base in 1,4-dioxane (2 mL)/water (2 mL), heated in a sealed tube at 150 °C (silicon oil bath temperature) for 48 h. Conversions and yields were determined by ^1^H NMR (dibromomethane as an internal standard, isolated yields in parentheses).

As shown in Figure 1a, heating the resulting solution at 150 °C for 20 h with **Ru-1** as the catalyst yielded only 12% of the oxidation product **2a** and 55% of the hydrolysis product **3a**. Using **Ru-2** as the catalyst, the yield of **2a** increased to 19%, with 60% yield of **3a**. Remarkably, our acridine PNP complex **Ru-3** catalyzed the reaction with high oxidation product yield (86%) and low **3a** yield (6%). The dearomatized acridine PNP complex **Ru-4** displayed similar catalytic activities to **Ru-3,** producing 84% yield of **2a** with 8% of **3a**. To further improve the yield of the oxidation product **2a**, we undertook a systematic optimization of the reaction conditions using **Ru-3** as the catalyst (Figure 1b). Given that the quantity of solvent and the water-to-dioxane ratio may affect both the hydrolysis and oxidation steps, we initially tested the impact of solvent composition. Reducing the amounts of either water or dioxane dramatically led to lower conversions of **1a**, significantly reduced yields of **2a**, and increased formation of ester **4a** (Figure 1b, entries 1–4). Interestingly, increasing the amounts of both water and dioxane resulted in slightly lower yields of **2a** and **3a** (entry 5 vs entry 1). Conducting the reaction at 135 °C for 20 h resulted in only 66% conversion, with 50% yield of **2a** and 14% yield of **3a** (entry 6). Extending the reaction time to 48 h, resulted in full conversion, obtaining a 99% yield of **2a** and a 98% yield of H_2_ (entry 7).

### Effect of Base Amount and Strength

Next, the effect of various NaOH amounts was tested. Using 3 eq. NaOH resulted in full conversion of **1a**, yielding 92% of **2a**, with 8% yield of **3a** left in the system. Further reducing the amount of NaOH to 2 equiv resulted in a 96% conversion of **1a**, but only a 45% yield of **2a**, with significant amounts of **4a** (34%) and **3a** (17%). Next, the effect of different bases was also investigated. Relatively weaker bases, such as Na_2_CO_3_ and K_3_PO_4_, led to decreased yields of **2a** (80% and 81%, respectively). Further reducing the base strength, as with NaHCO_3_, resulted in an even lower yield of **2a** (40%), with the remaining product being the alcohol **3a** (60%). These results indicate that both the amount and strength of the base are critical for the catalytic oxidation of **1a** to **2a**. Using 2.4 equiv NaOH, full conversion of **1a** was achieved after 144 h, but **2a** was obtained in only 59% yield, with **4a** (32%) and **3a** (9%) formed as byproducts. With 3 equiv NaOH and extending the reaction time to 96 h, 98% yield **2a** was obtained (Table S1). Finally, we chose to use 4 equiv NaOH to investigate the substrate scope.

### Catalytic Oxidation of Primary Alkyl Halides to Carboxylates

With the optimal reaction conditions in hand, we explored the substrate scope for this catalytic oxidation of carbon–halogen bonds by water, as shown in Table 1. Besides the C–Cl bond (**1a**), C–Br (**1b**) and C–I (**1c**) bonds also exhibited high reactivity, giving the corresponding carboxylic acids in high yields. Our catalytic system also demonstrated high selectivity and efficiency in the oxidation of 1-chloro-4-methoxybutane (**1d**), (3-chloropropyl)­benzene (**1e**) and (4-chlorobutoxy)­benzene (**1f**) (Table 1, entries 4–6). When (4-chlorobutyl)­(phenyl)­sulfane (**1g**) was employed as the substrate, a significantly lower yield was observed, likely attributable to the decomposition of **1g** during the reaction (see Supporting Information, page S8). To further explore the functional group and heterocycle tolerance of the reaction, a range of structurally diverse substrates were evaluated. The methodology exhibits broad substrate scope with respect to heteroaromatic motifs. A variety of common five-membered heteroaromatic compounds, including indole (**1h**), furan (**1i**), thiophene (**1j**), pyrrole (**1k**), and carbazole (**1l**), are well tolerated, delivering the corresponding carboxylic acids in good to excellent yields (Table 1, entries 8–12). In addition, the protocol accommodates other functional groups such as trimethylsilyl (**1m**), diphenylamino (**1n**), and sulfone (**1o**) substituents without compromising the reaction efficiency (Table 1, entries 13–15). Notably, more structurally elaborate or privileged heterocyclic scaffolds such as phenoxazine (**1p**) and phenothiazine (**1q**) also undergo smooth transformation under the standard conditions, highlighting the robustness and synthetic utility of the method (Table 1, entries 16 and 17).

**Table 1:**
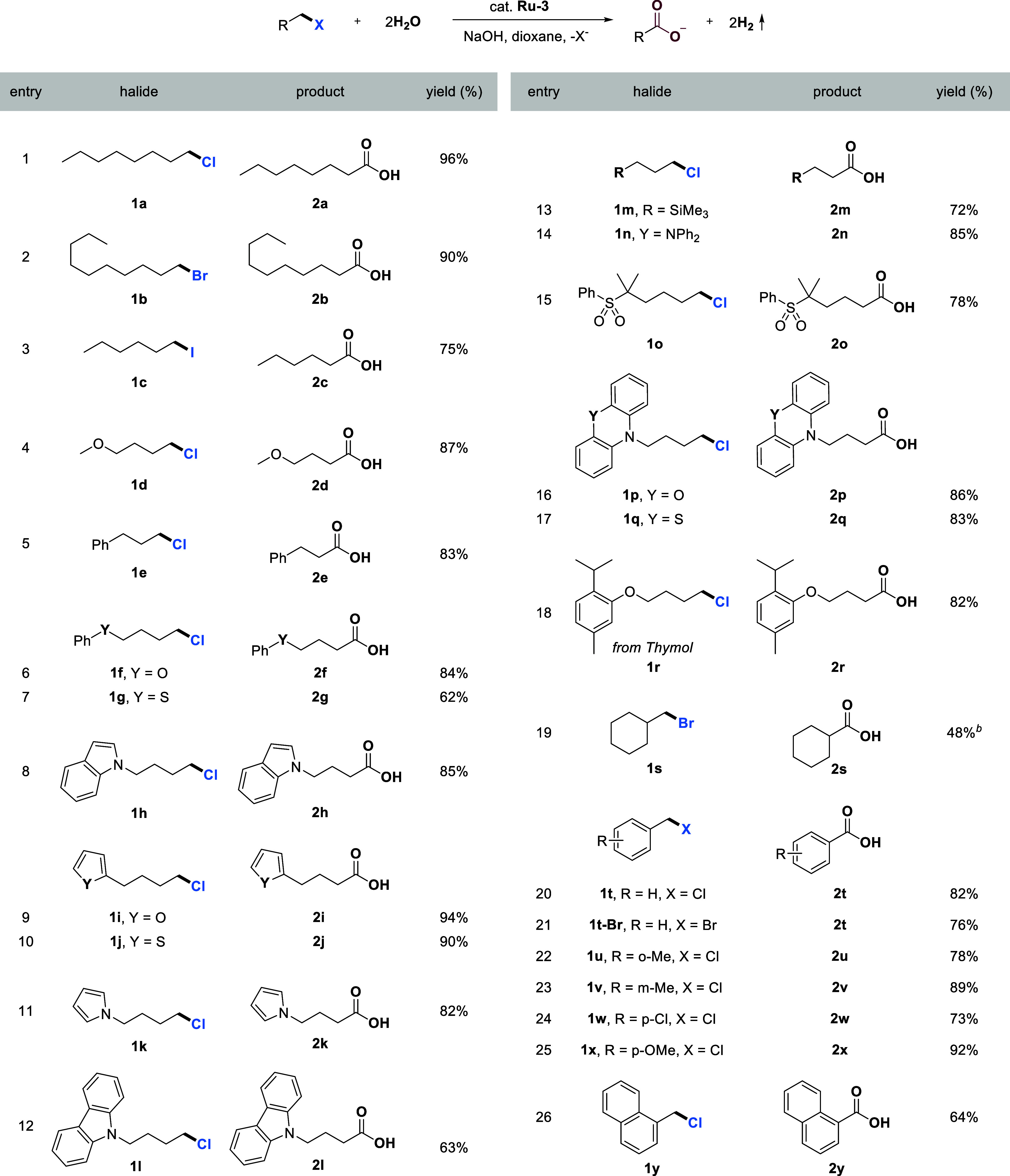
Catalytic Oxidation of Primary Alkyl Halides to Carboxylates Using Water with H_2_ Liberation^a^ ^b^

a General reaction conditions: Halides (0.50 mmol), **Ru-3** (0.0075 mmol), NaOH (2.0 mmol), water (2.0 mL), and dioxane (2.0 mL) were heated in a closed system at 150 °C (silicon oil bath temperature, solvent reflux) for 48 h. Yields of isolated products are displayed.

b K_2_CO_3_ (1.0 mmol) was used instead of NaOH.

Furthermore, a substrate derived from the natural product thymol (**1r**) is also well tolerated. Employing the bulky primary halide **1s**, a lower yield (48%) was obtained with K_2_CO_3_ as the base, primarily due to the occurrence of an elimination side reaction, which was observed by ^1^H NMR of the crude reaction mixture. Subsequently, our catalytic system was also investigated in the oxidation of benzylic halides, exhibiting high reactivity and selectivity as well. (Chloromethyl)­benzene (**1t**) and (bromomethyl)­benzene (**1t-Br**) were transformed to benzoic acid in good yields (Table 1, entries 20 and 21). Benzylic halides bearing substituents at various positions and different electronic properties were effectively accommodated, affording the corresponding carboxylic acids in yields ranging from 73% to 92% (Table 1, entries 22–25). Using 1-(chloromethyl)­naphthalene (**1y**), resulted in a moderate yield of 64% due to increased steric hindrance (Table 1, entry 26).

### Catalytic Oxidation of Secondary Alkyl Halides to Ketones

Next, we extended this methodology to the oxidation of secondary halides, resulting in the formation of ketones. Although many methods have been reported for this transformation, using water as the oxidant is very rare.^9,10^ As shown in Table 2, good selectivity and efficiency were achieved by heating an alkaline water/dioxane (1:4 volumetric ratio) solution of secondary halides in the presence of complex **Ru-3**. High yields of 85% and 84% were obtained for the oxidation of (1-chloroethyl) benzene **1z** and (1-bromoethyl) benzene **1z-Br**, respectively. The oxidation of benzylic chloride **1aa**, which has a long alkyl chain, produced the ketone product with a 62% yield, while the main byproduct was (*E*)-pent-1-en-1-ylbenzene, resulting from elimination. A similar outcome was observed in the oxidation of secondary aliphatic chloride **1ab**, where **2ab** was obtained in moderate yield. Moreover, the oxidation of cycloalkyl halide **1ac** afforded the corresponding cyclic ketone **2ac** in good yield. For the oxidation of diaryl substituted benzylic secondary halide **1ad**, reaction proceeded very smoothly, affording the corresponding diary ketone **2ad** in almost quantitative yield.

**Table 2:**
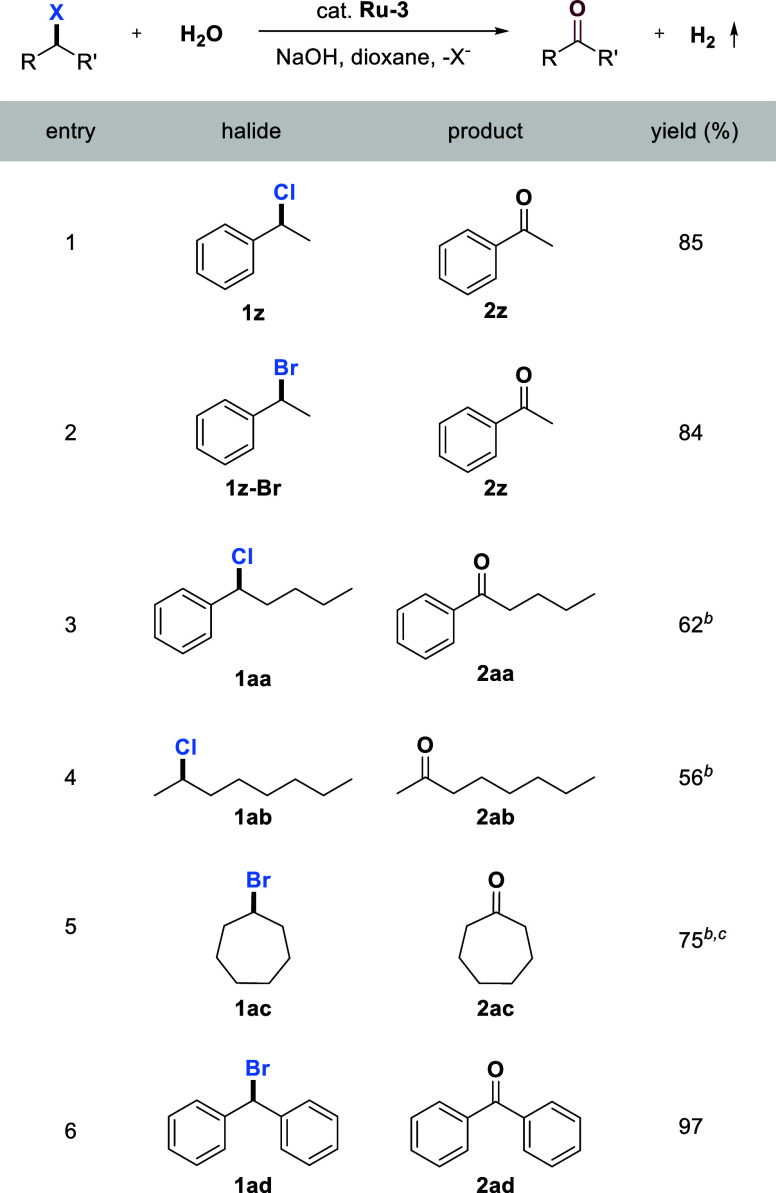
Catalytic Oxidation of Secondary Halides to Ketones Using Water with H_2_ Liberation^a^ ^b^ ^c^

a General reaction conditions: Halides (0.50 mmol), **Ru-3** (0.0075 mmol), NaOH (0.60 mmol), water (0.50 mL), and dioxane (2.0 mL) were heated in a closed system at 150 °C (silicon oil bath temperature, solvent reflux) for 48 h. Isolated yields are displayed.

b NaOH (1.0 mmol) was used.

c Yields were determined by GC using mesitylene as an internal standard.

### Catalytic Oxidation of C–F Bonds

In comparison to other C–X bonds (X = Cl, Br, I), transformation of the much stronger C–F bond^37^ is significantly more challenging. Due to its low reactivity, the C–F bond remains inert under most reaction conditions, allowing it to be safely carried through multistep syntheses with minimal concern for side reactions. Among the halogens in alkyl halides, fluoride exhibits the poorest leaving group ability, with the ranking as follows: I > Br > Cl ≫ F. In recent years, there has been growing interest in C–F bond transformations, aiming to utilize fluoride as a leaving group in substitution reactions, which traditionally necessitate more activated leaving groups.^38–41^ To date, the oxidation of C–F bonds to carbonyl compounds, including aldehydes, ketones, or carboxylic acids, remains an undeveloped area of research. Here we disclose the oxidation of C–F bonds for the first time.

As shown in Table 3, primary benzyl fluorides are converted into the corresponding carboxylic acids (**2ae** and **2af**) in high yields using alkaline water as the oxidant, without the need for any additional oxidant, liberating H_2_ as the byproduct. To our delight, the oxidation of a more challenging aliphatic fluoride, **1e-F**, was also successfully achieved by employing 6 equiv of LiOH and 3 mol % of **Ru-3**, albeit with a moderate yield of 48%. The relatively low yield was primarily due to the inherent difficulty of the hydrolysis step, as reflected by the limited conversion of **1e-F** (52%). Next, the oxidation of secondary benzyl fluorides was also investigated. Due to a significant elimination side reaction, the oxidation of **1ag** was not very efficient, resulting in the formation of the corresponding ketone **2ag** with only a 40% yield. In contrast, the oxidation of the diaryl substituted benzyl fluoride **1ad-F** proceeded very smoothly and gave ketone **2ad** in high yield of 82% (Table 3, entry 5).

**Table 3:**
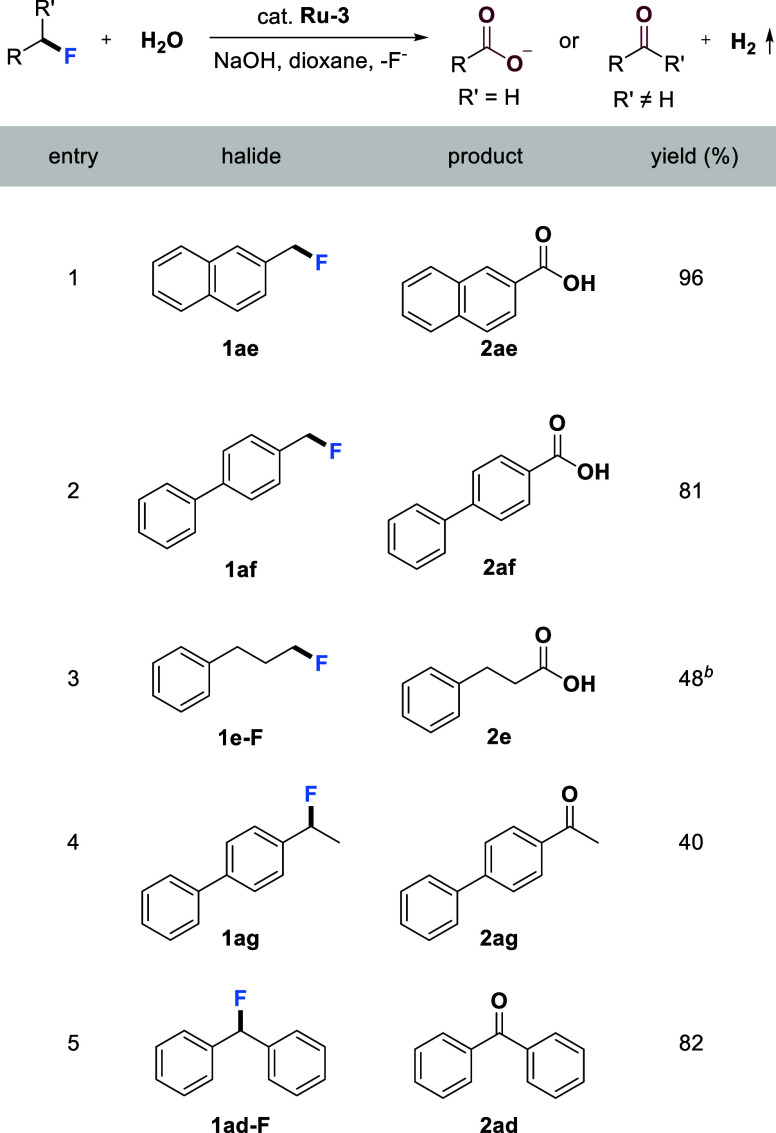
Catalytic Oxidation of C–F Bonds Using Water with H_2_ Liberation^a^ ^b^

a For **1ae**, **1af** and **1e-F**: Fluorides (0.25 mmol), **Ru-3** (0.0038 mmol), NaOH (1.0 mmol), water (1.0 mL), and dioxane (1.0 mL) were heated in a closed system at 150 °C (silicon oil bath temperature, solvent reflux) for 72 h. For **1ag** and **1ad-F**: Fluorides (0.25 mmol), **Ru-3** (0.0038 mmol), NaOH (0.50 mmol), water (0.25 mL), and dioxane (1.0 mL) were heated in a closed system at 150 °C (silicon oil bath temperature, solvent reflux) for 72 h. Isolated yields are displayed.

b **1e-F** (0.50 mmol), **Ru-3** (0.015 mmol), LiOH (3.0 mmol), water (2.0 mL), and dioxane (2.0 mL) were heated in a closed system at 150 °C (silicon oil bath temperature, solvent reflux) for 72 h. Yields were determined by ^1^H NMR (dibromomethane as an internal standard).

### Mechanistic Investigation

According to previous studies by us and Hofmann et al.,^42–44^ **Ru-3** can readily undergo reduction by alcohols or amines under basic conditions to form the corresponding dearomatized complex, **Ru-4**. As expected, **Ru-4** gave quite similar results to those of **Ru-3** in the oxidation of **1a,** affording **2a** in almost quantitative amounts in the presence of NaOH. To understand the roles of NaOH and **Ru-4** in this transformation, a series of control experiments were carried out (Control experiments for the benzyl fluoride substrate **1ae**, which showed similar results to those for **1a**, are provided in the Supporting Information). As shown in Scheme 2a, in the absence of either NaOH or **Ru-4**, or both, no formation of **2a** was observed, indicating the crucial roles of NaOH and **Ru-4** in the oxidation of **1a**. Surprisingly, the addition of 4 equiv of NaOH alone did not result in full conversion of **1a**, unlike the full conversion observed when both NaOH and **Ru-4** are present. We speculated that the carboxylates formed during the reaction might assist the hydrolysis step.^31,33^ To confirm this, a reaction was conducted with 0.4 eq. of **2a** and 4 eq. of NaOH, and a full conversion of **1a** was achieved, affording **3a** in a 99% yield. Next, control experiments were also carried out in the oxidation of the secondary halide **1z** (Scheme 2b). Again, **Ru-4** gave quite similar results to those of **Ru-3,** converting **1z** to the corresponding ketone **2z** in the presence of NaOH. In the absence of either NaOH, **Ru-4**, or both, no formation of **2z** was observed, highlighting the crucial roles of NaOH and **Ru-4** in the oxidation of the secondary halide **1z**. Notably, although high conversion of **1z** was achieved in the absence of NaOH, the very low selectivity for **3z** underscores the importance of NaOH in the selective hydrolysis step. Since alcohols are believed to be generated from the hydrolysis step in aqueous solutions, the oxidation of primary alcohol **3a** by water was also investigated. When **Ru-4** was employed in the oxidation of primary alcohol **3a** in the absence of NaOH, only 12% yield of **2a** was formed, as expected according to our previous reports.^21^ However, upon addition of 1.2 equiv of NaOH to the system, almost full conversion of the primary alcohol **3a** to carboxylic acid **2a** was observed (Scheme 2c). In contrast, the dehydrogenation of the secondary alcohol **3z** by complex **Ru-4** proceeded smoothly in the absence of NaOH, affording an excellent yield (94%) of the ketone product **2z** (Scheme 2d). Based on these experimental results, we hypothesized that the HCl formed during the hydrolysis step could shut down the reaction when the oxidation of C-X bonds was carried out in the absence of a base. To validate this hypothesis, the dehydrogenation of **3a** and **3z** was performed using **Ru-4** as the catalyst in the presence of 10 mol % HCl. The reaction yielded almost no desired oxidation products (Scheme 2e,f), indicating that HCl acts as a poison in this transformation. Based on the experimental results and our previous reports,^21^ a plausible mechanism for the catalytic oxidation of halides by alkaline water with H_2_ liberation is proposed (Scheme 2g). NaOH can promote the hydrolysis of halides to afford the corresponding alcohols, and it is very important for the high selectivity in the hydrolysis of the secondary halide **1z** to **3z**. Simultaneously, NaOH neutralizes the HCl formed during this hydrolysis step, preventing the deactivation of **Ru-4**. After hydrolysis of the halide substrates to the corresponding alcohols, **Ru-4** can catalyze the oxidation of primary halide **1a** (via alcohol **3a**) to carboxylic acid **2a** in the presence of NaOH with the liberation of 2 equiv of H_2_, and the oxidation of secondary halide **1z** (via alcohol **3z**) to ketone **2z** even in the absence of base, with the release of 1 equiv of H_2_. These mechanistic studies reveal that the presence of a base is essential not only for facilitating the hydrolysis of halides to alcohols but also for governing reaction selectivities and preserving catalyst activity. For the catalytic oxidation of the primary halide **1a**, in theory, two equivalents of NaOH are sufficient; however, an excess of base significantly facilitates the complete conversion of **1a** to **2a** more efficiently.

**Scheme 2:**
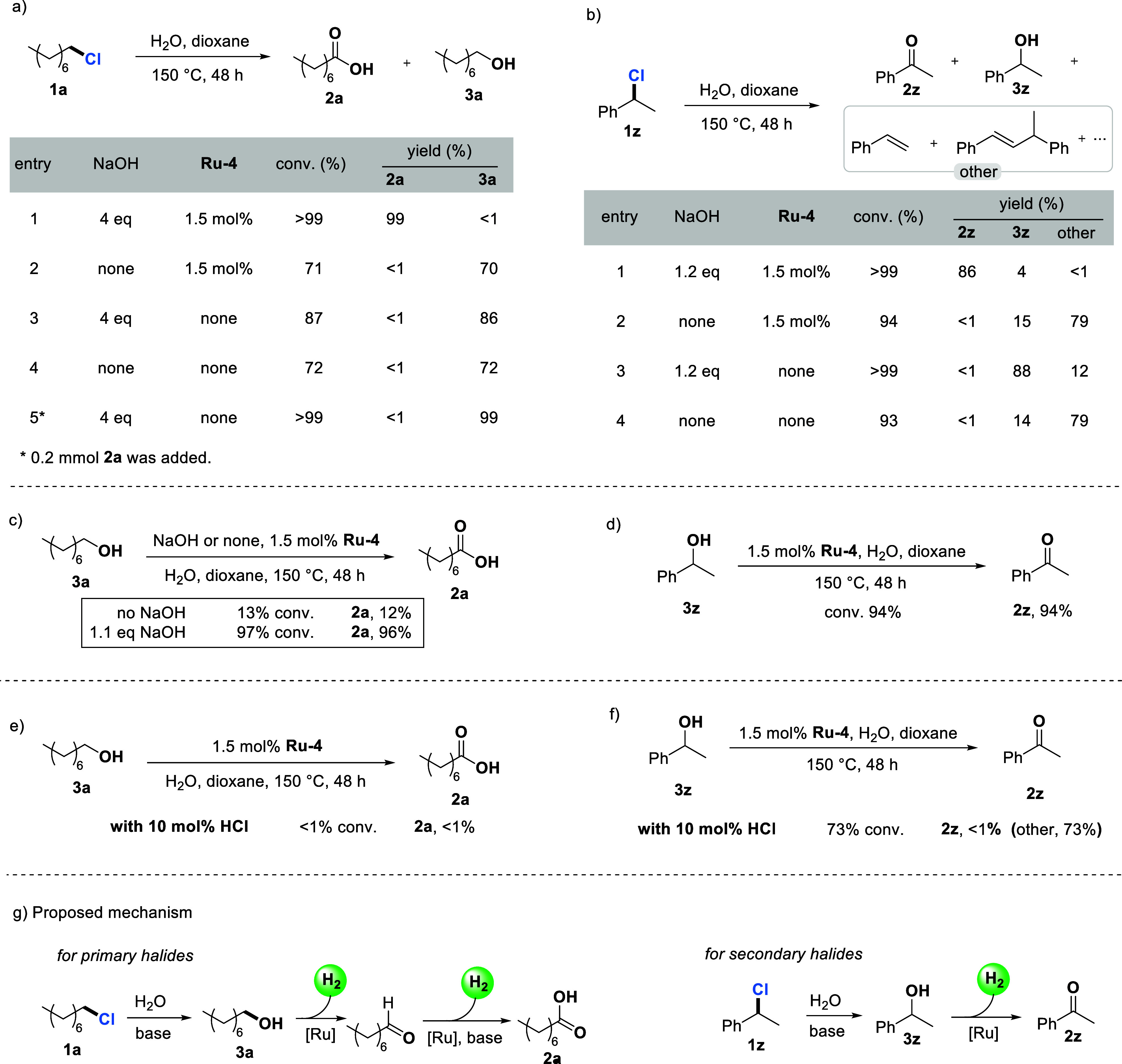
Mechanistic Experiments

### Scale-Up and Application

To demonstrate the synthetic utility of the current methodology, a creative synthetic route for MCPB, a phenoxybutyric herbicide,^45^ was developed (Scheme 3). Starting with commercially available chemicals, 4-chloro-2-methylphenol and 1-bromo-4-chlorobutane, 4-chloro-1-(4-chlorobutoxy)-2-methylbenzene (**1ah**) was synthesized in high yield (96%). Subsequently, upon decreasing the catalyst loading to 0.2 mol %, a gram-scale experiment using 5 mmol of **1ah** (1.17 g) was conducted to assess the scalability and applicability of the current oxidation methodology. After 72 h of reaction at 150 °C, 0.99 g of MCPB (**2ah**) (87% yield) was isolated, along with 231 mL of H_2_ collected (94% yield),^46^ thereby demonstrating the efficiency of this transformation for large-scale synthesis.

**Scheme 3:**
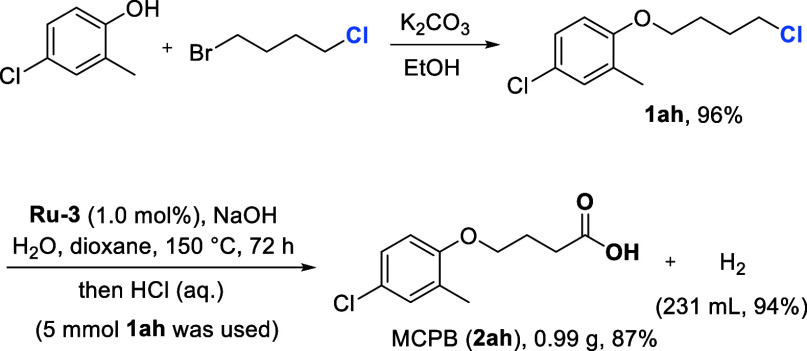
Synthesis of MCPB

### Formal Anti-Markovnikov Oxidation of Nonactivated Olefins to Carboxylic Acids

To further demonstrate the synthetic potential and conceptual flexibility of our strategy, we applied the protocol to a two-step transformation of nonactivated alkenes into carboxylic acids (Table 4). Specifically, the olefins (**4a**, **4b**) first underwent anti-Markovnikov hydrobromination,^47^ followed by oxidation of the resulting alkyl bromides to furnish the corresponding carboxylic acids in high yields.^46^ This overall sequence constitutes a formal anti-Markovnikov oxidation of nonactivated alkenes, a transformation that remains rare in the literature.^48,49^ These results highlight the broader utility and strategic value of our method in oxidative alkene functionalization.

**Table 4:**
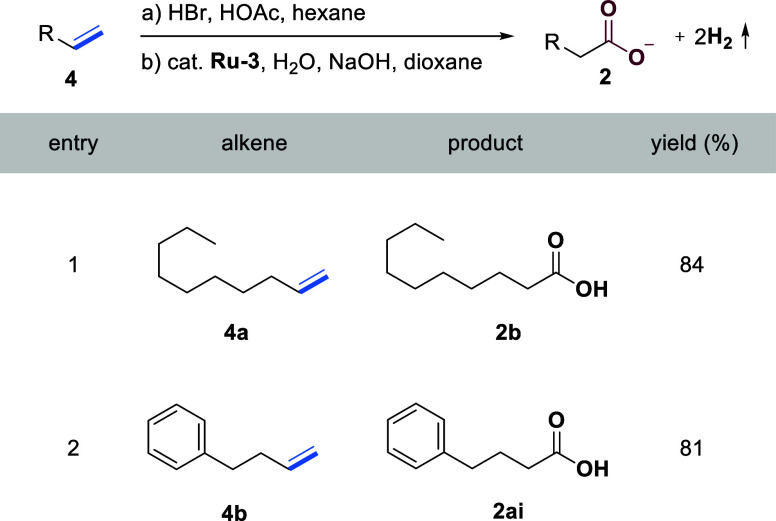
Formal Anti-Markovnikov Oxidation of Nonactivated Olefins to Carboxylic Acids^a^

a General reaction conditions: (a) Alkenes (0.5 mmol), HBr (1.0 mmol, 33% v/v in AcOH), hexane, 0 °C, 2 h. (b) **Ru-3** (0.0075 mmol), NaOH (2.0 mmol), water (2.0 mL), and dioxane (2.0 mL) were heated in a closed system at 150 °C (silicon oil bath temperature, solvent reflux) for 48 h. Yields of isolated products are displayed.

## Conclusions

We have developed the first catalytic oxidation of carbon–halogen bonds using water as the oxidant with concomitant hydrogen liberation. In contrast to previous traditional oxidation methods, this reaction does not require additional oxidants. A wide variety of primary halides were selectively and efficiently transformed into carboxylates, while secondary halides were converted to ketones. Notably, this method also successfully oxidizes the challenging C–F bonds for the first time. Our mechanistic studies indicate that the presence of a base is crucial not only for facilitating the hydrolysis of halides to alcohols, but also for influencing the reaction selectivities and maintaining catalyst activity. Moreover, this homogeneous process was successfully applied in the large-scale oxidation of the challenging primary aliphatic chloride **1ah** to MCPB, demonstrating the practical potential of this green methodology. Building on this catalytic halide oxidation, we have also demonstrated a formal anti-Markovnikov oxidation of nonactivated olefins to carboxylic acids through a two-step sequence involving anti-Markovnikov hydrobromination followed by oxidation of the resulting alkyl halides. This transformation further highlights the synthetic potential and conceptual flexibility of our water-based oxidation strategy. More oxidation reactions using water as the oxidant are undergoing in our lab.

## Supplementary Material



## Notes

Experimental details, characterization data, NMR spectra (PDF)
