# Supplementary material for: Catalytic Oxidation of Carbon–Halogen Bonds by Water with H2 Liberation
Source: J Am Chem Soc. 2025 Aug 9;147(33):30490–8. doi: 10.1021/jacs.5c11295 (PMC12371878; doi:10.1021/jacs.5c11295)
Supplement: Supplementary file 1 [file ja5c11295_si_001.pdf]

Supporting Information

Catalytic Oxidation of Carbon-Halogen Bonds by Water with H_2 Liberation

Cai You, Lijun Lu, David Milstein*

Department of Molecular Chemistry and Materials Science, The Weizmann Institute of Science, Rehovot 7610001, Israel.
E-mail: david.milstein@weizmann.ac.il.

Table of Contents

1. General information..2

2. Effect of base amount and strength…………………………………………...………………3

3. Catalytic oxidation of carbon-halogen bonds...4

4. Formal anti-Markovnikov oxidation of nonactivated olefins to carboxylic acids……..............28

5. Control experiments with benzyl fluoride substrate **1ae**……..30

6. NMR spectra………...31

7. References…………………………………………………………………….…..............66

1. General information

All reactions were performed under an atmosphere of purified nitrogen in an MBraun glovebox, or by using standard Schlenk techniques, unless otherwise noted. All commercially available reagents were used as received. 1,4-dioxane was purified prior to use by refluxing and distilling over Na/benzophenone under an argon atmosphere. Water was purified on a Synergy UV Water Purification System, and was degassed prior to use by bubbling argon for at least 20 min. **Ru-1**,[1] **Ru-2**,[2] **Ru-3**,[3] **Ru-4**,[4] **1g**,[5] **1h**,[6] **1aa**,[7] **1ae**,[8] **1af**,[8] **1e-F**,[9] **1ag**[10] and **1ad-F**[10] were synthesized according to reported procedures. Hydrogen gas was detected by GC analysis on an HP 6890 chromatograph (TCD detector) with nitrogen as the carrier gas. NMR spectra were recorded using Bruker Advance III 300 MHz, Advance III 400 MHz, or Advance III HD-500 MHz spectrometers at 293 K. ^{1}H NMR chemical shifts are referenced to the residual hydrogen signal of the deuterated solvent, and the ^{13}C NMR chemical shifts are referenced to the ^{13}C signal of the deuterated solvent. Abbreviations used in the description of the NMR data are as follows: Ar, aryl; br, broad; s, singlet; d, doublet; t,triplet; q, quartet; m, multiplet. Analytical TLC was performed on Merck silica gel 60 F254 plates. Flash chromatography columns were packed with 200-300 mesh silica gel.

2. Effect of base amount and strength

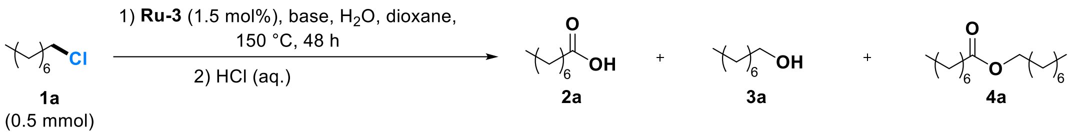

entry	base	base amount (mmol)	conversion (%)	yield (%) 2a	3a	4a
1	NaOH	2.0	>99	99	<1	<1
2	NaOH	1.5	>99	92	8	<1
3[a]	NaOH	1.5	>99	99	<1	<1
4[b]	NaOH	1.2	>99	59	9	32
5	NaOH	1.0	96	45	17	34
6	Na_2CO_3	2.0	>99	80	20	<1
7	K_3PO_4	2.0	96	81	6	8
8	$NaHCO_3$	2.0	>99	40	60	<1

[a]96 h. [b]144 h

Reactions were conducted using 0.5 mmol of **1a**, 1.5 mol% **Ru-3**, and base in 1,4-dioxane (2 mL)/water (2 mL), heated in a sealed tube at 150 °C (silicon oil bath temperature) for 48 h. Conversions and yields were determined by ^{1}H NMR with dibromomethane as an internal standard.

3. Catalytic oxidation of carbon-halogen bonds

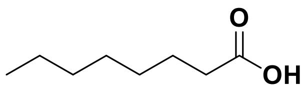

Octanoic acid (2a):[4] A 100 mL thick-glass pressure tube, equipped with a stirring bar, was charged with complex **Ru-3** (4.5 mg, 0.0075 mmol), NaOH (80.0 mg, 2.0 mmol), 1-chlorooctane (74.3 mg, 0.50 mmol), 2.0 mL of dioxane, and 2.0 mL of water. The tube was sealed, and the reaction mixture was stirred and heated at 150 °C (silicon oil bath temperature, solvent reflux). After 48 h, the reaction mixture was cooled down to room temperature and the generated gas was carefully released in a hood. Saturated brine (5.0 mL) was then added to the reaction mixture, and the mixture was extracted with ethyl acetate (3 × 5.0 mL). The aqueous phase was then acidified with 4 M HCl (4.0 mL) and extracted with ethyl acetate (3 × 5.0 mL). The combined organic extracts from the acidified aqueous phase were dried over Na_2SO_4 and all volatiles were removed under vacuum. The product was obtained as a yellow oil in 96% yield (69.1 mg).

^{1}H NMR (300 MHz, $CDCl_3$) δ 10.98 (s, 1H), 2.34 (t, J = 7.5 Hz, 2H), 1.69 – 1.56 (m, 2H), 1.40 – 1.20 (m, 8H), 0.93 – 0.82 (m, 3H).

^{13}C NMR (75 MHz, $CDCl_3$) δ 180.91, 34.47, 31.97, 29.36, 29.24, 25.02, 22.93, 14.38.

Determination of H_2 generated during the reaction: After cooling the reaction mixture to room temperature, the headspace was analyzed by GC with a TCD detector, using N_2 as the carrier gas. As shown in Figure S1, only H_2 was detected by GC while no other gases were present in detectable amounts.

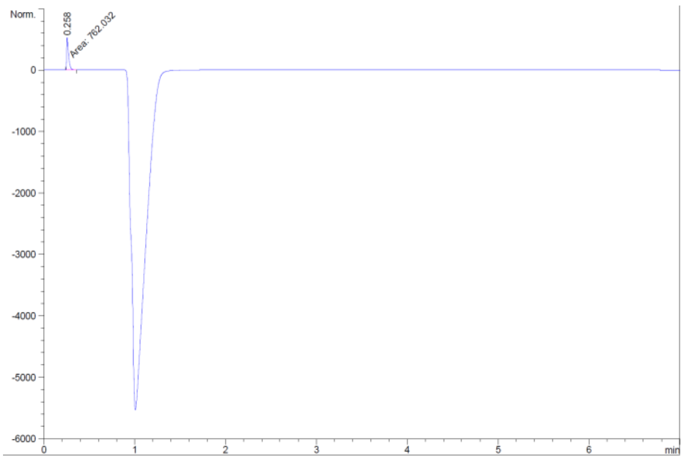

Figure S1. GC analysis of the gas phase (t = 0.258 min [H_2, 100%])

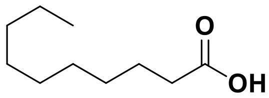

Decanoic acid (2b):[11] A 100 mL thick-glass pressure tube, equipped with a stirring bar, was charged with complex **Ru-3** (4.5 mg, 0.0075 mmol), NaOH (80.0 mg, 2.0 mmol), 1-bromodecane (110.6 mg, 0.50 mmol), 2.0 mL of dioxane, and 2.0 mL of water. The tube was sealed, and the reaction mixture was stirred and heated at 150 °C (silicon oil bath temperature, solvent reflux). After 48 h, the reaction mixture was cooled down to room temperature and the generated gas was carefully released in a hood. Saturated brine (5.0 mL) was then added to the reaction mixture, and the mixture was extracted with ethyl acetate (3 × 5.0 mL). The aqueous phase was then acidified with 4 M HCl (4.0 mL) and extracted with ethyl acetate (3 × 5.0 mL). The combined organic extracts from the acidified aqueous phase were dried over Na_2SO_4 and all volatiles were removed under vacuum. The product was obtained as a yellow oil in 90% yield (77.6 mg).

^{1}H NMR (300 MHz, $CDCl_3$) δ 11.34 (s, 1H),2.35 (t, J = 7.5 Hz, 2H), 1.70 – 1.56 (m, 2H), 1.40 – 1.19 (m, 12H), 0.88 (t, J = 6.7 Hz, 3H).

^{13}C NMR (75 MHz, $CDCl_3$) δ 180.58, 34.42, 32.20, 29.74, 29.59, 29.40, 25.02, 23.01, 14.44.

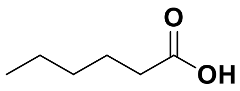

Hexanoic acid (**2c**):[4] A 100 mL thick-glass pressure tube, equipped with a stirring bar, was charged with complex **Ru-3** (4.5 mg, 0.0075 mmol), NaOH (80.0 mg, 2.0 mmol), 1-iodohexane (106.0 mg, 0.50 mmol), 2.0 mL of dioxane, and 2.0 mL of water. The tube was sealed, and the reaction mixture was stirred and heated at 150 °C (silicon oil bath temperature, solvent reflux). After 48 h, the reaction mixture was cooled down to room temperature and the generated gas was carefully released in a hood. Saturated brine (5.0 mL) was then added to the reaction mixture, and the mixture was extracted with ethyl acetate (3 × 5.0 mL). The aqueous phase was then acidified with 4 M HCl (4.0 mL) and extracted with ethyl acetate (3 × 5.0 mL). The combined organic extracts from the acidified aqueous phase were dried over Na_2SO_4 and all volatiles were removed under vacuum. The product was obtained as a yellow oil in 75% yield (43.7 mg).

^{1}H NMR (300 MHz, $CDCl_3$) δ 11.41 (s, 1H), 2.34 (t, *J* = 7.5 Hz, 2H), 1.70 – 1.56 (m, 2H), 1.40 – 1.25 (m, 4H), 0.95 – 0.84 (m, 3H).

^{13}C NMR (75 MHz, $CDCl_3$) δ 181.01, 34.44, 31.54, 24.69, 22.63, 14.18.

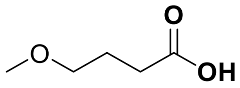

4-Methoxybutanoic acid (**2d**):[12] A 100 mL thick-glass pressure tube, equipped with a stirring bar, was charged with complex **Ru-3** (4.5 mg, 0.0075 mmol), NaOH (80.0 mg, 2.0 mmol), 1-chloro-4-methoxybutane (61.3 mg, 0.50 mmol), 2.0 mL of dioxane, and 2.0 mL of water. The tube was sealed, and the reaction mixture was stirred and heated at 150 °C (silicon oil bath temperature, solvent reflux). After 48 h, the reaction mixture was cooled down to room temperature and the generated gas was carefully released in a hood. Saturated brine (5.0 mL) was then added to the reaction mixture, and the mixture was extracted with ethyl acetate (3 × 5.0 mL). The aqueous phase was then acidified with 4 M HCl (4.0 mL) and extracted with ethyl acetate (3 × 5.0 mL). The combined organic extracts from the acidified aqueous phase were dried over Na_2SO_4 and all volatiles were removed under vacuum. The product was obtained as a yellow oil in 87% yield (51.3 mg).

^{1}H NMR (300 MHz, $CDCl_3$) δ 9.36 (s, 1H), 3.43 (t, *J* = 6.1 Hz, 2H), 3.33 (s, 3H), 2.44 (t, *J* = 7.3 Hz, 2H), 1.95 – 1.83 (m, 2H).

^{13}C NMR (75 MHz, $CDCl_3$) δ 179.61, 71.79, 58.87, 31.12, 24.90.

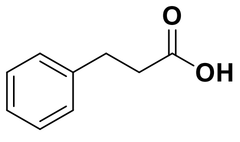

3-Phenylpropanoic acid (**2e**):[13] A 100 mL thick-glass pressure tube, equipped with a stirring bar, was charged with complex **Ru-3** (4.5 mg, 0.0075 mmol), NaOH (80.0 mg, 2.0 mmol), (3-chloropropyl)benzene (77.3 mg, 0.50 mmol), 2.0 mL of dioxane, and 2.0 mL of water. The tube was sealed, and the reaction mixture was stirred and heated at 150 °C (silicon oil bath temperature, solvent reflux). After 48 h, the reaction mixture was cooled down to room temperature and the

generated gas was carefully released in a hood. Saturated brine (5.0 mL) was then added to the reaction mixture, and the mixture was extracted with ethyl acetate (3 × 5.0 mL). The aqueous phase was then acidified with 4 M HCl (4.0 mL) and extracted with ethyl acetate (3 × 5.0 mL). The combined organic extracts from the acidified aqueous phase were dried over Na_2SO_4 and all volatiles were removed under vacuum. The product was obtained as a light-yellow solid in 83% yield (62.5 mg).

^{1}H NMR (300 MHz, $CDCl_3$) δ 11.08 (s, 1H), 7.37 – 7.28 (m, 2H), 7.28 – 7.19 (m, 3H), 2.99 (t, *J* = 7.8 Hz, 2H), 2.71 (t, *J* = 7.8 Hz, 2H).

^{13}C NMR (75 MHz, $CDCl_3$) δ 179.52, 140.48, 128.91, 128.60, 126.72, 35.93, 30.91.

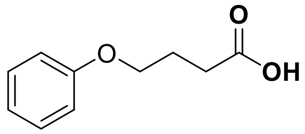

4-Phenoxybutanoic acid (**2f**):[14] A 100 mL thick-glass pressure tube, equipped with a stirring bar, was charged with complex **Ru-3** (4.5 mg, 0.0075 mmol), NaOH (80.0 mg, 2.0 mmol), (4-chlorobutoxy)benzene (92.3 mg, 0.50 mmol), 2.0 mL of dioxane, and 2.0 mL of water. The tube was sealed, and the reaction mixture was stirred and heated at 150 °C (silicon oil bath temperature, solvent reflux). After 48 h, the reaction mixture was cooled down to room temperature and the generated gas was carefully released in a hood. Saturated brine (5.0 mL) was then added to the reaction mixture, and the mixture was extracted with ethyl acetate (3 × 5.0 mL). The aqueous phase was then acidified with 4 M HCl (4.0 mL) and extracted with ethyl acetate (3 × 5.0 mL). The combined organic extracts from the acidified aqueous phase were dried over Na_2SO_4 and all volatiles were removed under vacuum. The product was obtained as a light-yellow solid in 84% yield (75.6 mg).

^{1}H NMR (300 MHz, $CDCl_3$) δ 10.81 (s, 1H), 7.31 (t, *J* = 7.9 Hz, 2H), 7.06 – 6.86 (m, 3H), 4.06 (t, *J* = 6.0 Hz, 2H), 2.63 (t, *J* = 7.2 Hz, 2H), 2.21 – 2.11 (m, 2H).

^{13}C NMR (75 MHz, $CDCl_3$) δ 180.06, 159.05, 129.79, 121.14, 114.79, 66.69, 30.94, 24.70.

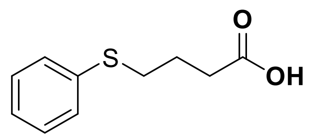

4-(Phenylthio)butanoic acid (**2g**):[15] A 100 mL thick-glass pressure tube, equipped with a stirring bar, was charged with complex **Ru-3** (4.5 mg, 0.0075 mmol), NaOH (80.0 mg, 2.0 mmol), (4-chlorobutyl)(phenyl)sulfane (100.4 mg, 0.50 mmol), 2.0 mL of dioxane, and 2.0 mL of water. The tube was sealed, and the reaction mixture was stirred and heated at 150 °C (silicon oil bath temperature, solvent reflux). After 48 h, the reaction mixture was cooled down to room temperature and the generated gas was carefully released in a hood. Saturated brine (5.0 mL) was then added to the reaction mixture, and the mixture was extracted with ethyl acetate (3 × 5.0 mL). The aqueous phase was then acidified with 4 M HCl (4.0 mL) and extracted with ethyl acetate (3 × 5.0 mL). The combined organic extracts from the acidified aqueous phase were dried over Na_2SO_4 and all volatiles were removed under vacuum. The product was obtained as a light-yellow solid in 62% yield (61.0 mg). *When the reaction was carried out for 24 h,* ***2g*** *was obtained in 61% along with 64%* H_2 *yield, and the starting material* ***1g*** *and the hydrolysis product 4-(phenylthio)butan-1-ol were not observed. Thus, the low yield should be attributable to the decomposition of* ***1g*** *during the reaction.*

^{1}H NMR (300 MHz, DMSO) δ 12.10 (s, 1H), 7.37 – 7.28 (m, 4H), 7.23 – 7.15 (m, 1H), 3.02 – 2.94 (m, 2H), 2.37 (t, *J* = 7.3 Hz, 2H), 1.79 (p, *J* = 7.3 Hz, 2H).

^{13}C NMR (75 MHz, DMSO) δ 174.84, 136.95, 129.95, 129.01, 126.53, 33.32, 32.26, 25.07.

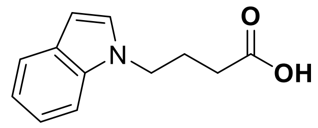

4-(1*H*-indol-1-yl)butanoic acid (**2h**):[16] A 100 mL thick-glass pressure tube, equipped with a stirring bar, was charged with complex **Ru-3** (4.5 mg, 0.0075 mmol), NaOH (80.0 mg, 2.0 mmol), 1-(4-chlorobutyl)-1*H*-indole (103.8 mg, 0.50 mmol), 2.0 mL of dioxane, and 2.0 mL of water. The tube was sealed, and the reaction mixture was stirred and heated at 150 °C (silicon oil bath temperature, solvent reflux). After 48 h, the reaction mixture was cooled down to room temperature

and the generated gas was carefully released in a hood. Saturated brine (5.0 mL) was then added to the reaction mixture, and the mixture was extracted with ethyl acetate (3 × 5.0 mL). The aqueous phase was then acidified with 4 M HCl (4.0 mL) and extracted with ethyl acetate (3 × 5.0 mL). The combined organic extracts from the acidified aqueous phase were dried over Na_2SO_4 and all volatiles were removed under vacuum. The product was obtained as a yellow solid in 85% yield (86.6 mg).

^{1}H NMR (300 MHz, DMSO) δ 12.15 (s, 1H), 7.57 (d, *J* = 7.7 Hz, 1H), 7.48 (d, *J* = 8.1 Hz, 1H), 7.34 (d, *J* = 2.9 Hz, 1H), 7.15 (t, *J* = 7.5 Hz, 1H), 7.04 (t, *J* = 7.3 Hz, 1H), 6.45 (d, *J* = 2.8 Hz, 1H), 4.20 (t, *J* = 6.9 Hz, 2H), 2.22 (t, *J* = 7.1 Hz, 2H), 2.00 (t, *J* = 6.8 Hz, 2H).

^{13}C NMR (75 MHz, DMSO) δ 174.83, 136.59, 129.38, 129.06, 121.94, 121.38, 119.83, 110.57, 101.52, 45.61, 31.63, 26.27.

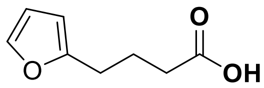

4-(Furan-2-yl)butanoic acid (**2i**):[24] A 100 mL thick-glass pressure tube, equipped with a stirring bar, was charged with complex **Ru-3** (4.5 mg, 0.0075 mmol), NaOH (80.0 mg, 2.0 mmol), 2-(4-chlorobutyl)furan (79.3 mg, 0.50 mmol), 2.0 mL of dioxane, and 2.0 mL of water. The tube was sealed, and the reaction mixture was stirred and heated at 150 °C (silicon oil bath temperature, solvent reflux). After 48 h, the reaction mixture was cooled down to room temperature and the generated gas was carefully released in a hood. Saturated brine (5.0 mL) was then added to the reaction mixture, and the mixture was extracted with ethyl acetate (3 × 5.0 mL). The aqueous phase was then acidified with 4 M HCl (4.0 mL) and extracted with ethyl acetate (3 × 5.0 mL). The combined organic extracts from the acidified aqueous phase were dried over Na_2SO_4 and all volatiles were removed under vacuum. The product was obtained as a light-yellow oil in 94% yield (72.5 mg).

^{1}H NMR (300 MHz, $CDCl_3$) δ 7.30 (d, *J* = 0.9 Hz, 1H), 6.28 (dd, *J* = 2.7, 1.9 Hz, 1H), 6.02 (d, *J* = 2.5 Hz, 1H), 2.70 (t, *J* = 7.3 Hz, 2H), 2.41 (t, *J* = 7.4 Hz, 2H), 2.05 – 1.93 (m, 2H).

^{13}C NMR (75 MHz, $CDCl_3$) δ 179.95, 154.96, 141.23, 110.24, 105.58, 33.28, 27.23, 23.18.

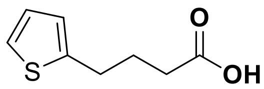

4-(Thiophen-2-yl)butanoic acid (**2j**):[25] A 100 mL thick-glass pressure tube, equipped with a stirring bar, was charged with complex **Ru-3** (4.5 mg, 0.0075 mmol), NaOH (80.0 mg, 2.0 mmol), 2-(4-chlorobutyl)thiophene (87.3 mg, 0.50 mmol), 2.0 mL of dioxane, and 2.0 mL of water. The tube was sealed, and the reaction mixture was stirred and heated at 150 °C (silicon oil bath temperature, solvent reflux). After 48 h, the reaction mixture was cooled down to room temperature and the generated gas was carefully released in a hood. Saturated brine (5.0 mL) was then added to the reaction mixture, and the mixture was extracted with ethyl acetate (3 × 5.0 mL). The aqueous phase was then acidified with 4 M HCl (4.0 mL) and extracted with ethyl acetate (3 × 5.0 mL). The combined organic extracts from the acidified aqueous phase were dried over Na_2SO_4 and all volatiles were removed under vacuum. The product was obtained as a yellow oil in 90% yield (77.0 mg).

^{1}H NMR (400 MHz, $CDCl_3$) δ 10.26 (s, 1H), 7.14 (dd, *J* = 5.1, 0.8 Hz, 1H), 6.93 (dd, *J* = 5.0, 3.5 Hz, 1H), 6.82 (d, *J* = 2.7 Hz, 1H), 2.91 (t, *J* = 7.4 Hz, 2H), 2.43 (t, *J* = 7.4 Hz, 2H), 2.03 (p, *J* = 7.4 Hz, 2H).

^{13}C NMR (101 MHz, $CDCl_3$) δ 179.92, 143.95, 126.93, 124.76, 123.46, 33.20, 29.10, 26.61.

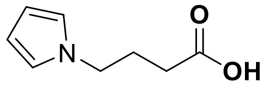

4-(1*H*-Pyrrol-1-yl)butanoic acid (**2k**):[26] A 100 mL thick-glass pressure tube, equipped with a stirring bar, was charged with complex **Ru-3** (4.5 mg, 0.0075 mmol), NaOH (80.0 mg, 2.0 mmol), 1-(4-chlorobutyl)-1*H*-pyrrole (78.8 mg, 0.50 mmol), 2.0 mL of dioxane, and 2.0 mL of water. The tube was sealed, and the reaction mixture was stirred and heated at 150 °C (silicon oil bath temperature, solvent reflux). After 48 h, the reaction mixture was cooled down to room temperature and the generated gas was carefully released in a hood. Saturated brine (5.0 mL) was then added to the reaction mixture, and the mixture was extracted with ethyl acetate (3 × 5.0 mL). The aqueous phase was then acidified with 4 M HCl (4.0 mL) and extracted with ethyl acetate (3 × 5.0 mL). The

combined organic extracts from the acidified aqueous phase were dried over Na_2SO_4 and all volatiles were removed under vacuum. The product was obtained as a yellow oil in 82% yield (62.9 mg).

^{1}H NMR (400 MHz, $CDCl_3$) δ 9.71 (s, 1H), 6.70 – 6.59 (m, 2H), 6.14 (t, *J* = 2.0 Hz, 2H), 3.95 (t, *J* = 6.8 Hz, 2H), 2.32 (t, *J* = 7.2 Hz, 2H), 2.12 – 2.04 (m, 2H).

^{13}C NMR (101 MHz, $CDCl_3$) δ 179.29, 120.65, 108.42, 48.40, 30.85, 26.54.

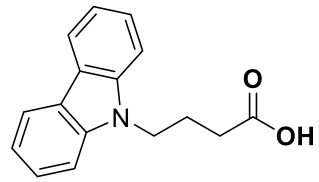

4-(9*H*-Carbazol-9-yl)butanoic acid (2l):[27] A 100 mL thick-glass pressure tube, equipped with a stirring bar, was charged with complex **Ru-3** (4.5 mg, 0.0075 mmol), NaOH (80.0 mg, 2.0 mmol), 9-(4-chlorobutyl)-9*H*-carbazole (128.9 mg, 0.50 mmol), 2.0 mL of dioxane, and 2.0 mL of water. The tube was sealed, and the reaction mixture was stirred and heated at 150 °C (silicon oil bath temperature, solvent reflux). After 48 h, the reaction mixture was cooled down to room temperature and the generated gas was carefully released in a hood. Saturated brine (5.0 mL) was then added to the reaction mixture, and the mixture was extracted with ethyl acetate (3 × 5.0 mL). The aqueous phase was then acidified with 4 M HCl (4.0 mL) and extracted with ethyl acetate (3 × 5.0 mL). The combined organic extracts from the acidified aqueous phase were dried over Na_2SO_4 and all volatiles were removed under vacuum. The product was obtained as a yellow solid in 63% yield (79.8 mg).

^{1}H NMR (400 MHz, $CDCl_3$) δ 8.09 (d, *J* = 7.8 Hz, 2H), 7.49 – 7.38 (m, 4H), 7.23 (t, *J* = 7.3 Hz, 2H), 4.38 (t, *J* = 7.0 Hz, 2H), 2.40 (t, *J* = 7.1 Hz, 2H), 2.20 (p, *J* = 7.0 Hz, 2H).

^{13}C NMR (101 MHz, $CDCl_3$) δ 179.06, 140.41, 125.92, 123.05, 120.55, 119.17, 108.64, 41.94, 31.15, 23.87.

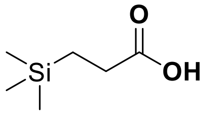

3-(Trimethylsilyl)propanoic acid (**2m**):[28] A 100 mL thick-glass pressure tube, equipped with a stirring bar, was charged with complex **Ru-3** (4.5 mg, 0.0075 mmol), NaOH (80.0 mg, 2.0 mmol), (3-chloropropyl)trimethylsilane (75.4 mg, 0.50 mmol), 2.0 mL of dioxane, and 2.0 mL of water. The tube was sealed, and the reaction mixture was stirred and heated at 150 °C (silicon oil bath temperature, solvent reflux). After 48 h, the reaction mixture was cooled down to room temperature and the generated gas was carefully released in a hood. Saturated brine (5.0 mL) was then added to the reaction mixture, and the mixture was extracted with ethyl acetate (3 × 5.0 mL). The aqueous phase was then acidified with 4 M HCl (4.0 mL) and extracted with ethyl acetate (3 × 5.0 mL). The combined organic extracts from the acidified aqueous phase were dried over Na_2SO_4 and all volatiles were removed under vacuum. The product was obtained as a yellow oil in 72% yield (52.6 mg).

^{1}H NMR (400 MHz, $CDCl_3$) δ 2.37 – 2.29 (m, 2H), 0.88 – 0.83 (m, 2H), 0.02 (s, 9H).

^{13}C NMR (101 MHz, $CDCl_3$) δ 181.16, 28.79, 11.61, -1.85.

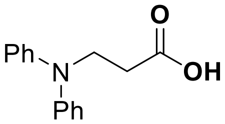

3-(Diphenylamino)propanoic acid (**2n**): A 100 mL thick-glass pressure tube, equipped with a stirring bar, was charged with complex **Ru-3** (4.5 mg, 0.0075 mmol), NaOH (80.0 mg, 2.0 mmol), *N*-(3-chloropropyl)-*N*-phenylaniline (122.9 mg, 0.50 mmol), 2.0 mL of dioxane, and 2.0 mL of water. The tube was sealed, and the reaction mixture was stirred and heated at 150 °C (silicon oil bath temperature, solvent reflux). After 48 h, the reaction mixture was cooled down to room temperature and the generated gas was carefully released in a hood. Saturated brine (5.0 mL) was then added to the reaction mixture, and the mixture was extracted with ethyl acetate (3 × 5.0 mL). The aqueous phase was then acidified with 4 M HCl (4.0 mL) and extracted with ethyl acetate (3 × 5.0 mL). The combined organic extracts from the acidified aqueous phase were dried over Na_2SO_4 and all volatiles were removed under vacuum. The product was obtained as a yellow solid in 85%

yield (102.4 mg).

^{1}H NMR (400 MHz, $CDCl_3$) δ 7.31 – 7.26 (m, 4H), 7.03 – 6.95 (m, 6H), 4.10 – 4.01 (m, 2H), 2.76 – 2.68 (m, 2H).

^{13}C NMR (101 MHz, $CDCl_3$) δ 178.30, 147.40, 129.57, 121.98, 121.22, 47.72, 32.43.

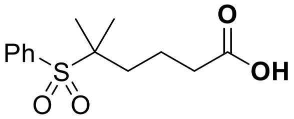

5-Methyl-5-(phenylsulfonyl)hexanoic acid (**2o**): A 100 mL thick-glass pressure tube, equipped with a stirring bar, was charged with complex **Ru-3** (4.5 mg, 0.0075 mmol), NaOH (80.0 mg, 2.0 mmol), ((6-chloro-2-methylhexan-2-yl)sulfonyl)benzene (137.4 mg, 0.50 mmol), 2.0 mL of dioxane, and 2.0 mL of water. The tube was sealed, and the reaction mixture was stirred and heated at 150 °C (silicon oil bath temperature, solvent reflux). After 48 h, the reaction mixture was cooled down to room temperature and the generated gas was carefully released in a hood. Saturated brine (5.0 mL) was then added to the reaction mixture, and the mixture was extracted with ethyl acetate (3 × 5.0 mL). The aqueous phase was then acidified with 4 M HCl (4.0 mL) and extracted with ethyl acetate (3 × 5.0 mL). The combined organic extracts from the acidified aqueous phase were dried over Na_2SO_4 and all volatiles were removed under vacuum. The product was obtained as a yellow solid in 78% yield (105.3 mg).

^{1}H NMR (400 MHz, DMSO) δ 7.84 – 7.74 (m, 3H), 7.67 (t, *J* = 7.7 Hz, 2H), 2.21 (t, *J* = 6.3 Hz, 2H), 1.62 – 1.49 (m, 4H), 1.19 (s, 6H).

^{13}C NMR (101 MHz, DMSO) δ 174.12, 134.98, 134.02, 130.20, 129.16, 62.12, 33.85, 33.74, 20.31, 18.99.

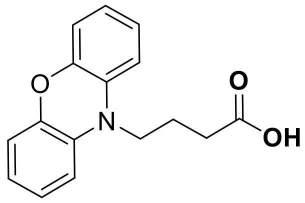

4-(10*H*-Phenoxazin-10-yl)butanoic acid (**2p**): A 100 mL thick-glass pressure tube, equipped with

a stirring bar, was charged with complex **Ru-3** (4.5 mg, 0.0075 mmol), NaOH (80.0 mg, 2.0 mmol), 10-(4-chlorobutyl)-10*H*-phenoxazine (136.9 mg, 0.50 mmol), 2.0 mL of dioxane, and 2.0 mL of water. The tube was sealed, and the reaction mixture was stirred and heated at 150 °C (silicon oil bath temperature, solvent reflux). After 48 h, the reaction mixture was cooled down to room temperature and the generated gas was carefully released in a hood. Saturated brine (5.0 mL) was then added to the reaction mixture, and the mixture was extracted with ethyl acetate (3 × 5.0 mL). The aqueous phase was then acidified with 4 M HCl (4.0 mL) and extracted with ethyl acetate (3 × 5.0 mL). The combined organic extracts from the acidified aqueous phase were dried over Na_2SO_4 and all volatiles were removed under vacuum. The product was obtained as a yellow solid in 86% yield (115.9 mg).

^{1}H NMR (400 MHz, $CDCl_3$) δ 6.84 – 6.77 (m, 2H), 6.71 – 6.61 (m, 4H), 6.55 (d, *J* = 7.9 Hz, 2H), 3.59 (s, 2H), 2.54 (t, *J* = 6.9 Hz, 2H), 2.05 – 1.95 (m, 2H).

^{13}C NMR (101 MHz, $CDCl_3$) δ 179.06, 145.07, 133.23, 123.84, 121.13, 115.61, 111.39, 43.14, 30.98, 19.95.

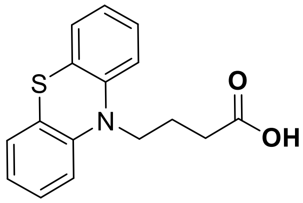

4-(10*H*-Phenothiazin-10-yl)butanoic acid (2q):[29] A 100 mL thick-glass pressure tube, equipped with a stirring bar, was charged with complex **Ru-3** (4.5 mg, 0.0075 mmol), NaOH (80.0 mg, 2.0 mmol), 10-(4-chlorobutyl)-10*H*-phenothiazine (144.9 mg, 0.50 mmol), 2.0 mL of dioxane, and 2.0 mL of water. The tube was sealed, and the reaction mixture was stirred and heated at 150 °C (silicon oil bath temperature, solvent reflux). After 48 h, the reaction mixture was cooled down to room temperature and the generated gas was carefully released in a hood. Saturated brine (5.0 mL) was then added to the reaction mixture, and the mixture was extracted with ethyl acetate (3 × 5.0 mL). The aqueous phase was then acidified with 4 M HCl (4.0 mL) and extracted with ethyl acetate (3 × 5.0 mL). The combined organic extracts from the acidified aqueous phase were dried over Na_2SO_4 and all volatiles were removed under vacuum. The product was obtained as a yellow solid in 83%

yield (118.2 mg).

^{1}H NMR (400 MHz, $CDCl_3$) δ 10.16 (s, 1H), 7.23 – 7.12 (m, 4H), 7.00 – 6.85 (m, 4H), 3.95 (t, *J* = 6.6 Hz, 2H), 2.52 (t, *J* = 7.1 Hz, 2H), 2.19 – 2.07 (m, 2H).

^{13}C NMR (101 MHz, $CDCl_3$) δ 179.69, 145.14, 127.66, 127.37, 125.59, 122.73, 115.62, 46.18, 31.13, 21.82.

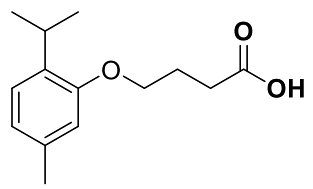

4-(2-Isopropyl-5-methylphenoxy)butanoic acid (**2r**): A 100 mL thick-glass pressure tube, equipped with a stirring bar, was charged with complex **Ru-3** (4.5 mg, 0.0075 mmol), NaOH (80.0 mg, 2.0 mmol), 2-(4-chlorobutoxy)-1-isopropyl-4-methylbenzene (120.4 mg, 0.50 mmol), 2.0 mL of dioxane, and 2.0 mL of water. The tube was sealed, and the reaction mixture was stirred and heated at 150 °C (silicon oil bath temperature, solvent reflux). After 48 h, the reaction mixture was cooled down to room temperature and the generated gas was carefully released in a hood. Saturated brine (5.0 mL) was then added to the reaction mixture, and the mixture was extracted with ethyl acetate (3 × 5.0 mL). The aqueous phase was then acidified with 4 M HCl (4.0 mL) and extracted with ethyl acetate (3 × 5.0 mL). The combined organic extracts from the acidified aqueous phase were dried over Na_2SO_4 and all volatiles were removed under vacuum. The product was obtained as a yellow solid in 82% yield (96.8 mg).

^{1}H NMR (400 MHz, $CDCl_3$) δ 7.11 (d, *J* = 7.7 Hz, 1H), 6.76 (d, *J* = 7.6 Hz, 1H), 6.66 (s, 1H), 4.03 (t, *J* = 5.9 Hz, 2H), 3.29 (dt, *J* = 13.8, 6.9 Hz, 1H), 2.64 (t, *J* = 7.3 Hz, 2H), 2.33 (s, 3H), 2.17 (dt, *J* = 13.2, 6.6 Hz, 2H), 1.22 (d, *J* = 6.9 Hz, 6H).

^{13}C NMR (101 MHz, $CDCl_3$) δ 179.83, 155.85, 136.43, 134.14, 126.00, 121.35, 112.22, 66.53, 30.94, 26.64, 24.71, 22.92, 21.46.

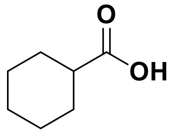

Cyclohexanecarboxylic acid (2s):[17] A 100 mL thick-glass pressure tube, equipped with a stirring bar, was charged with complex **Ru-3** (4.5 mg, 0.0075 mmol), K_2CO_3 (138.0 mg, 1.0 mmol), (bromomethyl)cyclohexane (88.5 mg, 0.50 mmol), 2.0 mL of dioxane, and 2.0 mL of water. The tube was sealed, and the reaction mixture was stirred and heated at 150 °C (silicon oil bath temperature, solvent reflux). After 48 h, the reaction mixture was cooled down to room temperature and the generated gas was carefully released in a hood. Saturated brine (5.0 mL) was then added to the reaction mixture, and the mixture was extracted with ethyl acetate (3 × 5.0 mL). The aqueous phase was then acidified with 4 M HCl (4.0 mL) and extracted with ethyl acetate (3 × 5.0 mL). The combined organic extracts from the acidified aqueous phase were dried over Na_2SO_4 and all volatiles were removed under vacuum. The product was obtained as a light-yellow semi solid in 48% yield (31.0 mg).

^{1}H NMR (300 MHz, $CDCl_3$) δ 11.18 (s, 1H), 2.33 (tt, *J* = 11.1, 3.6 Hz, 1H), 1.93 (dd, *J* = 12.8, 2.2 Hz, 2H), 1.82 – 1.70 (m, 2H), 1.69 – 1.58 (m, 1H), 1.54 – 1.36 (m, 2H), 1.36 – 1.14 (m, 3H).

^{13}C NMR (75 MHz, $CDCl_3$) δ 183.16, 43.28, 29.10, 26.02, 25.66.

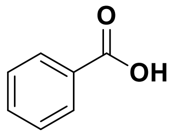

Benzoic acid (2t):[4] A 100 mL thick-glass pressure tube, equipped with a stirring bar, was charged with complex **Ru-3** (4.5 mg, 0.0075 mmol), NaOH (80.0 mg, 2.0 mmol), (chloromethyl)benzene (63.3 mg, 0.50 mmol) or (bromomethyl)benzene (85.5 mg, 0.50 mmol), 2.0 mL of dioxane, and 2.0 mL of water. The tube was sealed, and the reaction mixture was stirred and heated at 150 °C (silicon oil bath temperature, solvent reflux). After 48 h, the reaction mixture was cooled down to room temperature and the generated gas was carefully released in a hood. Saturated brine (5.0 mL) was then added to the reaction mixture, and the mixture was extracted with ethyl acetate (3 × 5.0 mL). The aqueous phase was then acidified with 4 M HCl (4.0 mL) and extracted with ethyl acetate (3 ×

5.0 mL). The combined organic extracts from the acidified aqueous phase were dried over Na_2SO_4 and all volatiles were removed under vacuum. The product was obtained as a light-yellow solid in 82% yield (50.1 mg) from (chloromethyl)benzene, in 76% yield (46.3 mg) from (bromomethyl)benzene.

^{1}H NMR (400 MHz, DMSO) δ 12.97 (s, 1H), 7.99 (d, J = 7.2 Hz, 2H), 7.66 (t, J = 7.4 Hz, 1H), 7.54 (t, J = 7.6 Hz, 2H).

^{13}C NMR (101 MHz, DMSO) δ 168.25, 133.79, 131.69, 130.20, 129.50.

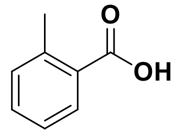

2-Methylbenzoic acid (**2u**):[18] A 100 mL thick-glass pressure tube, equipped with a stirring bar, was charged with complex **Ru-3** (4.5 mg, 0.0075 mmol), NaOH (80.0 mg, 2.0 mmol), 1-(chloromethyl)-2-methylbenzene (70.3 mg, 0.50 mmol), 2.0 mL of dioxane, and 2.0 mL of water. The tube was sealed, and the reaction mixture was stirred and heated at 150 °C (silicon oil bath temperature, solvent reflux). After 48 h, the reaction mixture was cooled down to room temperature and the generated gas was carefully released in a hood. Saturated brine (5.0 mL) was then added to the reaction mixture, and the mixture was extracted with ethyl acetate (3 × 5.0 mL). The aqueous phase was then acidified with 4 M HCl (4.0 mL) and extracted with ethyl acetate (3 × 5.0 mL). The combined organic extracts from the acidified aqueous phase were dried over Na_2SO_4 and all volatiles were removed under vacuum. The product was obtained as a light-yellow solid in 78% yield (53.2 mg).

^{1}H NMR (400 MHz, DMSO) δ 12.79 (s, 1H), 7.82 (d, J = 7.6 Hz, 2H), 7.44 (t, J = 7.3 Hz, 2H), 7.28 (t, J = 8.1 Hz, 2H), 2.52 (s, 3H).

^{13}C NMR (101 MHz, DMSO) δ 169.60, 139.91, 132.62, 132.42, 131.37, 131.10, 126.75, 22.16.

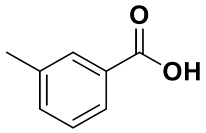

3-Methylbenzoic acid (**2v**):[17] A 100 mL thick-glass pressure tube, equipped with a stirring bar, was charged with complex **Ru-3** (4.5 mg, 0.0075 mmol), NaOH (80.0 mg, 2.0 mmol), 1-(chloromethyl)-3-methylbenzene (70.3 mg, 0.50 mmol), 2.0 mL of dioxane, and 2.0 mL of water. The tube was sealed, and the reaction mixture was stirred and heated at 150 °C (silicon oil bath temperature, solvent reflux). After 48 h, the reaction mixture was cooled down to room temperature and the generated gas was carefully released in a hood. Saturated brine (5.0 mL) was then added to the reaction mixture, and the mixture was extracted with ethyl acetate (3 × 5.0 mL). The aqueous phase was then acidified with 4 M HCl (4.0 mL) and extracted with ethyl acetate (3 × 5.0 mL). The combined organic extracts from the acidified aqueous phase were dried over Na_2SO_4 and all volatiles were removed under vacuum. The product was obtained as a light-yellow solid in 89% yield (60.5 mg).

^{1}H NMR (300 MHz, DMSO) δ 12.89 (s, 1H), 7.95 – 7.64 (m, 2H), 7.58 – 7.29 (m, 2H), 2.39 (s, 3H).

^{13}C NMR (75 MHz, DMSO) δ 168.35, 138.83, 134.38, 131.70, 130.70, 129.38, 127.42, 21.74.

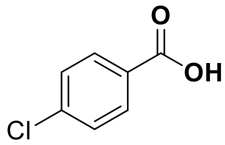

4-Chlorobenzoic acid (**2w**):[4] A 100 mL thick-glass pressure tube, equipped with a stirring bar, was charged with complex **Ru-3** (4.5 mg, 0.0075 mmol), NaOH (80.0 mg, 2.0 mmol), 1-chloro-4-(chloromethyl)benzene (80.5 mg, 0.50 mmol), 2.0 mL of dioxane, and 2.0 mL of water. The tube was sealed, and the reaction mixture was stirred and heated at 150 °C (silicon oil bath temperature, solvent reflux). After 48 h, the reaction mixture was cooled down to room temperature and the generated gas was carefully released in a hood. Saturated brine (5.0 mL) was then added to the reaction mixture, and the mixture was extracted with ethyl acetate (3 × 5.0 mL). The aqueous phase was then acidified with 4 M HCl (4.0 mL) and extracted with ethyl acetate (3 × 5.0 mL). The

combined organic extracts from the acidified aqueous phase were dried over Na_2SO_4 and all volatiles were removed under vacuum. The product was obtained as a light-yellow solid in 73% yield (57.4 mg).

^{1}H NMR (300 MHz, DMSO) δ 13.19 (s, 1H), 7.97 (d, *J* = 5.4 Hz, 2H), 7.58 (d, *J* = 5.1 Hz, 2H).

^{13}C NMR (75 MHz, DMSO) δ 167.41, 138.75, 132.08, 130.60, 129.66.

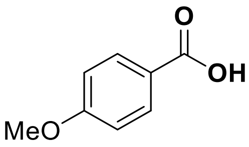

4-Methoxybenzoic acid (**2x**):[4] A 100 mL thick-glass pressure tube, equipped with a stirring bar, was charged with complex **Ru-3** (4.5 mg, 0.0075 mmol), NaOH (80.0 mg, 2.0 mmol), 1-(chloromethyl)-4-methoxybenzene (78.3 mg, 0.50 mmol), 2.0 mL of dioxane, and 2.0 mL of water. The tube was sealed, and the reaction mixture was stirred and heated at 150 °C (silicon oil bath temperature, solvent reflux). After 48 h, the reaction mixture was cooled down to room temperature and the generated gas was carefully released in a hood. Saturated brine (5.0 mL) was then added to the reaction mixture, and the mixture was extracted with ethyl acetate (3 × 5.0 mL). The aqueous phase was then acidified with 4 M HCl (4.0 mL) and extracted with ethyl acetate (3 × 5.0 mL). The combined organic extracts from the acidified aqueous phase were dried over Na_2SO_4 and all volatiles were removed under vacuum. The product was obtained as a light-yellow solid in 92% yield (70.1 mg).

^{1}H NMR (300 MHz, DMSO) δ 12.64 (s, 1H), 7.93 (d, *J* = 8.8 Hz, 2H), 7.05 (d, *J* = 8.8 Hz, 2H), 3.86 (s, 3H).

^{13}C NMR (75 MHz, DMSO) δ 167.94, 163.78, 132.28, 123.91, 114.75, 56.37.

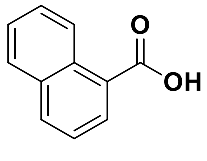

1-Naphthoic acid (**2y**):[19] A 100 mL thick-glass pressure tube, equipped with a stirring bar, was

charged with complex **Ru-3** (4.5 mg, 0.0075 mmol), NaOH (80.0 mg, 2.0 mmol), 1-(chloromethyl)naphthalene (88.3 mg, 0.50 mmol), 2.0 mL of dioxane, and 2.0 mL of water. The tube was sealed, and the reaction mixture was stirred and heated at 150 °C (silicon oil bath temperature, solvent reflux). After 48 h, the reaction mixture was cooled down to room temperature and the generated gas was carefully released in a hood. Saturated brine (5.0 mL) was then added to the reaction mixture, and the mixture was extracted with ethyl acetate (3 × 5.0 mL). The aqueous phase was then acidified with 4 M HCl (4.0 mL) and extracted with ethyl acetate (3 × 5.0 mL). The combined organic extracts from the acidified aqueous phase were dried over Na_2SO_4 and all volatiles were removed under vacuum. The product was obtained as a light-yellow solid in 64% yield (55.1 mg).

^{1}H NMR (300 MHz, DMSO) δ 13.18 (s, 1H), 8.91 (d, *J* = 8.3 Hz, 1H), 8.19 (d, *J* = 7.7 Hz, 2H), 8.10 – 8.02 (m, 1H), 7.74 – 7.57 (m, 3H).

^{13}C NMR (75 MHz, DMSO) δ 169.60, 134.41, 133.87, 131.62, 130.79, 129.55, 128.66, 128.50, 127.12, 126.43, 125.82.

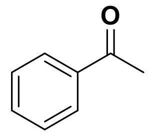

Acetophenone (**2z**):[20] A 100 mL thick-glass pressure tube, equipped with a stirring bar, was charged with complex **Ru-3** (4.5 mg, 0.0075 mmol), NaOH (24.0 mg, 1.0 mmol), (1-chloroethyl)benzene (70.3 mg, 0.50 mmol) or (1-bromoethyl)benzene (92.5 mg, 0.50 mmol), 2.0 mL of dioxane, and 0.5 mL of water. The tube was sealed, and the reaction mixture was stirred and heated at 150 °C (silicon oil bath temperature, solvent reflux). After 48 h, the reaction mixture was cooled down to room temperature and the generated gas was carefully released in a hood. 4 M HCl (4.0 mL) was then added to the reaction mixture, and the mixture was extracted with ethyl acetate (3 × 5.0 mL). Flash column chromatography (hexane: ethyl acetate = 100:1) afforded the desired product as a light-yellow oil in 85% yield (51.1 mg) from (1-chloroethyl)benzene, or 84% yield (50.5 mg) from (1-bromoethyl)benzene.

^{1}H NMR (300 MHz, $CDCl_3$) δ 7.98 – 7.91 (m, 2H), 7.59 – 7.52 (m, 1H), 7.49 – 7.42 (m, 2H), 2.60

(s, 3H).

^{13}C NMR (75 MHz, $CDCl_3$) δ 198.44, 137.42, 133.39, 128.86, 128.60, 26.89.

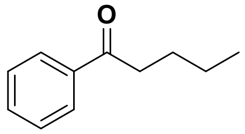

1-Phenylpentan-1-one (**2aa**):[21] A 100 mL thick-glass pressure tube, equipped with a stirring bar, was charged with complex **Ru-3** (4.5 mg, 0.0075 mmol), NaOH (40.0 mg, 1.0 mmol), (1-chloropentyl)benzene (91.3 mg, 0.50 mmol), 2.0 mL of dioxane, and 0.5 mL of water. The tube was sealed, and the reaction mixture was stirred and heated at 150 °C (silicon oil bath temperature, solvent reflux). After 48 h, the reaction mixture was cooled down to room temperature and the generated gas was carefully released in a hood. 4 M HCl (4.0 mL) was then added to the reaction mixture, and the mixture was extracted with ethyl acetate (3 × 5.0 mL). Flash column chromatography (hexane: ethyl acetate = 100:1) afforded the desired product as a yellow oil in 62% yield (50.4 mg).

^{1}H NMR (300 MHz, $CDCl_3$) δ 8.00 – 7.91 (m, 2H), 7.58 – 7.51 (m, 1H), 7.49 – 7.40 (m, 2H), 2.96 (t, J = 7.3 Hz, 2H), 1.78 – 1.66 (m, 2H), 1.48 – 1.34 (m, 2H), 0.95 (t, J = 7.3 Hz, 3H).

^{13}C NMR (75 MHz, $CDCl_3$) δ 200.90, 137.43, 133.17, 128.86, 128.37, 38.65, 26.81, 22.81, 14.26.

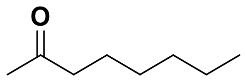

Octan-2-one (**2ab**):[22] A 100 mL thick-glass pressure tube, equipped with a stirring bar, was charged with complex **Ru-3** (4.5 mg, 0.0075 mmol), NaOH (40.0 mg, 1.0 mmol), 2-chlorooctane (74.3 mg, 0.50 mmol), 2.0 mL of dioxane, and 0.5 mL of water. The tube was sealed, and the reaction mixture was stirred and heated at 150 °C (silicon oil bath temperature, solvent reflux). After 48 h, the reaction mixture was cooled down to room temperature and the generated gas was carefully released in a hood. 4 M HCl (4.0 mL) was then added to the reaction mixture, and the mixture was extracted with ethyl acetate (3 × 5.0 mL). Flash column chromatography (hexane: ethyl acetate = 100:1)

afforded the desired product as a yellow oil in 56% yield (36.0 mg).

^{1}H NMR (300 MHz, $CDCl_3$) δ 2.39 (t, J = 7.4 Hz, 2H), 2.10 (s, 3H), 1.60-1.47 (m, 2H), 1.34 – 1.19 (m, 6H), 0.85 (t, J = 6.6 Hz, 3H).

^{13}C NMR (75 MHz, $CDCl_3$) δ 209.65, 44.10, 31.89, 30.11, 29.14, 24.13, 22.78, 14.30.

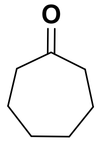

Cycloheptanone (**2ac**): A 100 mL thick-glass pressure tube, equipped with a stirring bar, was charged with complex **Ru-3** (4.5 mg, 0.0075 mmol), NaOH (40.0 mg, 1.0 mmol), bromocycloheptane (88.5 mg, 0.50 mmol), 2.0 mL of dioxane, and 0.50 mL of water. The tube was sealed, and the reaction mixture was stirred and heated at 150 °C (silicon oil bath temperature, solvent reflux). After 48 h, the reaction mixture was cooled down to room temperature and the generated gas was carefully released in a hood. 4 M HCl (4.0 mL) was then added to the reaction mixture, and the mixture was extracted with ethyl acetate (3 × 5.0 mL). The combined organic extracts were analyzed by GC (FID detector), using mesitylene as an internal standard (75% yield).

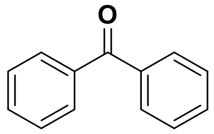

Benzophenone (**2ad**):[20] A 100 mL thick-glass pressure tube, equipped with a stirring bar, was charged with complex **Ru-3** (4.5 mg, 0.0075 mmol), NaOH (24.0 mg, 1.0 mmol), (bromomethylene)dibenzene (123.6 mg, 0.50 mmol), 2.0 mL of dioxane, and 0.5 mL of water. The tube was sealed, and the reaction mixture was stirred and heated at 150 °C (silicon oil bath temperature, solvent reflux). After 48 h, the reaction mixture was cooled down to room temperature and the generated gas was carefully released in a hood. 4 M HCl (4.0 mL) was then added to the reaction mixture, and the mixture was extracted with ethyl acetate (3 × 5.0 mL). Flash column chromatography (hexane: ethyl acetate = 100:1) afforded the desired product as a white solid in 97% yield (88.5 mg).

From (fluoromethylene)dibenzene (**1ad-F**): A 50 mL thick-glass pressure tube, equipped with a stirring bar, was charged with complex **Ru-3** (2.3 mg, 0.0038 mmol), NaOH (20.0 mg, 0.50 mmol), (fluoromethylene)dibenzene (46.6 mg, 0.25 mmol), 1.0 mL of dioxane, and 0.25 mL of water. The tube was sealed, and the reaction mixture was stirred and heated at 150 °C (silicon oil bath temperature, solvent reflux). After 72 h, the reaction mixture was cooled down to room temperature and the generated gas was carefully released in a hood. 4 M HCl (4.0 mL) was then added to the reaction mixture, and the mixture was extracted with ethyl acetate (3 × 5.0 mL). Flash column chromatography (hexane: ethyl acetate = 100:1) afforded the desired product as a white solid in 82% yield (37.2 mg).

^{1}H NMR (300 MHz, $CDCl_3$) δ 7.85 – 7.77 (m, 4H), 7.63 – 7.54 (m, 2H), 7.53 – 7.44 (m, 4H).

^{13}C NMR (75 MHz, $CDCl_3$) δ 197.07, 137.93, 132.73, 130.38, 128.60.

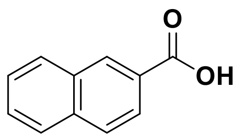

2-Naphthoic acid (**2ae**):[17] A 50 mL thick-glass pressure tube, equipped with a stirring bar, was charged with complex **Ru-3** (2.3 mg, 0.0038 mmol), NaOH (40.0 mg, 1.0 mmol), 2-(fluoromethyl)naphthalene (40.1 mg, 0.25 mmol), 1.0 mL of dioxane, and 1.0 mL of water. The tube was sealed, and the reaction mixture was stirred and heated at 150 °C (silicon oil bath temperature, solvent reflux). After 72 h, the reaction mixture was cooled down to room temperature and the generated gas was carefully released in a hood. Saturated brine (5.0 mL) was then added to the reaction mixture, and the mixture was extracted with ethyl acetate (3 × 5.0 mL). The aqueous phase was then acidified with 4 M HCl (4.0 mL) and extracted with ethyl acetate (3 × 5.0 mL). The combined organic extracts from the acidified aqueous phase were dried over Na_2SO_4 and all volatiles were removed under vacuum. The product was obtained as a light-yellow solid in 96% yield (41.4 mg).

^{1}H NMR (300 MHz, DMSO) δ 13.12 (s, 1H), 8.66 (s, 1H), 8.15 (d, J = 7.5 Hz, 1H), 8.07 – 7.99 (m, 3H), 7.73 – 7.58 (m, 2H).

^{13}C NMR (75 MHz, DMSO) δ 168.41, 135.89, 133.11, 131.48, 130.23, 129.26, 129.11, 129.04,

128.60, 127.75, 126.13.

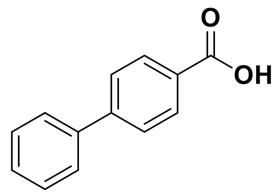

[1,1'-Biphenyl]-4-carboxylic acid (**2af**):[17] A 50 mL thick-glass pressure tube, equipped with a stirring bar, was charged with complex **Ru-3** (2.3 mg, 0.0038 mmol), NaOH (40.0 mg, 1.0 mmol), 4-(fluoromethyl)-1,1'-biphenyl (46.6 mg, 0.25 mmol), 1.0 mL of dioxane, and 1.0 mL of water. The tube was sealed, and the reaction mixture was stirred and heated at 150 °C (silicon oil bath temperature, solvent reflux). After 72 h, the reaction mixture was cooled down to room temperature and the generated gas was carefully released in a hood. Saturated brine (5.0 mL) was then added to the reaction mixture, and the mixture was extracted with ethyl acetate (3 × 5.0 mL). The aqueous phase was then acidified with 4 M HCl (4.0 mL) and extracted with ethyl acetate (3 × 5.0 mL). The combined organic extracts from the acidified aqueous phase were dried over Na_2SO_4 and all volatiles were removed under vacuum. The product was obtained as a light-yellow solid in 81% yield (40.1 mg).

^{1}H NMR (500 MHz, DMSO) δ 13.01 (s, 1H), 8.06 (d, *J* = 8.2 Hz, 2H), 7.84 (d, *J* = 8.2 Hz, 2H), 7.77 (d, *J* = 7.4 Hz, 2H), 7.54 (t, *J* = 7.5 Hz, 2H), 7.46 (t, *J* = 7.3 Hz, 1H).

^{13}C NMR (126 MHz, DMSO) δ 168.05, 145.24, 139.95, 130.88, 130.54, 130.01, 129.21, 127.89, 127.74.

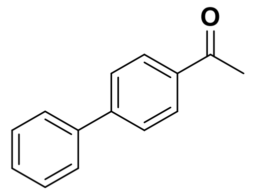

1-([1,1'-Biphenyl]-4-yl)ethan-1-one (**2ag**):[23] A 50 mL thick-glass pressure tube, equipped with a stirring bar, was charged with complex **Ru-3** (2.3 mg, 0.0038 mmol), NaOH (20.0 mg, 0.50 mmol), 4-(1-fluoroethyl)-1,1'-biphenyl (50.1 mg, 0.25 mmol), 1.0 mL of dioxane, and 0.25 mL of water. The tube was sealed, and the reaction mixture was stirred and heated at 150 °C (silicon oil bath

temperature, solvent reflux). After 72 h, the reaction mixture was cooled down to room temperature and the generated gas was carefully released in a hood. 4 M HCl (4.0 mL) was then added to the reaction mixture, and the mixture was extracted with ethyl acetate (3 × 5.0 mL). Flash column chromatography (hexane: ethyl acetate = 100:1) afforded the desired product as a white solid in 40% yield (19.7 mg).

^{1}H NMR (300 MHz, $CDCl_3$) δ 8.04 (d, J = 7.9 Hz, 2H), 7.69 (d, J = 7.9 Hz, 2H), 7.63 (d, J = 7.4 Hz, 2H), 7.48 (t, J = 7.3 Hz, 2H), 7.44 – 7.36 (m, 1H), 2.65 (s, 3H).

^{13}C NMR (75 MHz, $CDCl_3$) δ 198.00, 145.91, 139.99, 136.08, 129.15, 129.07, 128.35, 127.39, 127.35, 26.87.

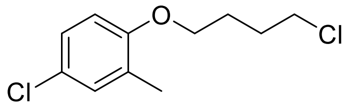

4-Chloro-1-(4-chlorobutoxy)-2-methylbenzene (1ah): A vial containing a stirring bar was charged with 4-chloro-2-methylphenol (1.43 g, 10 mmol), K_2CO_3 (1.66 g, 12 mmol) and EtOH (20 mL). After stirring for 5 min, the 1-bromo-4-chlorobutane (2.06 g, 12 mmol) was added. The mixture was then stirred and refluxed for 20 hours. Upon completion, the reaction mixture was filtered and concentrated. The crude product was purified by flash chromatography (hexane/ethyl acetate = 100:1) to give **1ah** as a colorless oil (2.24 g, 96% yield).

^{1}H NMR (300 MHz, $CDCl_3$) δ 7.13 – 7.06 (m, 2H), 6.71 (d, J = 8.5 Hz, 1H), 4.01 – 3.92 (m, 2H), 3.67 – 3.59 (m, 2H), 2.20 (s, 3H), 2.05 – 1.92 (m, 4H).

^{13}C NMR (75 MHz, $CDCl_3$) δ 155.69, 130.48, 128.74, 126.41, 125.06, 111.92, 67.38, 44.84, 29.53, 26.79, 16.22.

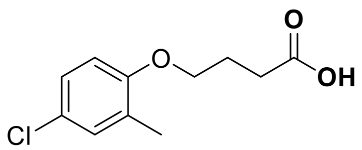

4-(4-Chloro-2-methylphenoxy)butanoic acid (2ah):[17] In a N_2-filled glovebox, a 90 mL Fischer-Porter tube was charged with **Ru-3** (30.5 mg, 0.05 mmol), **1ah** (1.17 g, 5 mmol), NaOH (800 mg,

20 mmol), H_2O (20 mL), dioxane (20 mL) and a stirring bar. The Fischer-Porter tube was sealed and taken out of the glovebox. The reaction mixture was heated to 150 °C (oil bath temperature) and stirred at this temperature for 72 h. Upon completion, the reaction solution was cooled to room temperature and the generated gas amount was measured in a gas burette. H_2 yield was calculated based on the gas collected (231 mL, 94%). H_2O (30 mL) was then added to the reaction mixture, and the mixture was extracted with diethyl ether (3 × 20 mL). The aqueous phase was then acidified with 4 M HCl (20 mL) and extracted with ethyl acetate (4 × 20 mL). The combined organic extracts from the acidified aqueous phase were dried over Na_2SO_4 and all volatiles were removed under vacuum. The product was obtained as a yellow solid (0.99 g, 87% yield).

^{1}H NMR (300 MHz, $CDCl_3$) δ 7.14 – 7.04 (m, 2H), 6.70 (d, *J* = 9.2 Hz, 1H), 3.99 (t, *J* = 5.9 Hz, 2H), 2.60 (t, *J* = 7.2 Hz, 2H), 2.23 – 2.08 (m, 5H).

^{13}C NMR (75 MHz, $CDCl_3$) δ 179.59, 155.56, 130.55, 128.80, 126.44, 125.25, 111.97, 66.91, 30.78, 24.52, 16.17.

Safety Note on Handling Hydrogen Gas After Reaction Completion

Upon completion of the scale-up reaction, a substantial amount of hydrogen gas (H_2) is generated. To prevent the formation of explosive hydrogen-oxygen mixtures, care must be taken to avoid any exposure of the evolved hydrogen to atmospheric air. Gas collection and disposal should be performed using gas-tight apparatus and under an inert atmosphere whenever possible. The reaction vessel and gas collection system should be well sealed and located in a well-ventilated area. Appropriate explosion-proof equipment and strict adherence to laboratory safety protocols are essential to ensure safe handling of hydrogen gas after the reaction.

Determination of H_2 generated during the reaction: After cooling the reaction mixture to room temperature, the headspace was analyzed by GC with a TCD detector, using N_2 as the carrier gas. As shown in Figure S2, only H_2 was detected by GC while no other gases were present in detectable amounts.

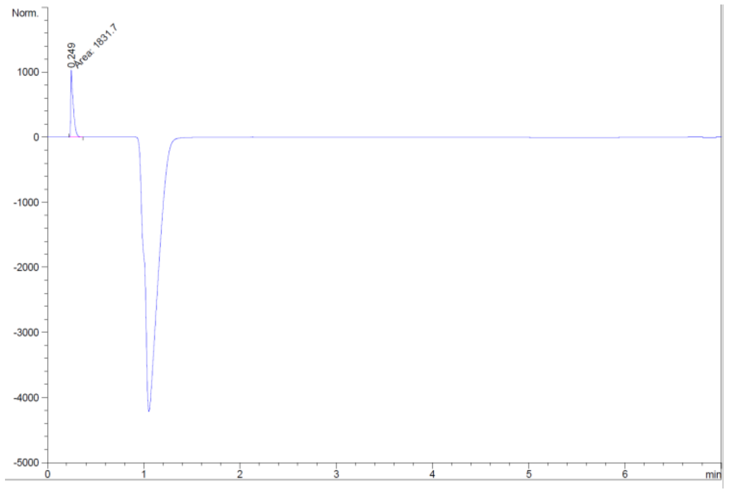

Figure S2. GC analysis of the gas phase (t = 0.249 min [H_2, 100%])

4. Anti-Markovnikov Oxidation of Nonactivated Olefins to Carboxylic Acids

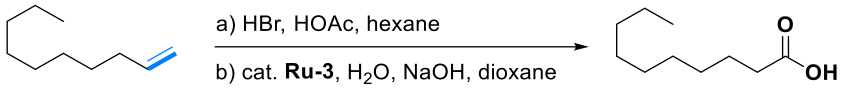

4a **2b**

A 100 mL thick-glass pressure tube equipped with a magnetic stirring bar was charged with a solution of 1-decene (70.1 mg, 0.50 mmol) in hexane (2.5 mL). The solution was stirred at 0 °C, while a gentle stream of air was bubbled through the mixture for 1 hour. Subsequently, a 33% w/v solution of HBr in acetic acid (0.19 mL, 1.0 mmol) was added via syringe. The tube was immediately sealed, and the reaction mixture was stirred at 0 °C for an additional 2 hours. Upon completion, stirring was stopped, and the volatile components were gently removed under reduced pressure. The tube was then transferred into a glovebox, where complex **Ru-3** (4.5 mg, 0.0075 mmol), NaOH (80.0 mg, 2.0 mmol), dioxane (2.0 mL), and water (2.0 mL) were added. The tube was sealed and taken out of the glovebox. The reaction mixture was then stirred and heated at 150 °C (silicon oil bath temperature, solvent reflux). After 48 h, the reaction mixture was cooled down to room temperature and the generated gas was carefully released in a hood. Saturated brine (5.0 mL) was then added to the reaction mixture, and the mixture was extracted with ethyl acetate (3 × 5.0 mL). The aqueous phase was then acidified with 4 M HCl (4.0 mL) and extracted with ethyl acetate (3 × 5.0 mL). The combined organic extracts from the acidified aqueous phase were dried over Na_2SO_4 and all volatiles were removed under vacuum. The product **2b** was obtained as a yellow oil in 84% yield (72.4 mg).

The anti-Markovnikov hydrobromination was carried out following a literature-reported protocol.[30]

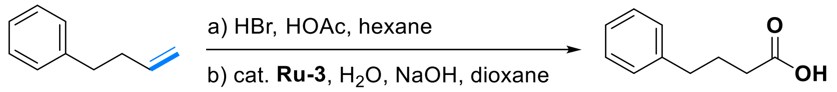

4b **2ai**

A 100 mL thick-glass pressure tube equipped with a magnetic stirring bar was charged with a solution of but-3-en-1-ylbenzene (66.1 mg, 0.50 mmol) in hexane (2.5 mL). The solution was stirred at 0 °C, while a gentle stream of air was bubbled through the mixture for 1 hour. Subsequently, a 33% w/v solution of HBr in acetic acid (0.19 mL, 1.0 mmol) was added via syringe. The tube was immediately sealed, and the reaction mixture was stirred at 0 °C for an additional 2 hours. Upon

completion, stirring was stopped, and the volatile components were gently removed under reduced pressure. The tube was then transferred into a glovebox, where complex **Ru-3** (4.5 mg, 0.0075 mmol), NaOH (80.0 mg, 2.0 mmol), dioxane (2.0 mL), and water (2.0 mL) were added. The tube was sealed and taken out of the glovebox. The reaction mixture was then stirred and heated at 150 °C (silicon oil bath temperature, solvent reflux). After 48 h, the reaction mixture was cooled down to room temperature and the generated gas was carefully released in a hood. Saturated brine (5.0 mL) was then added to the reaction mixture, and the mixture was extracted with ethyl acetate (3 × 5.0 mL). The aqueous phase was then acidified with 4 M HCl (4.0 mL) and extracted with ethyl acetate (3 × 5.0 mL). The combined organic extracts from the acidified aqueous phase were dried over Na_2SO_4 and all volatiles were removed under vacuum. The product **2ai**[31] was obtained as a yellow sticky oil in 81% yield (66.4 mg).

The anti-Markovnikov hydrobromination was carried out following a literature-reported protocol.[30]

^{1}H NMR (400 MHz, $CDCl_3$) δ 10.76 (s, 1H), 7.38 – 7.12 (m, 5H), 2.70 (t, *J* = 7.5 Hz, 2H), 2.40 (t, *J* = 7.3 Hz, 2H), 2.05 – 1.94 (m, 2H).

^{13}C NMR (101 MHz, $CDCl_3$) δ 180.30, 141.29, 128.59, 128.53, 126.16, 35.08, 33.47, 26.30.

5. Control experiments with benzyl fluoride substrate 1ae

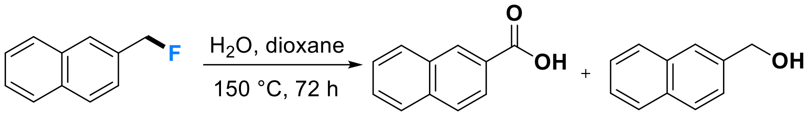

1ae **2ae** **3ae**

entry	NaOH	**Ru-4**	conv. (%)	yield (%)	
				**2ae**	**3ae**
1	4 eq	1.5 mol%	>99	98	<1
2	none	1.5 mol%	>99	<1	97
3	4 eq	none	>99	<1	99
4	none	none	>99	<1	98

Reactions were conducted using 0.5 mmol of **1ae** in 1,4-dioxane (2 mL)/water (2 mL), heated in a sealed tube at 150 °C (silicon oil bath temperature) for 72 h. Conversions and yields were determined by ^{1}H NMR with dibromomethane as an internal standard.

6. NMR spectra

2a

^{1}H NMR (300 MHz, $CDCl_3$)

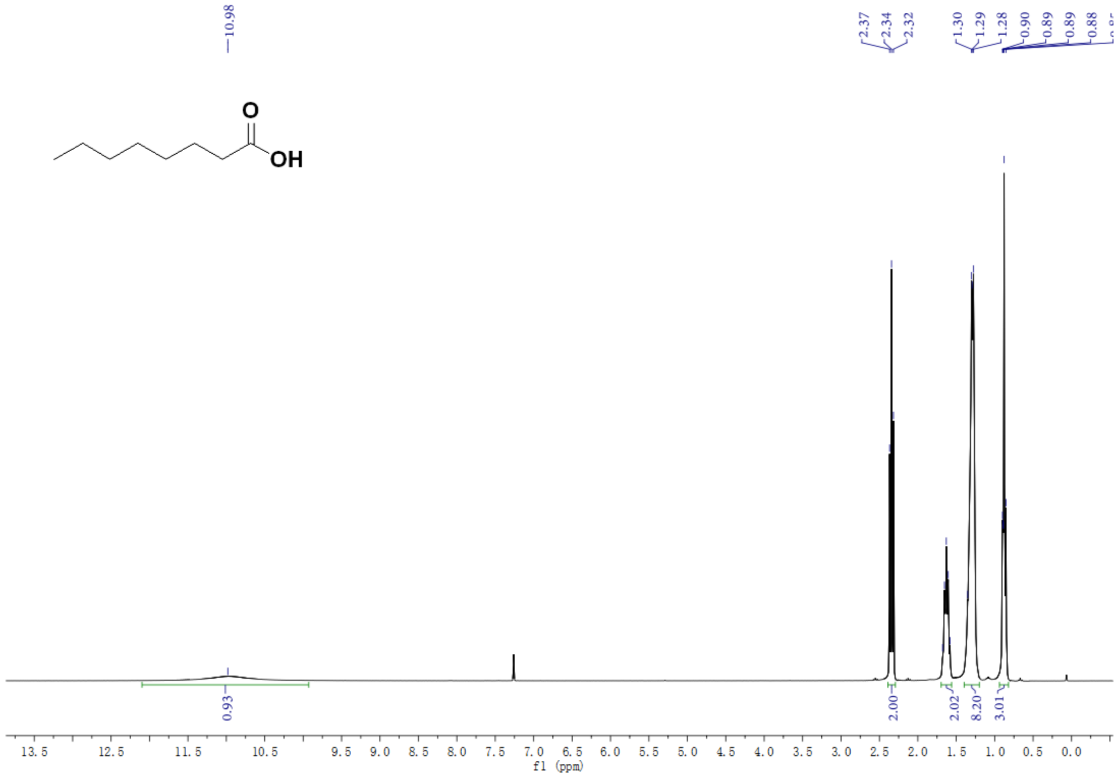

^{13}C NMR (75 MHz, $CDCl_3$)

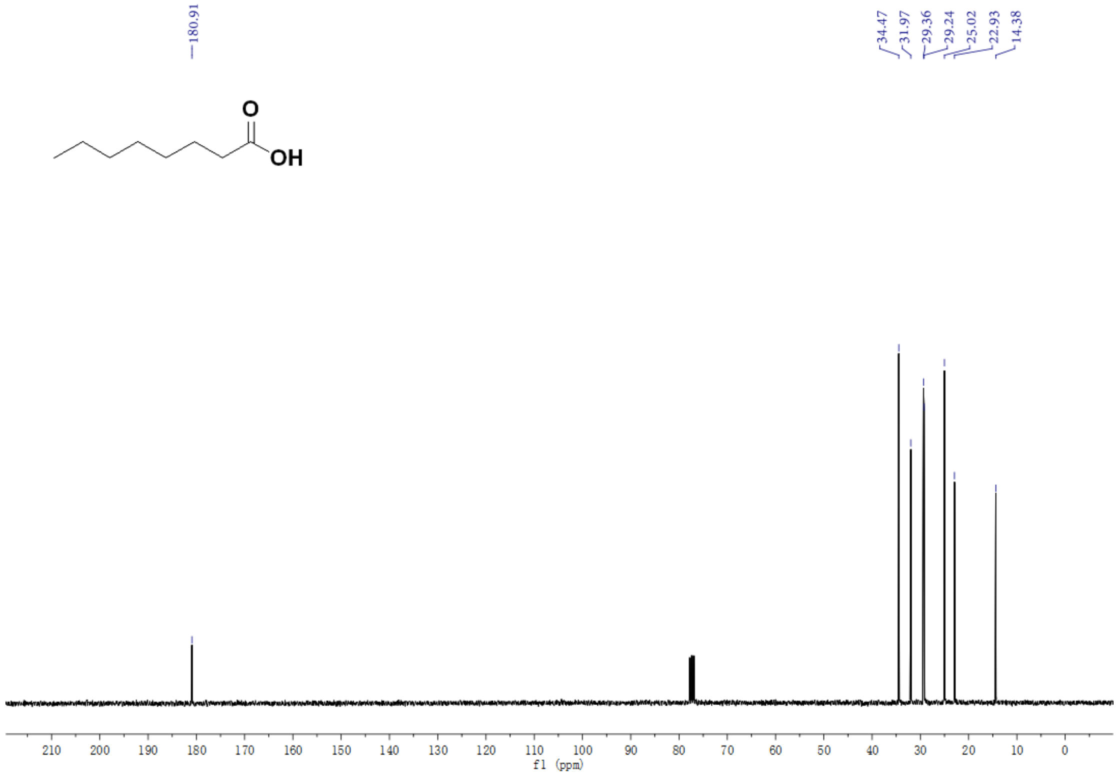

2b

^{1}H NMR (300 MHz, $CDCl_3$)

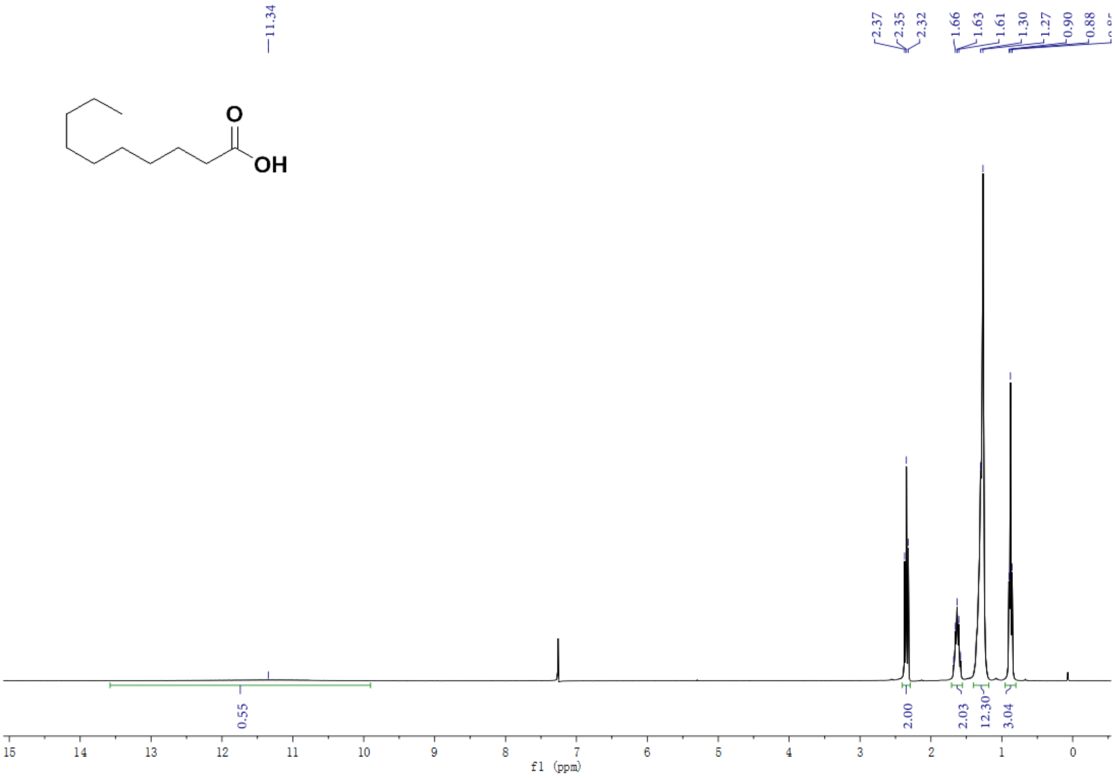

^{13}C NMR (75 MHz, $CDCl_3$)

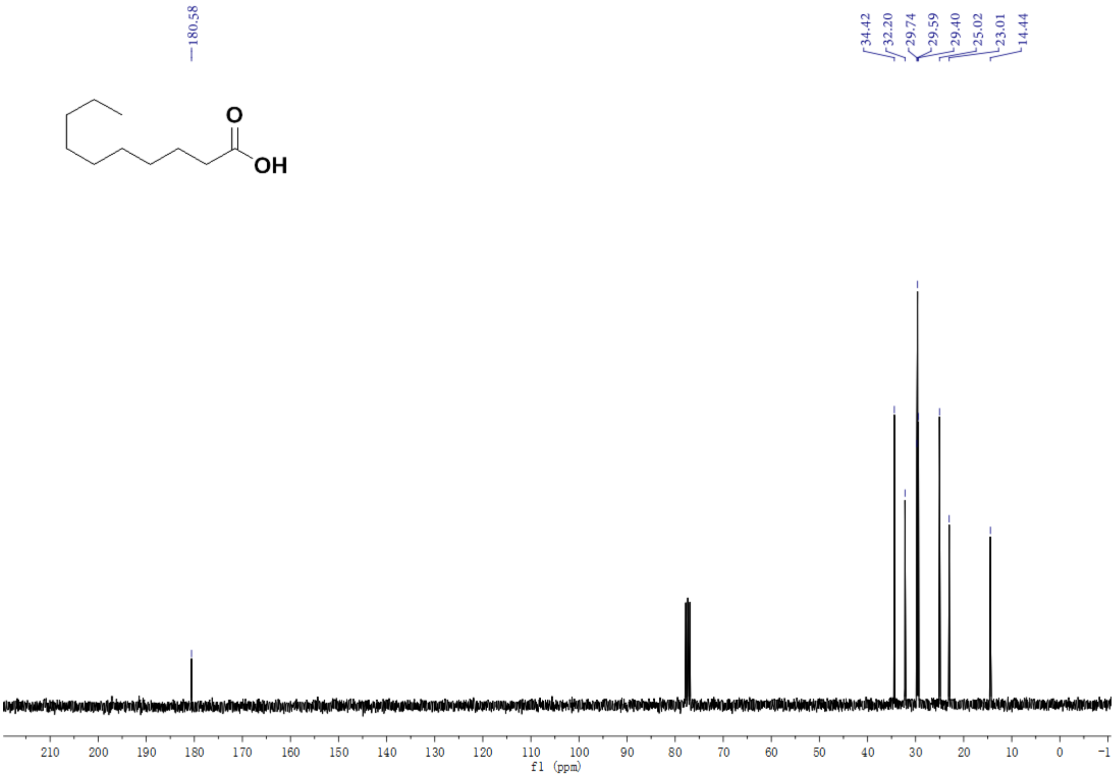

2c

^{1}H NMR (300 MHz, $CDCl_3$)

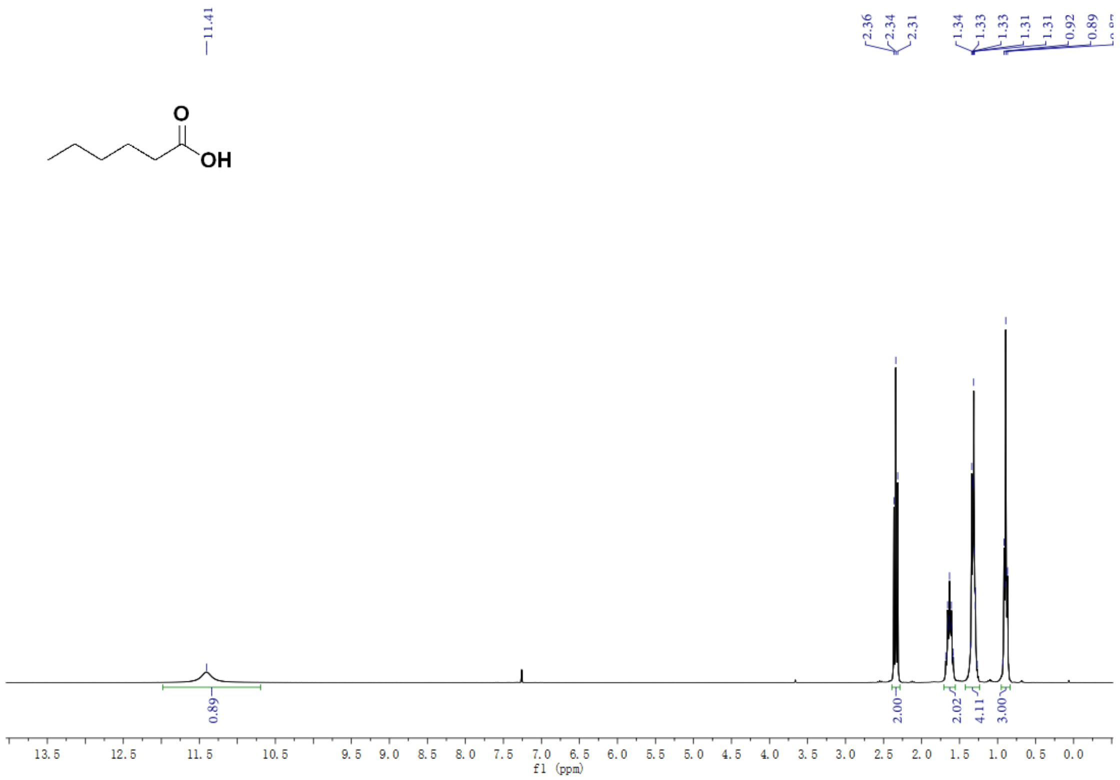

^{13}C NMR (75 MHz, $CDCl_3$)

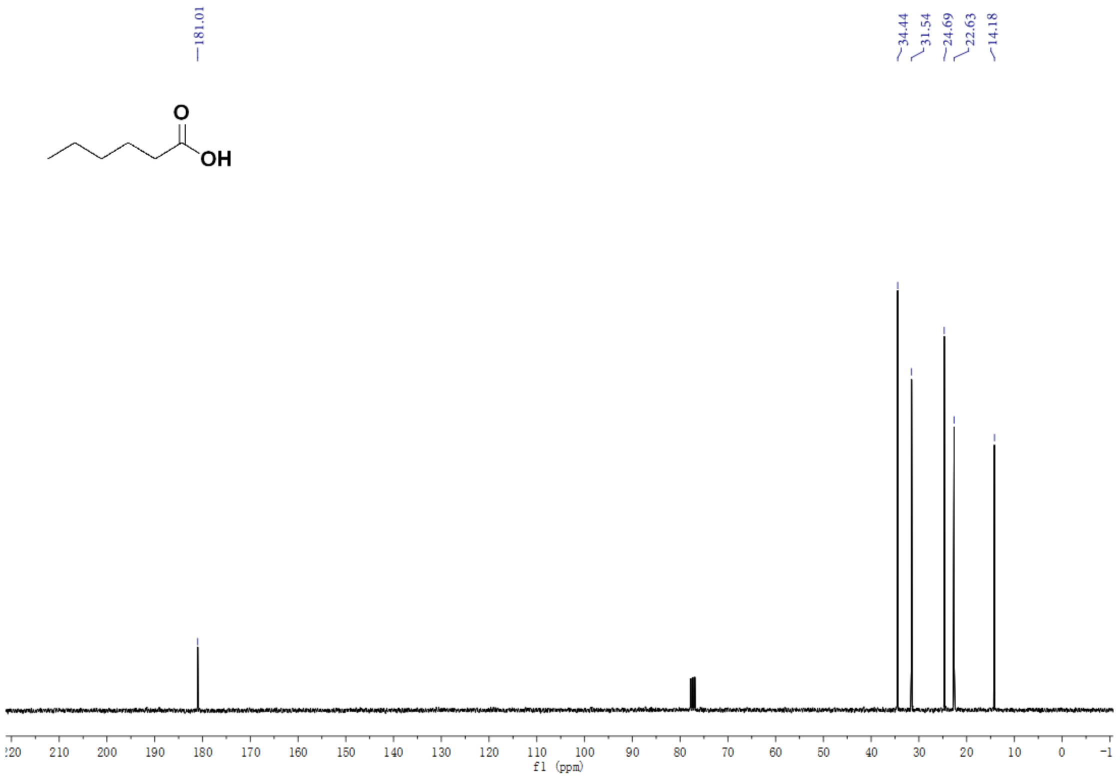

2d

^{1}H NMR (300 MHz, $CDCl_3$)

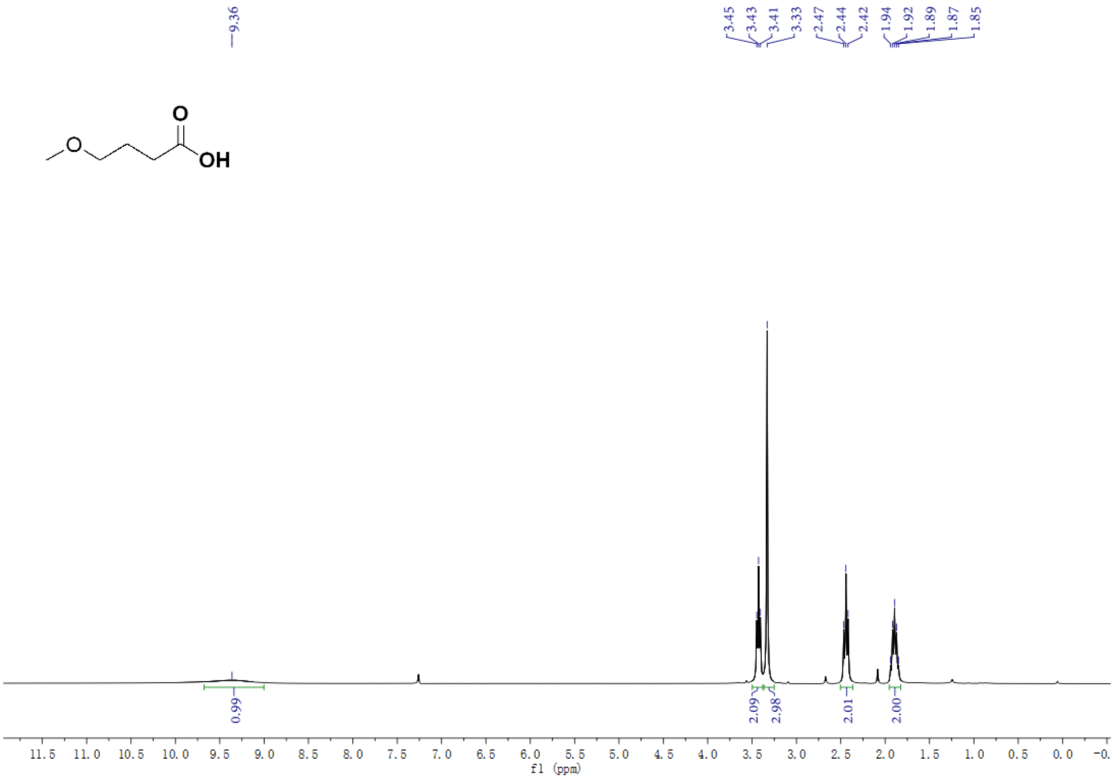

^{13}C NMR (75 MHz, $CDCl_3$)

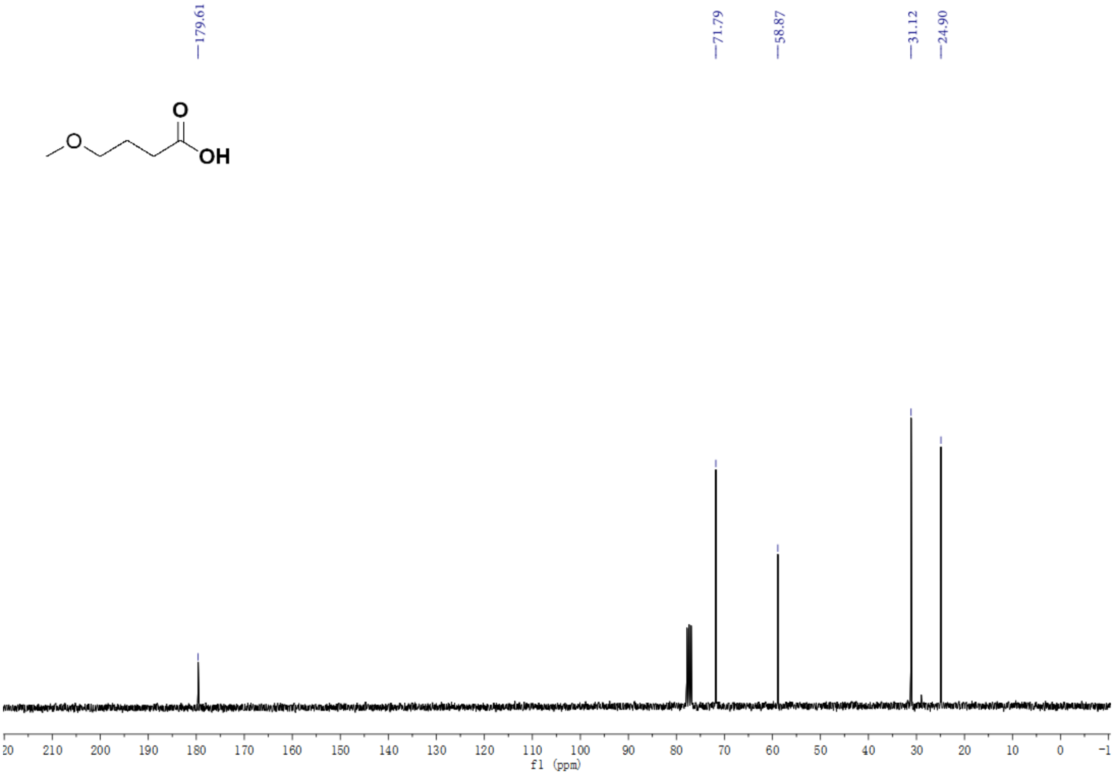

2e

^{1}H NMR (300 MHz, $CDCl_3$)

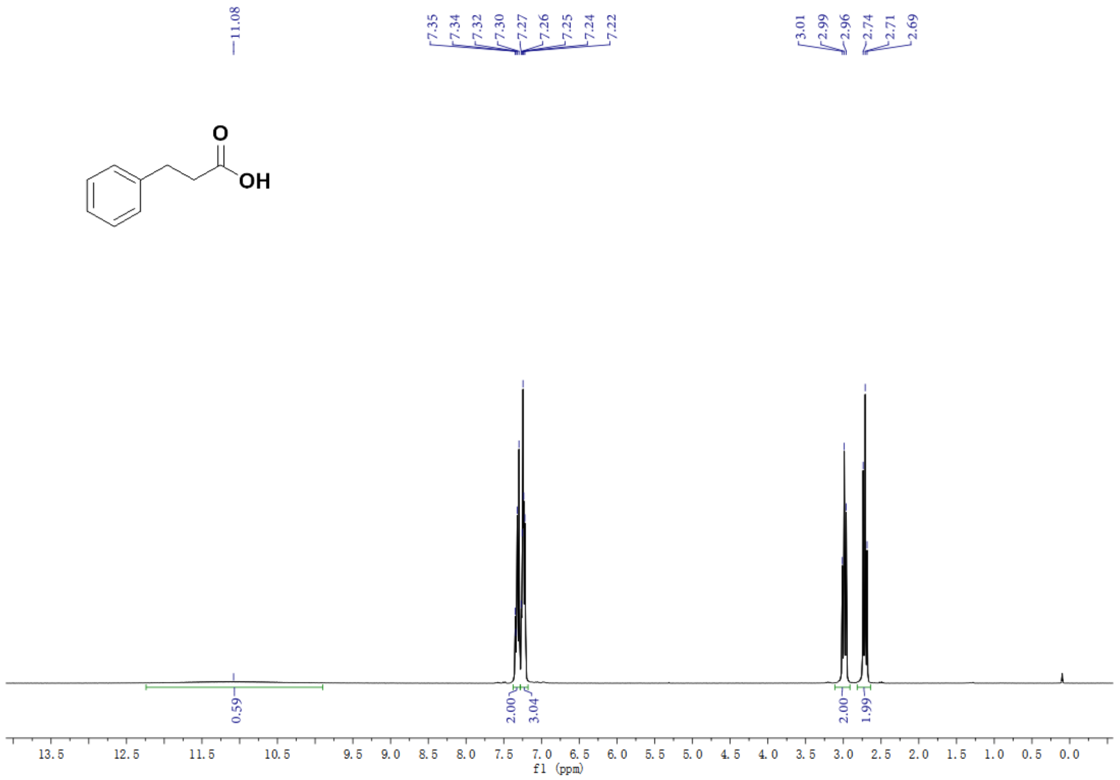

^{13}C NMR (75 MHz, $CDCl_3$)

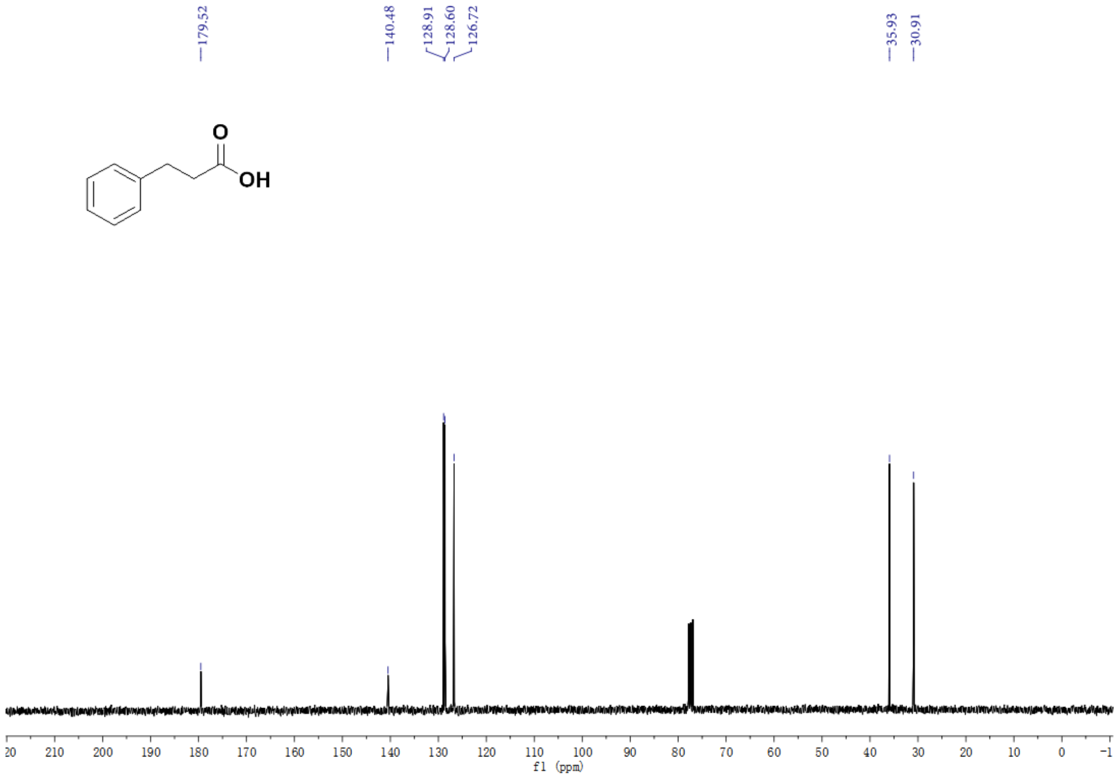

2f

^{1}H NMR (300 MHz, $CDCl_3$)

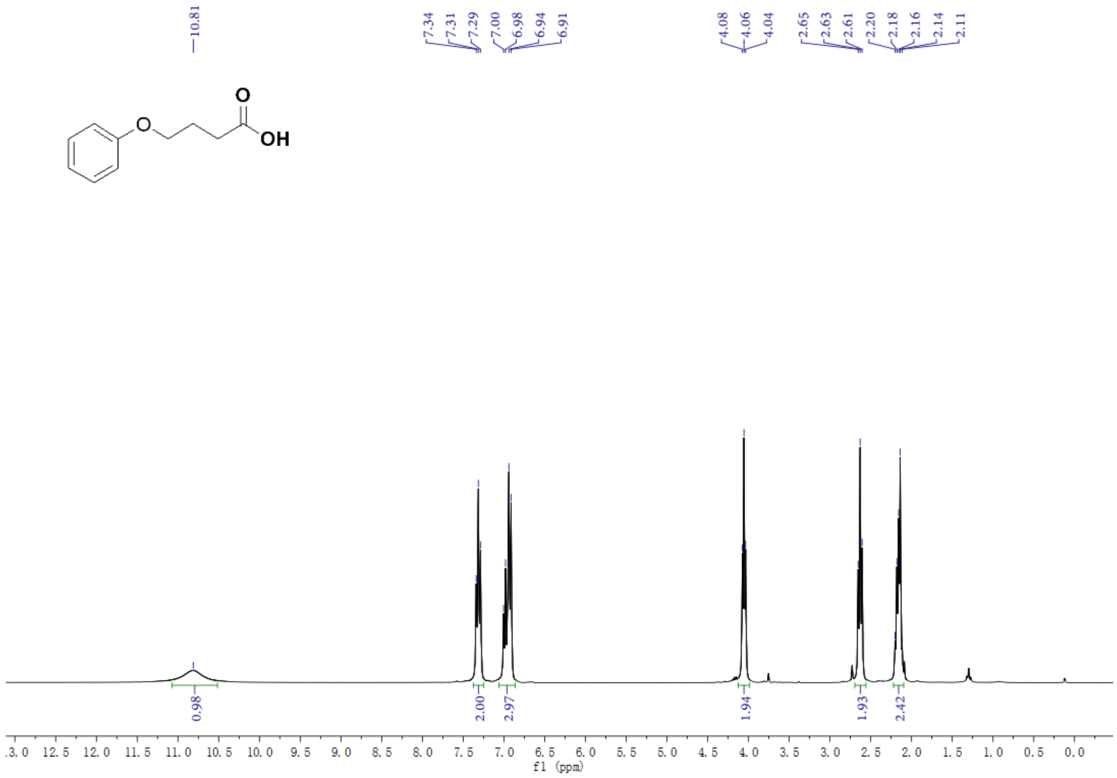

^{13}C NMR (75 MHz, $CDCl_3$)

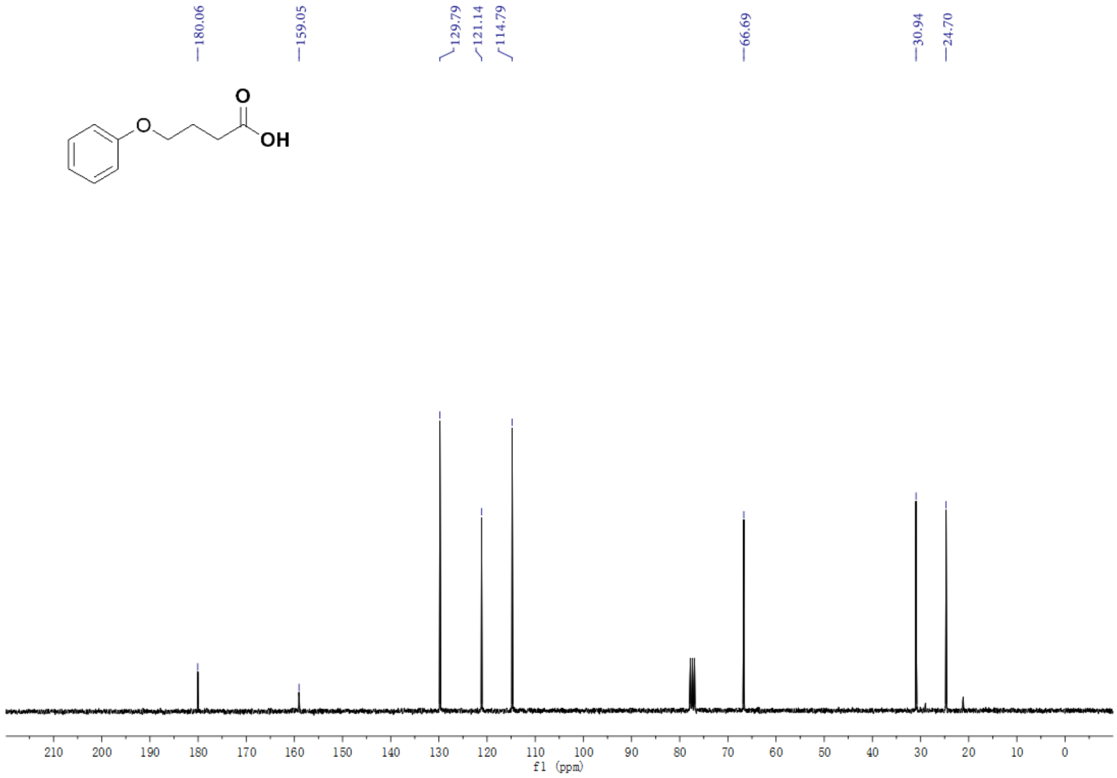

2g

^{1}H NMR (300 MHz, DMSO)

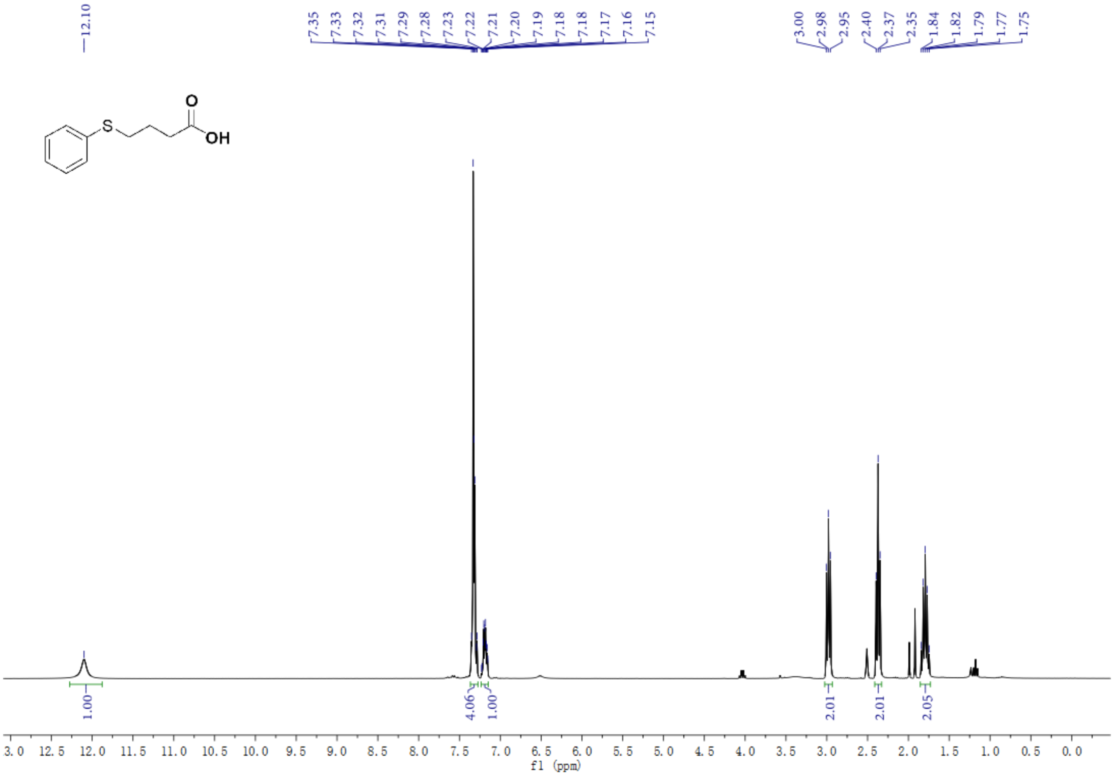

^{13}C NMR (75 MHz, DMSO)

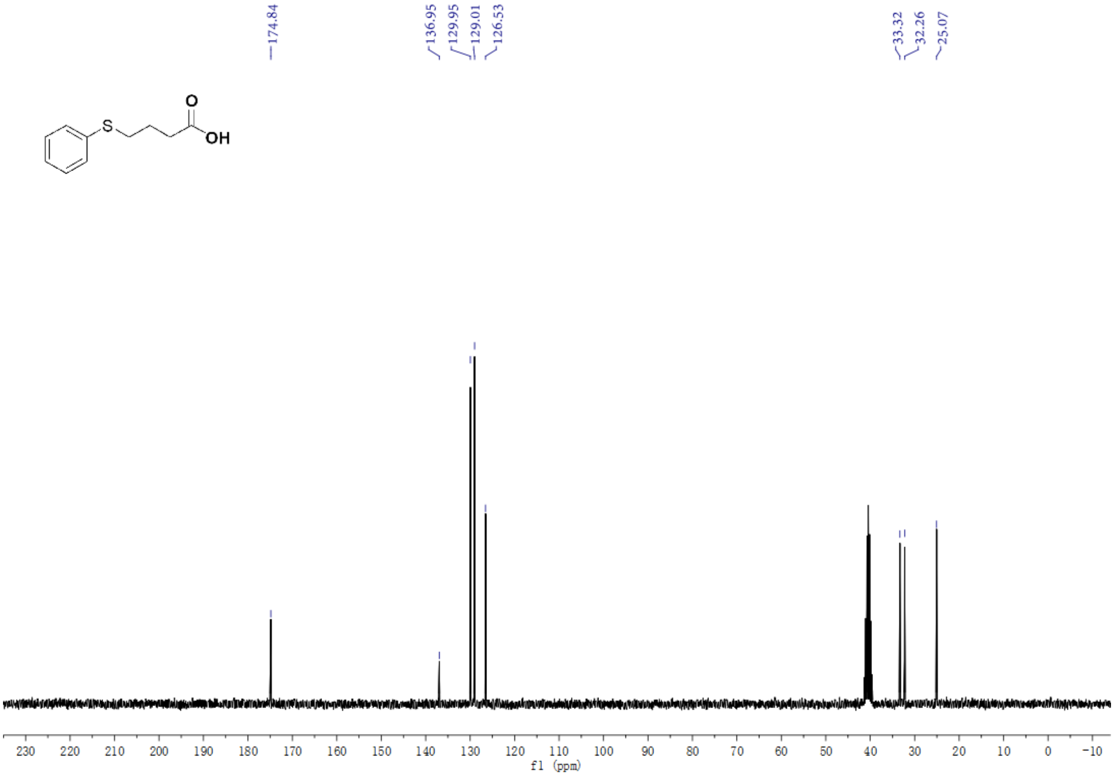

2h

^{1}H NMR (300 MHz, DMSO)

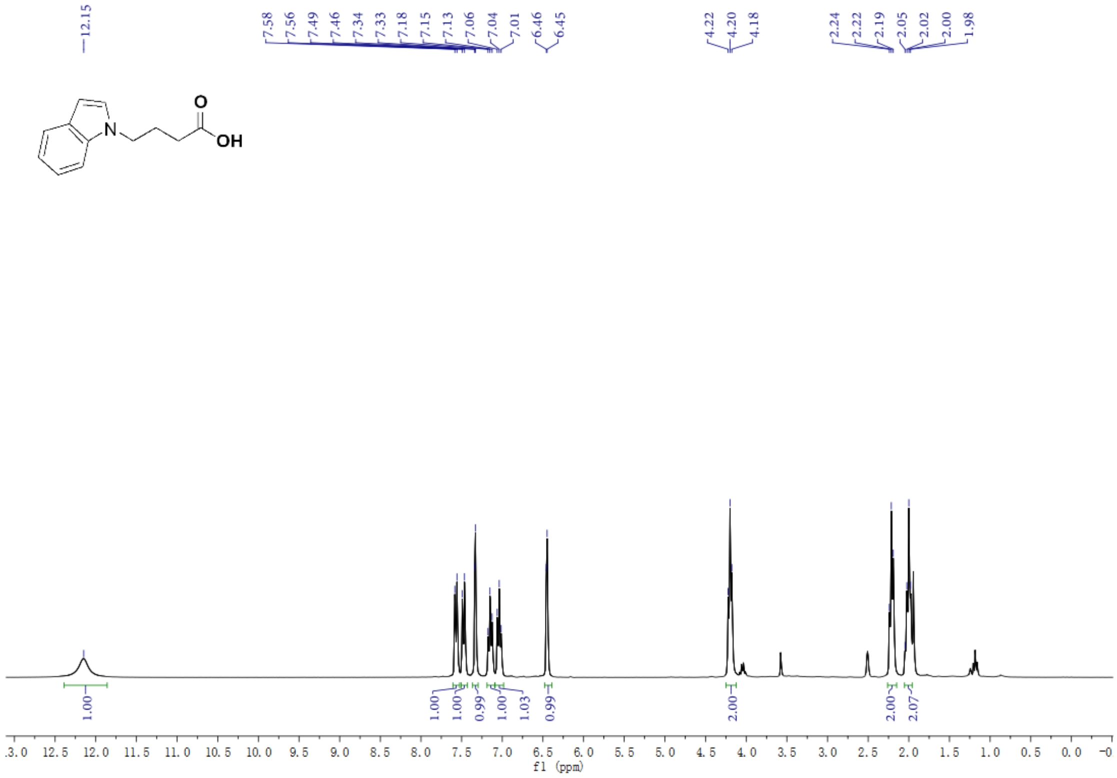

^{13}C NMR (75 MHz, DMSO)

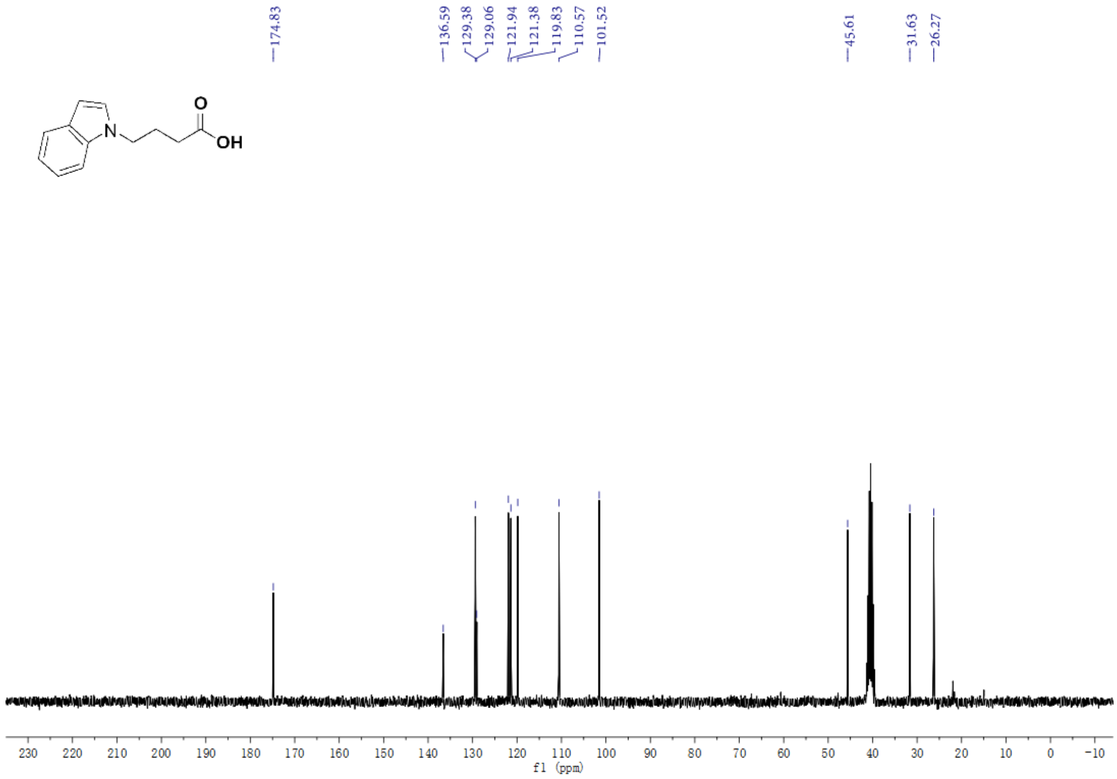

2i

^{1}H NMR (300 MHz, $CDCl_3$)

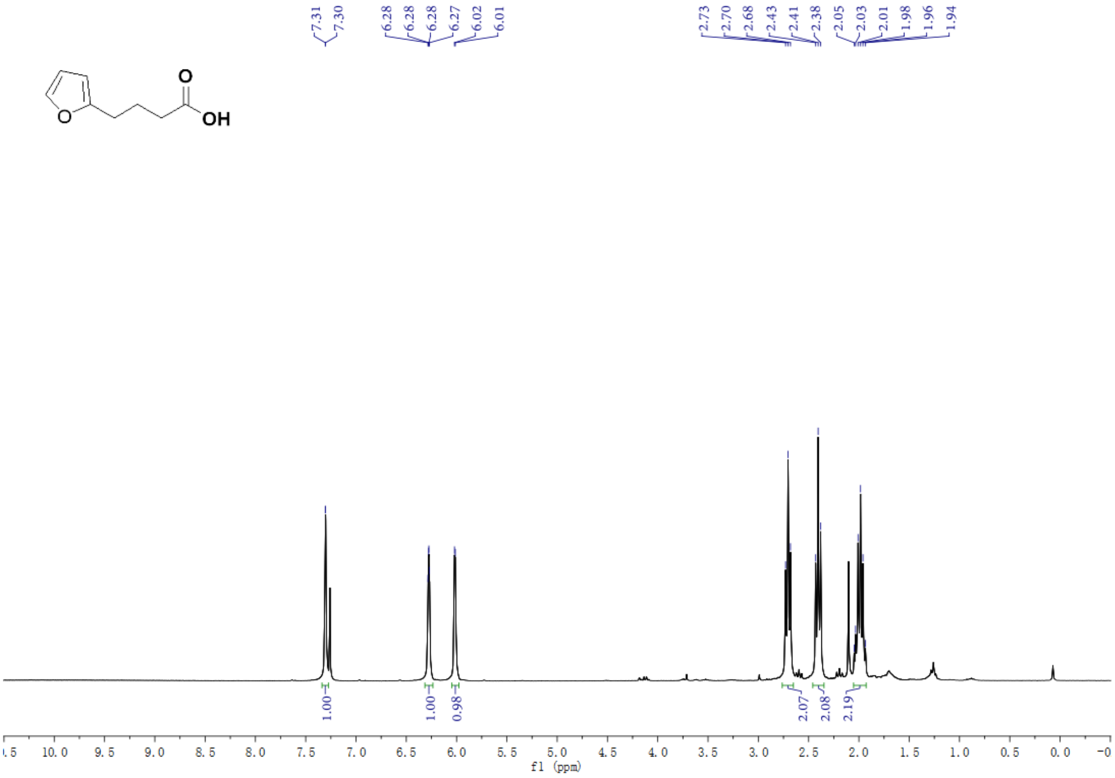

^{13}C NMR (75 MHz, $CDCl_3$)

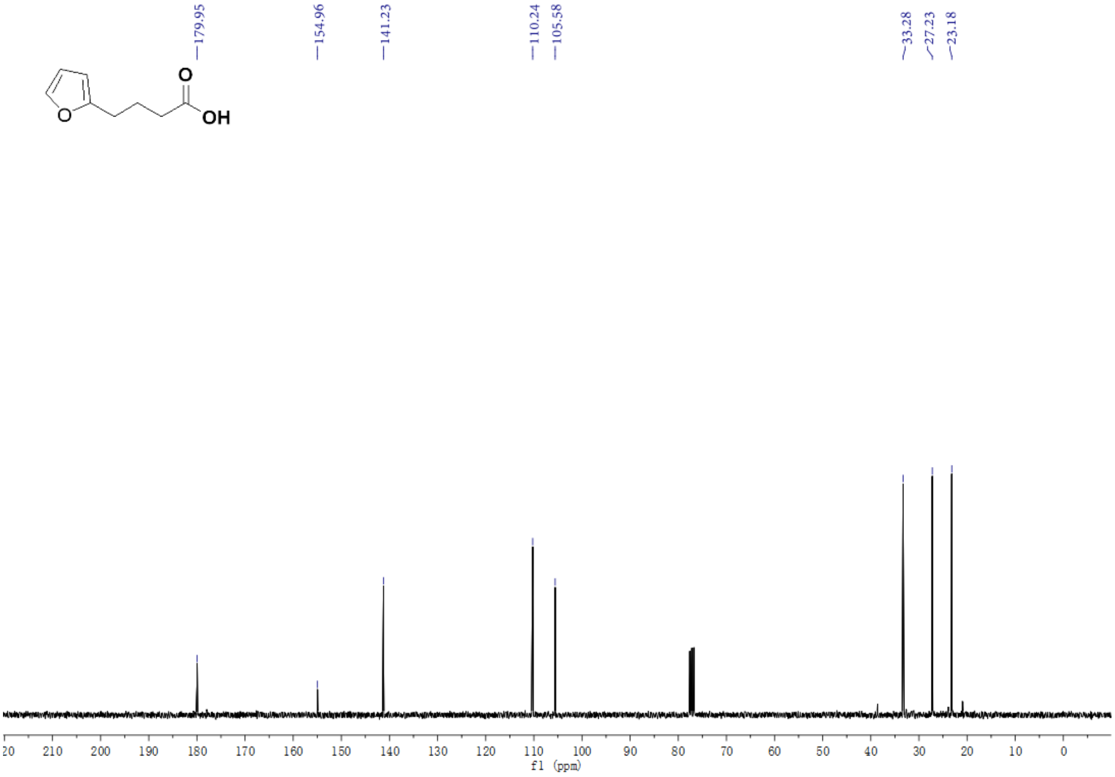

2j

^{1}H NMR (400 MHz, $CDCl_3$)

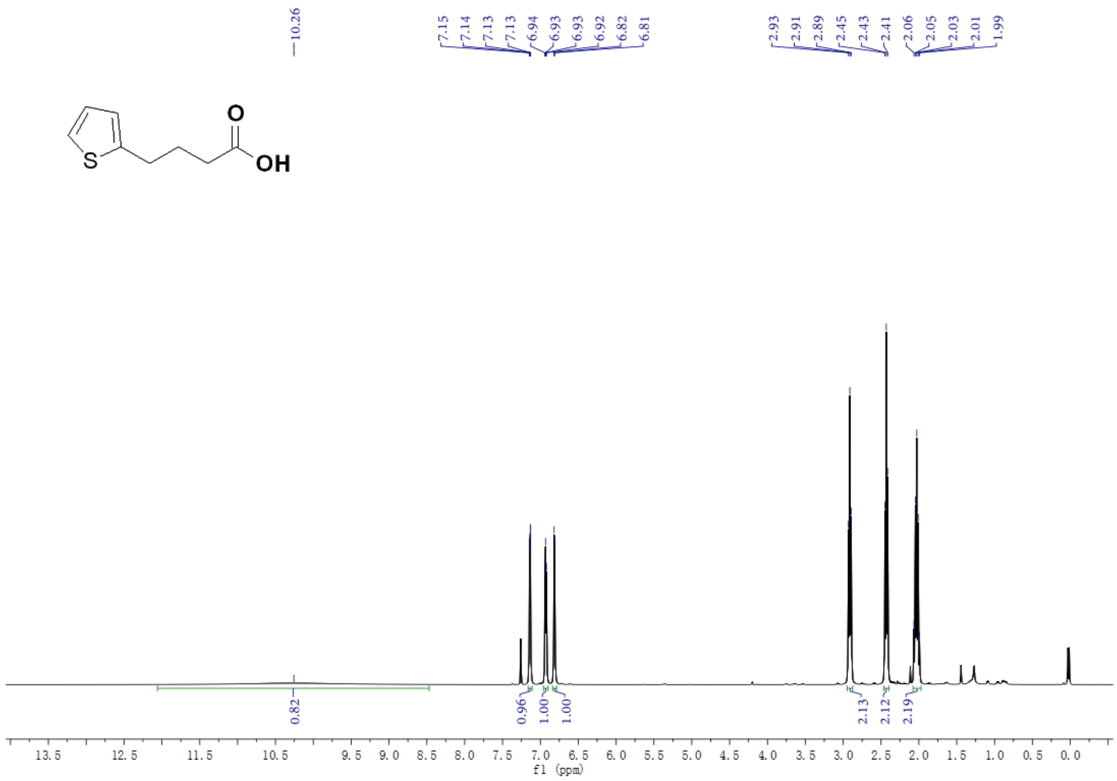

^{13}C NMR (101 MHz, $CDCl_3$)

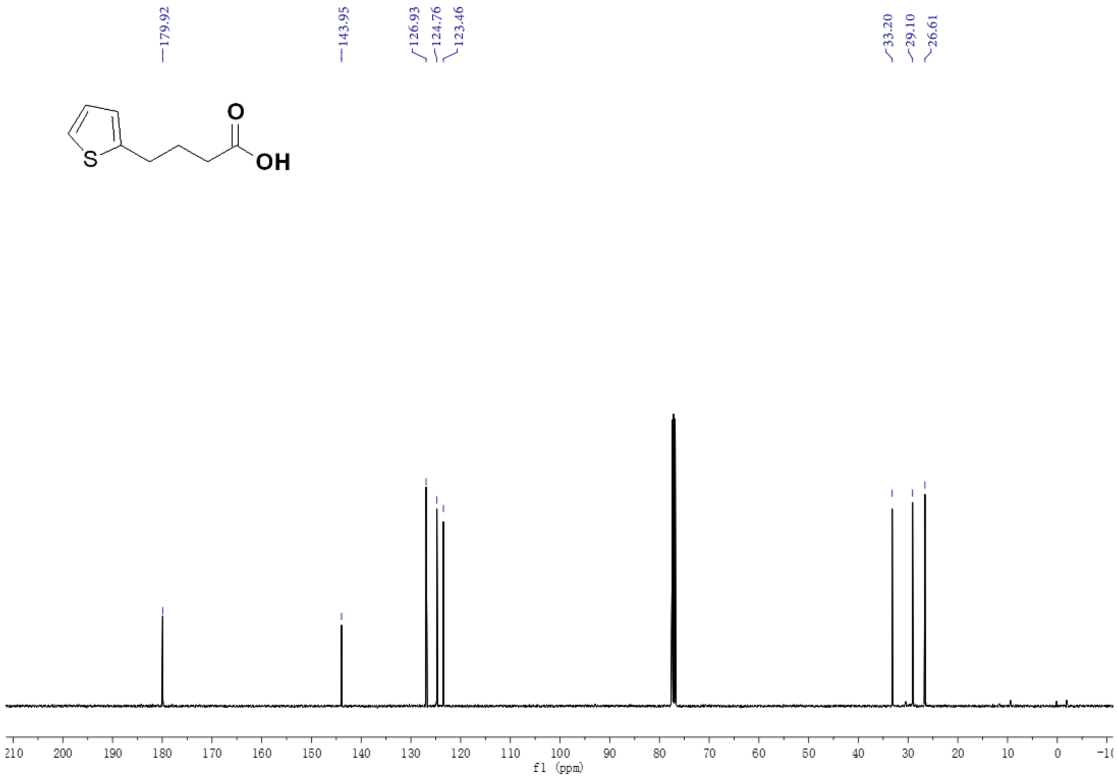

2k

^{1}H NMR (400 MHz, $CDCl_3$)

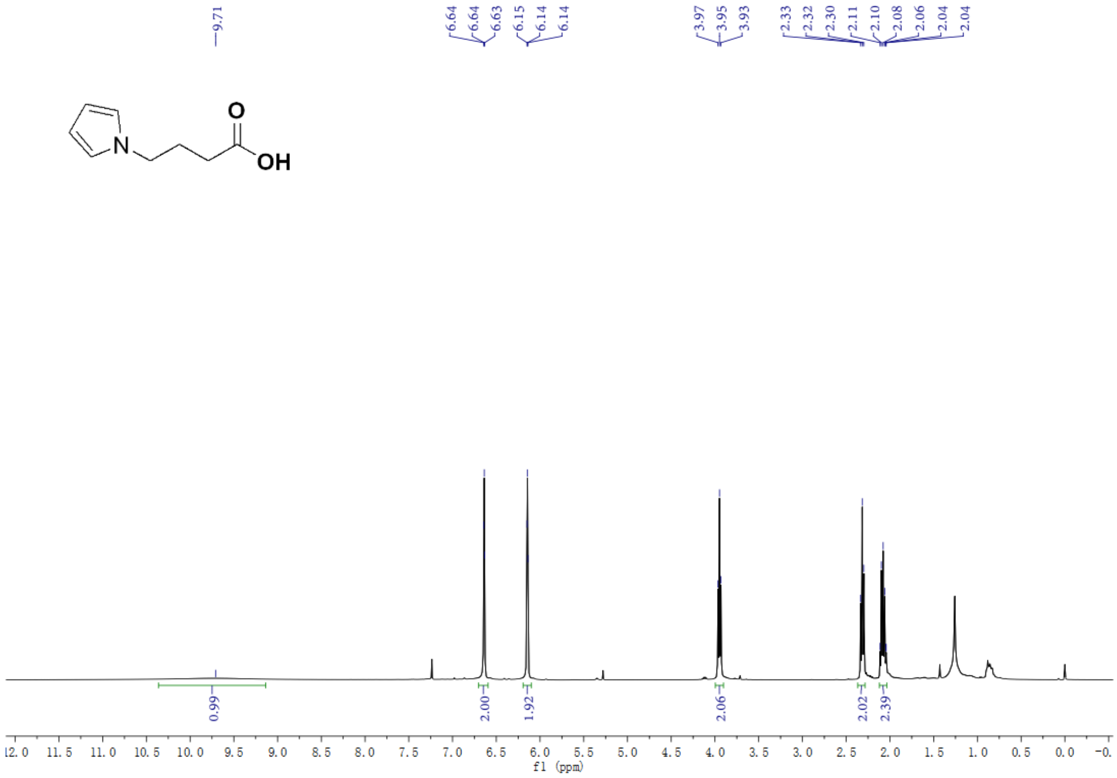

^{13}C NMR (101 MHz, $CDCl_3$)

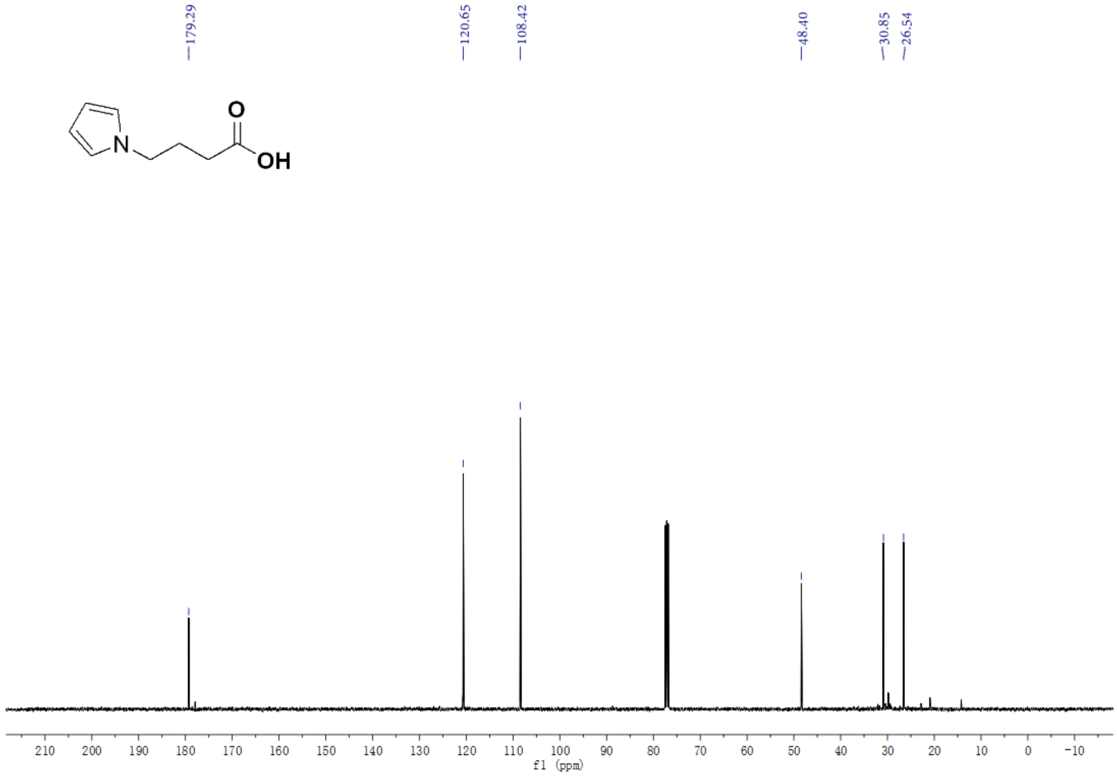

2l

^{1}H NMR (400 MHz, $CDCl_3$)

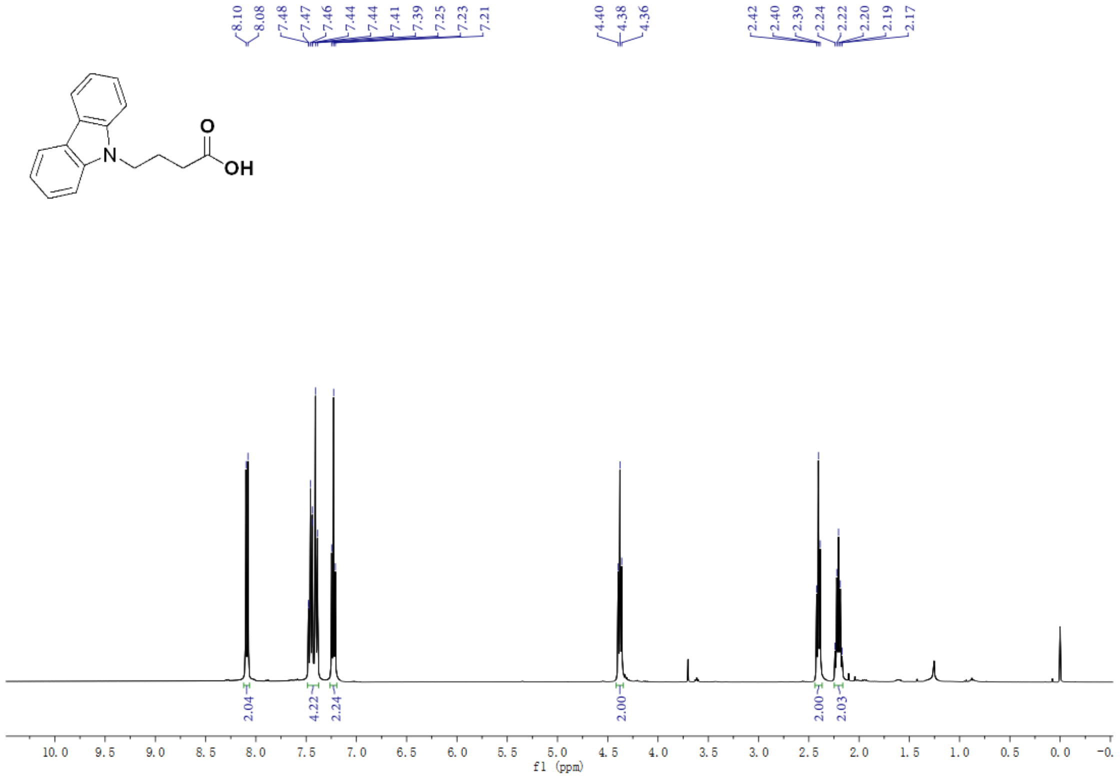

^{13}C NMR (101 MHz, $CDCl_3$)

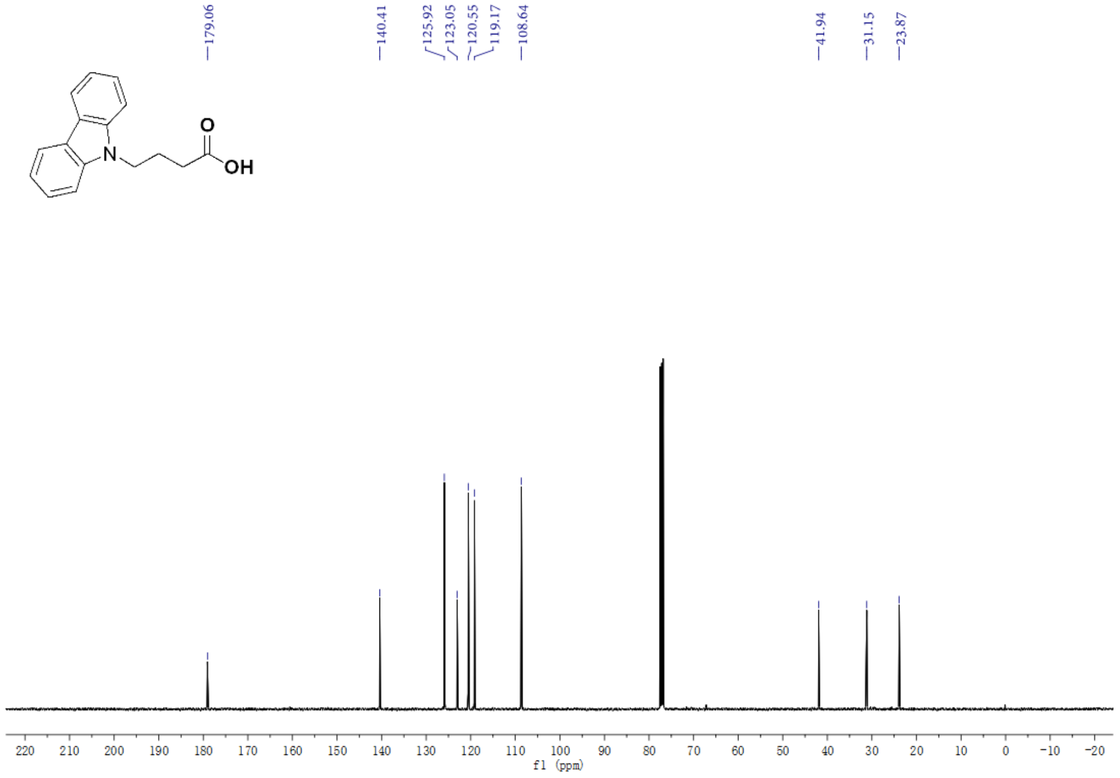

2m

^{1}H NMR (400 MHz, $CDCl_3$)

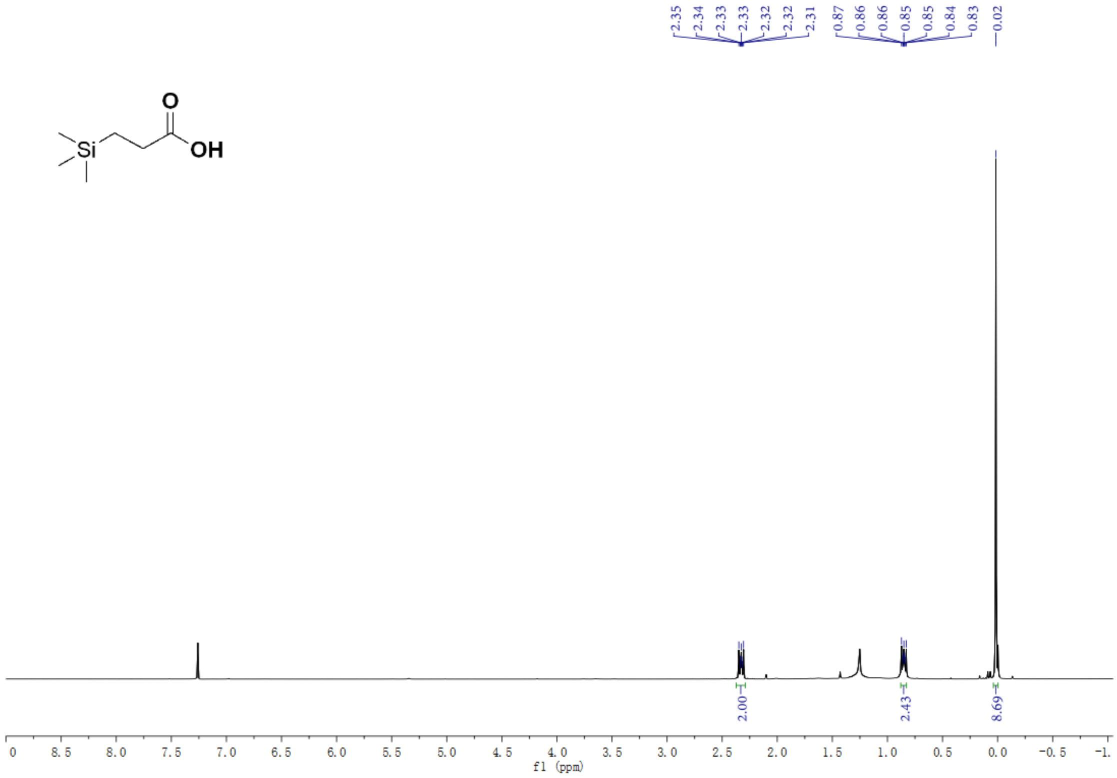

^{13}C NMR (101 MHz, $CDCl_3$)

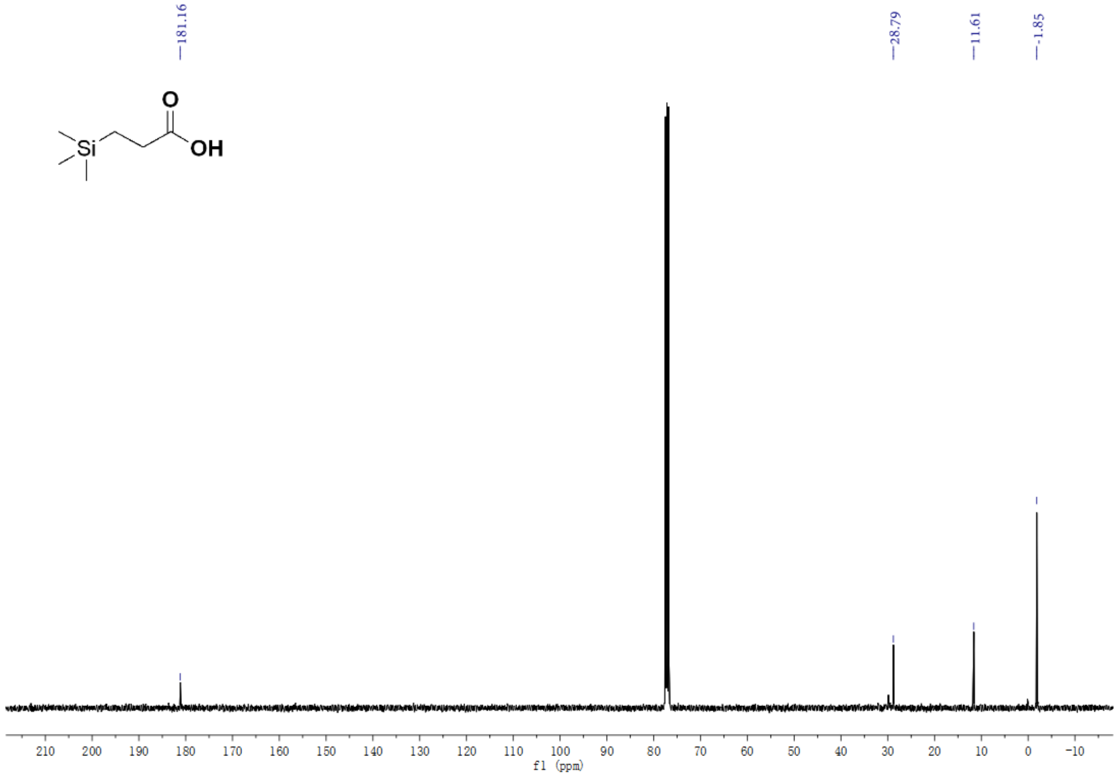

2n

¹H NMR (400 MHz, $CDCl_3$)

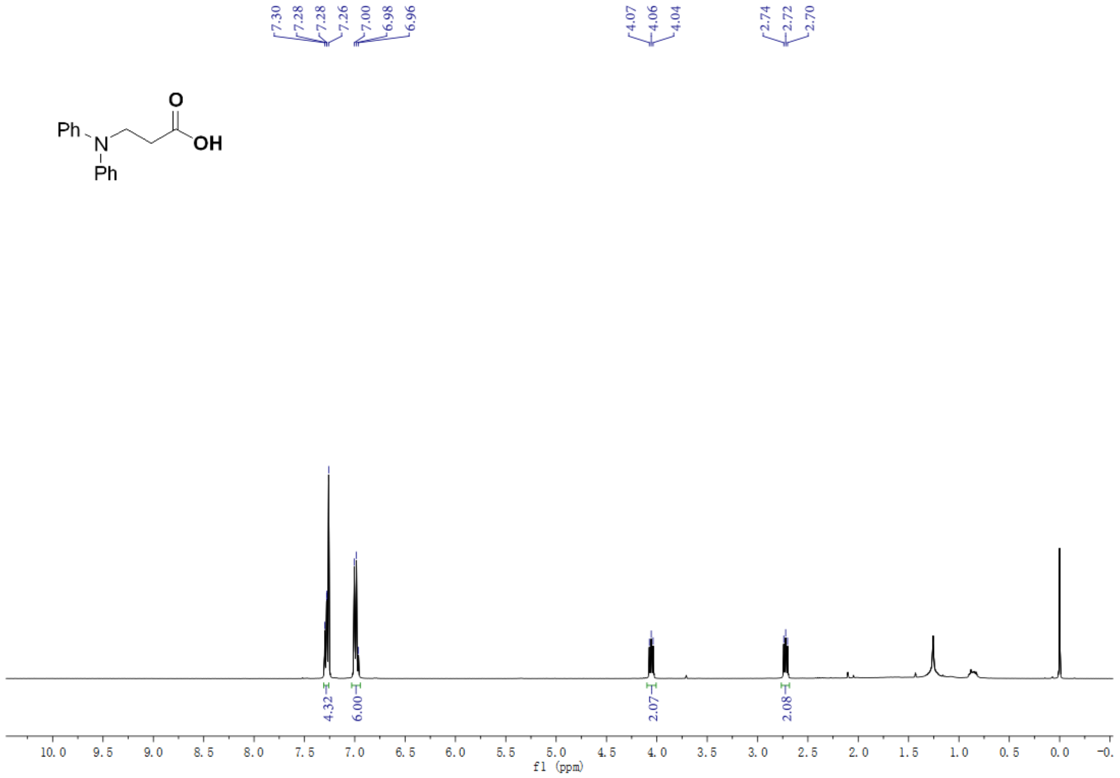

¹³C NMR (101 MHz, $CDCl_3$)

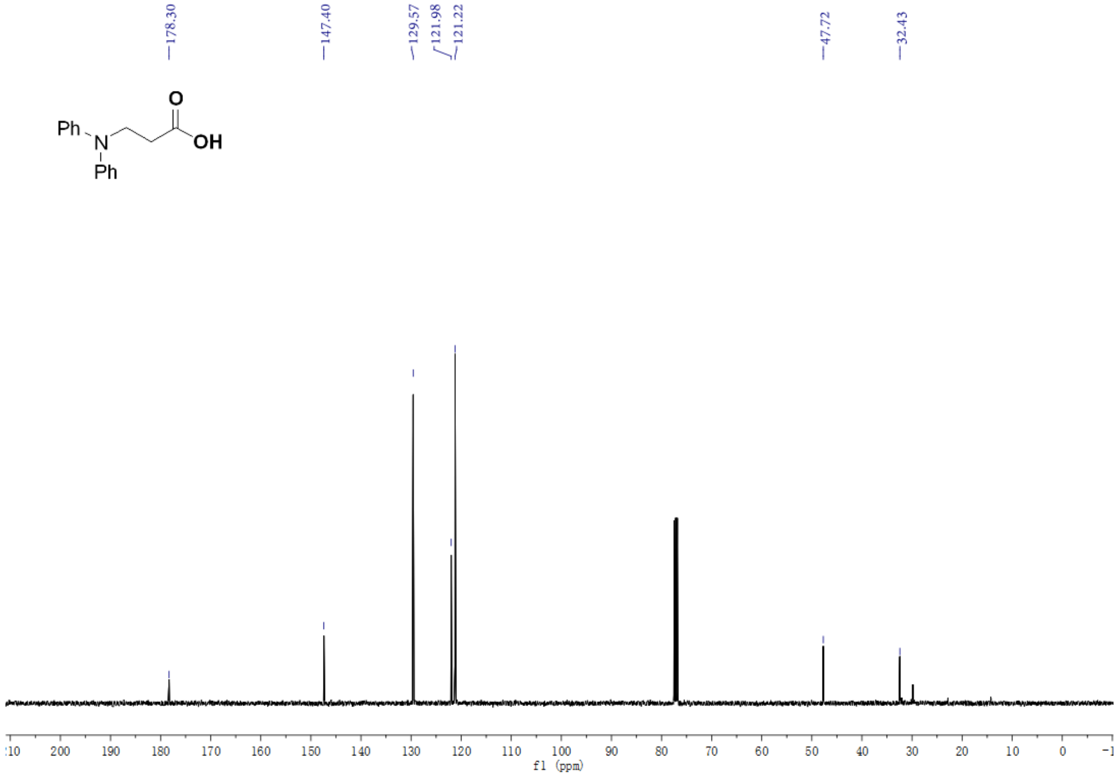

2o

^{1}**H NMR** (400 MHz, DMSO)

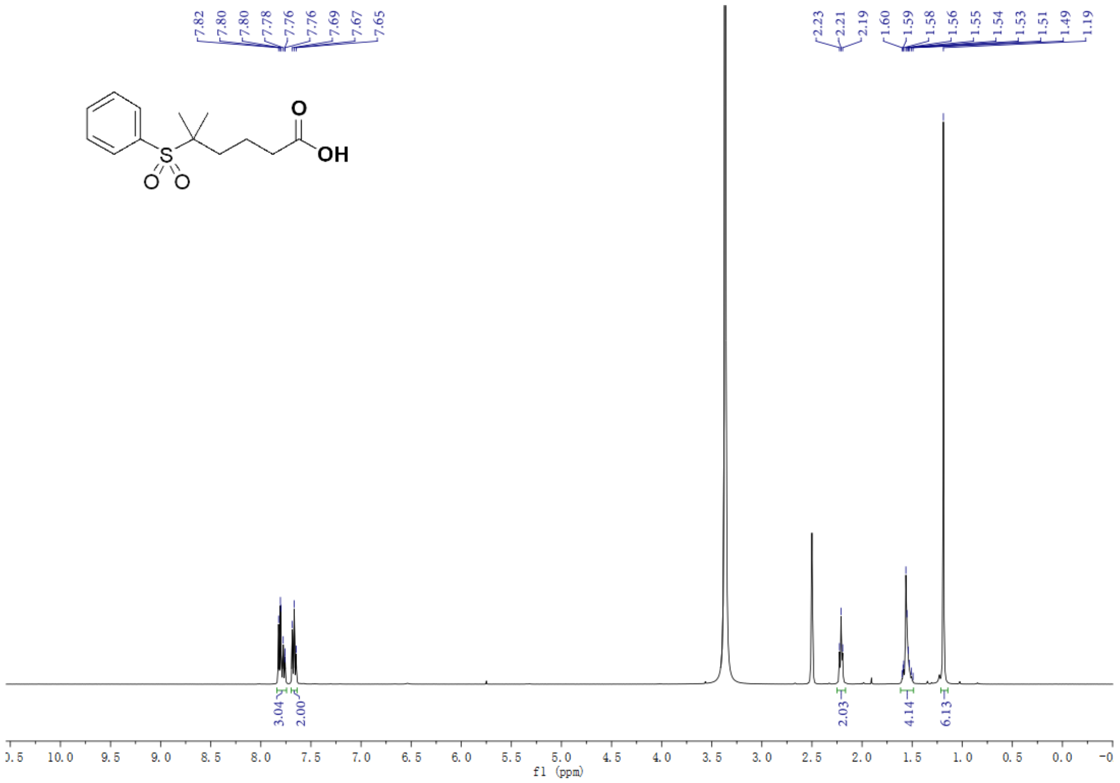

^{13}**C NMR** (101 MHz, DMSO)

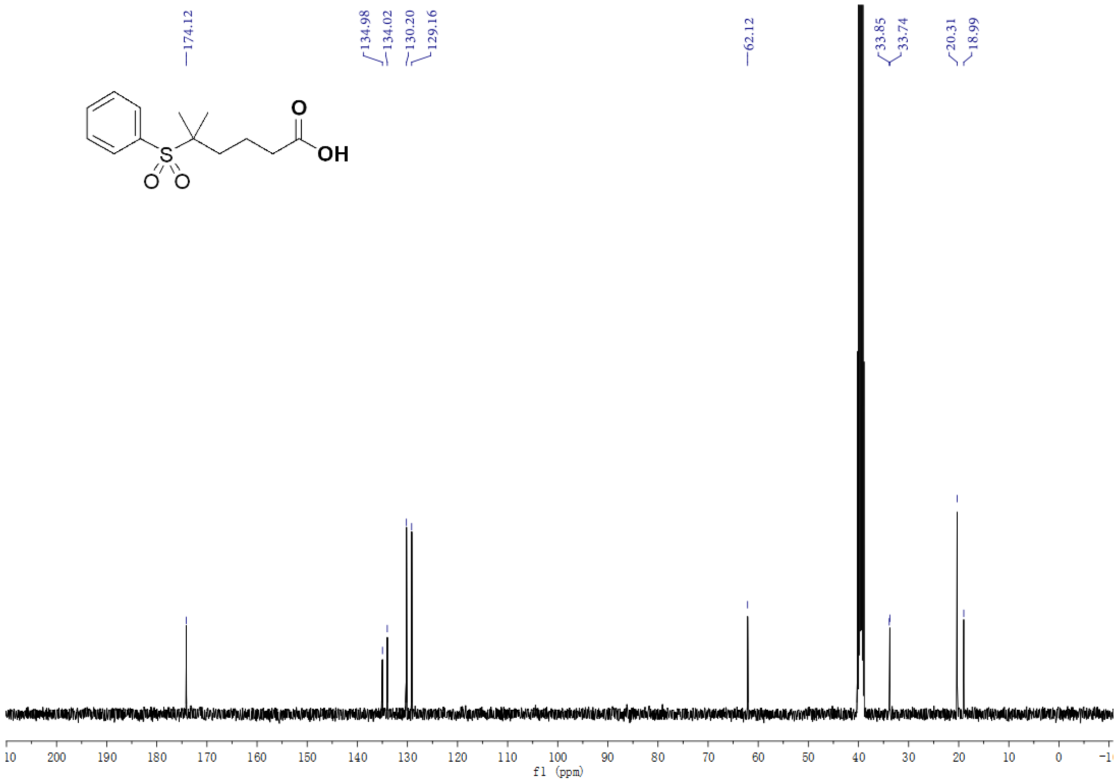

2p

^{1}H NMR (400 MHz, $CDCl_3$)

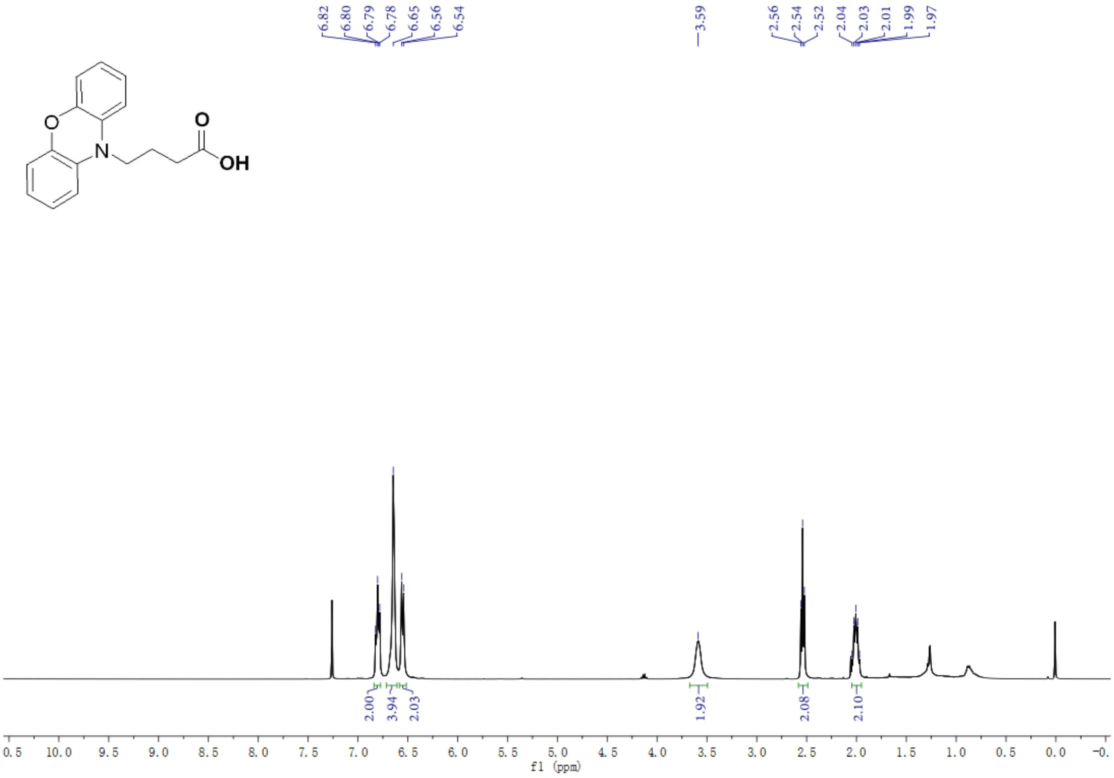

^{13}C NMR (101 MHz, $CDCl_3$)

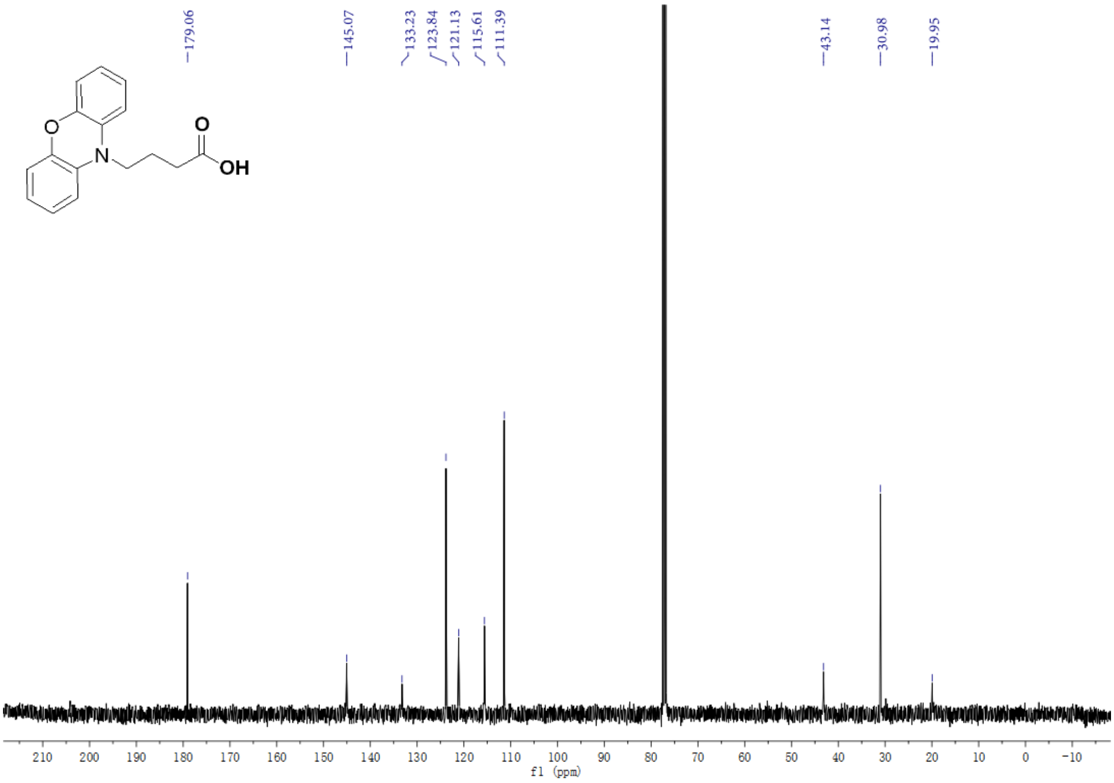

2q

^{1}H NMR (400 MHz, $CDCl_3$)

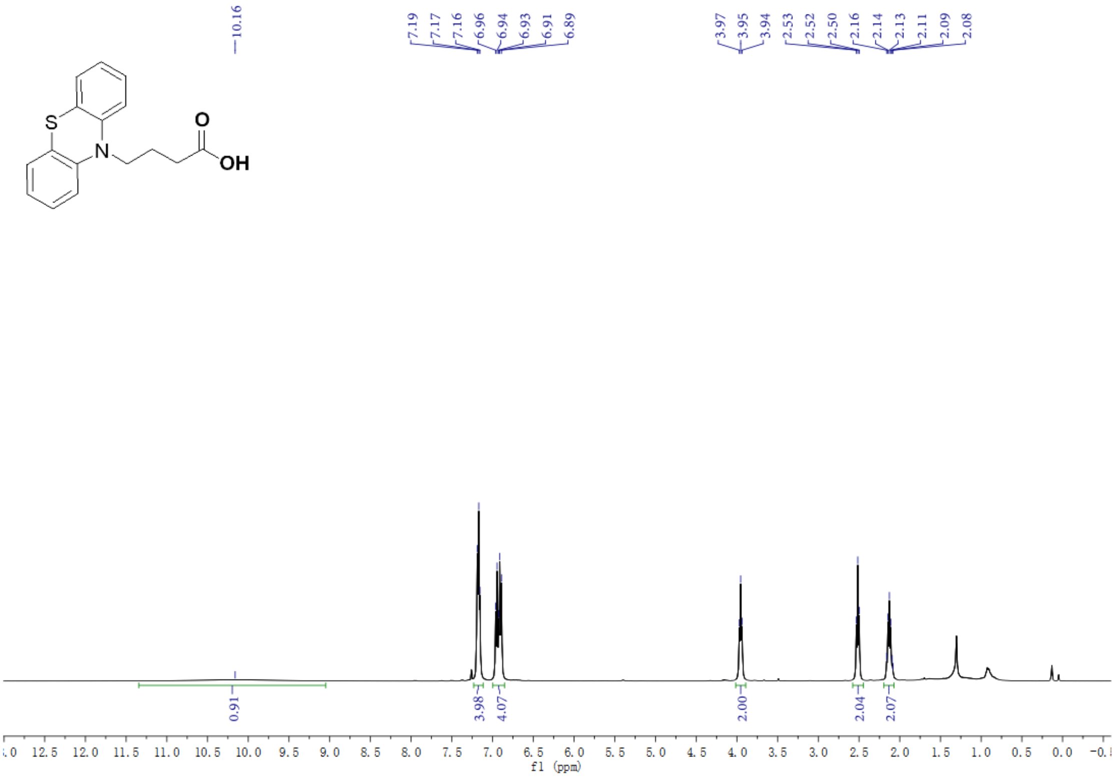

^{13}C NMR (101 MHz, $CDCl_3$)

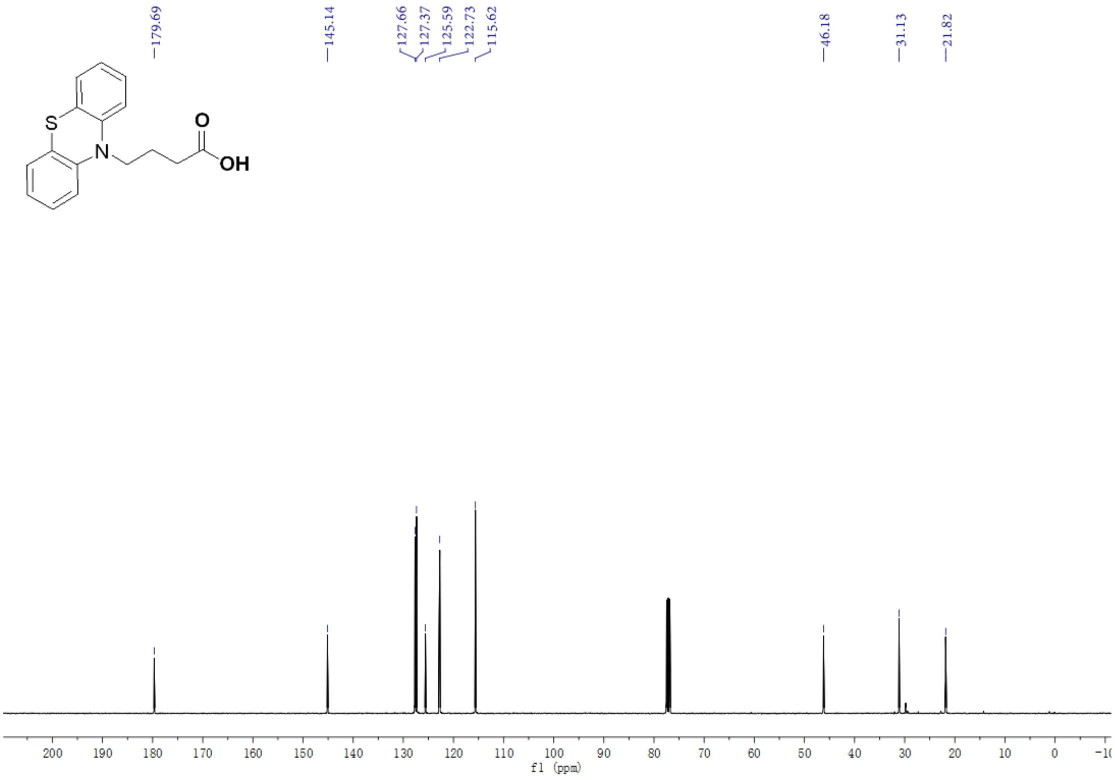

2r

^{1}H NMR (400 MHz, $CDCl_3$)

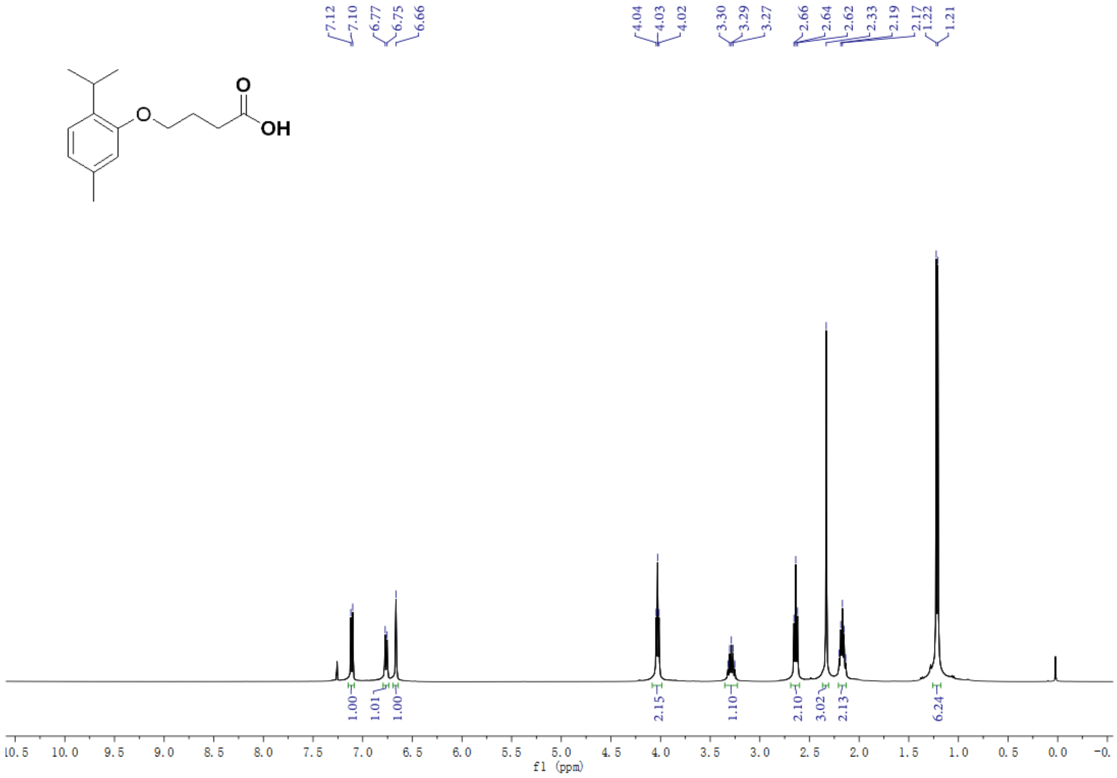

^{13}C NMR (101 MHz, $CDCl_3$)

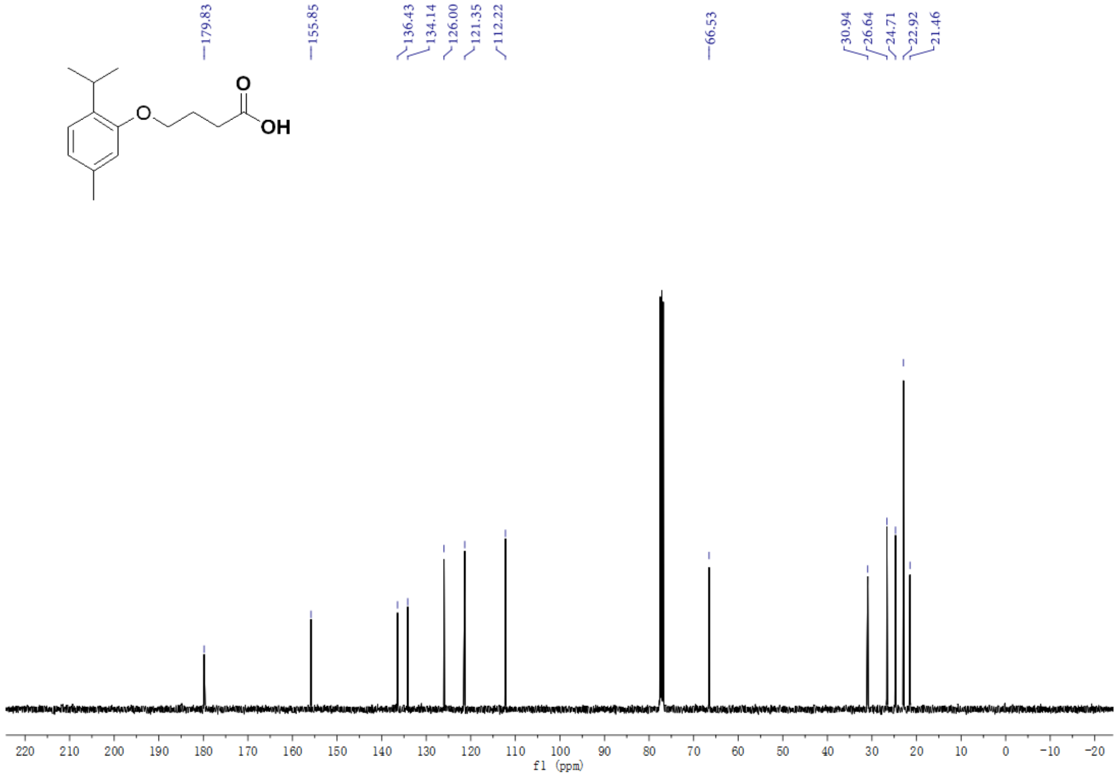

2s

^{1}H NMR (300 MHz, $CDCl_3$)

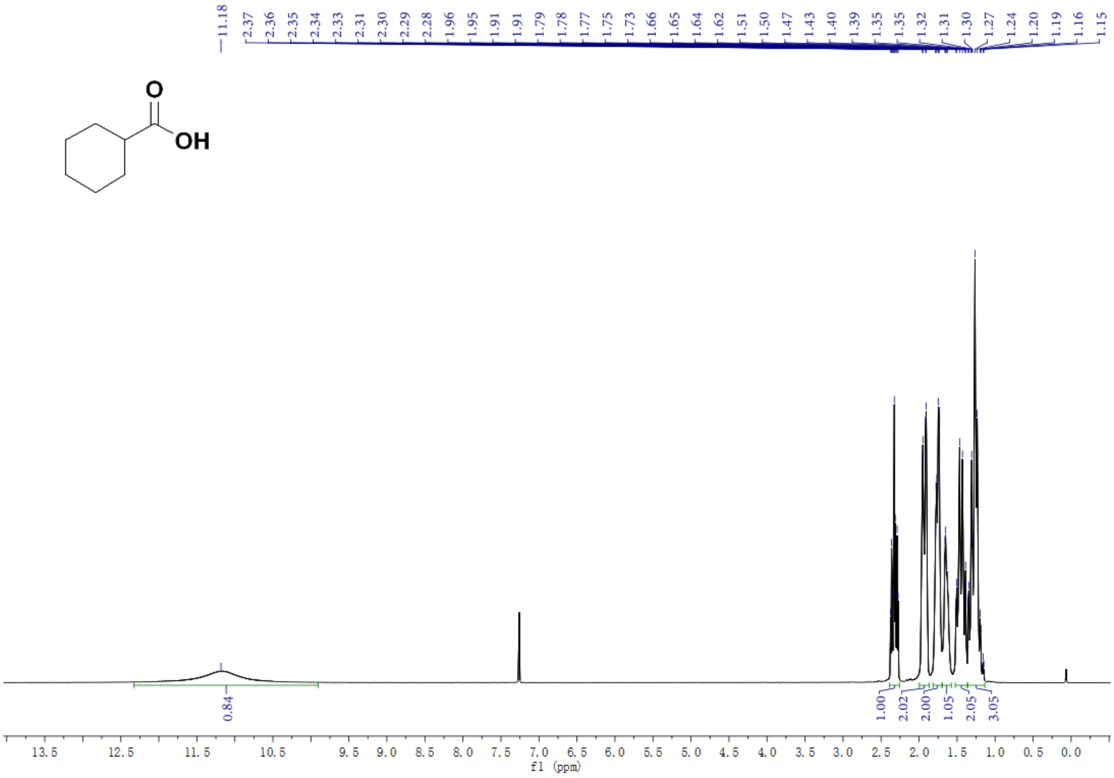

^{13}C NMR (75 MHz, $CDCl_3$)

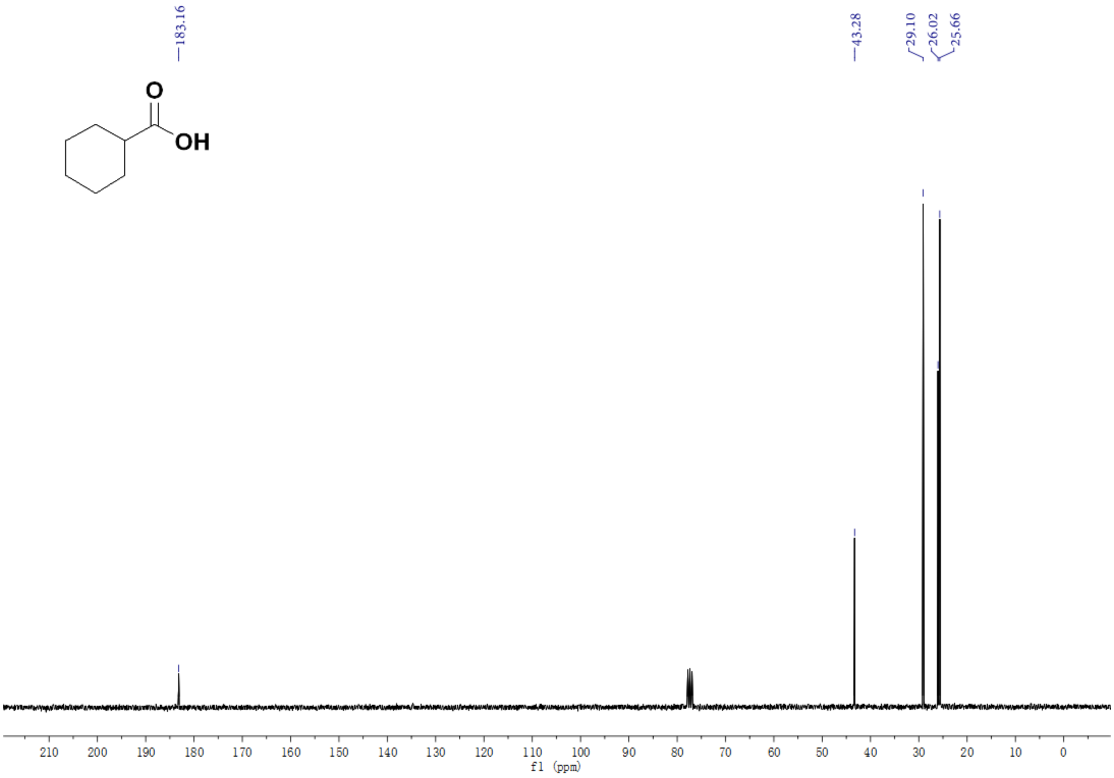

2t

¹H NMR (400 MHz, DMSO)

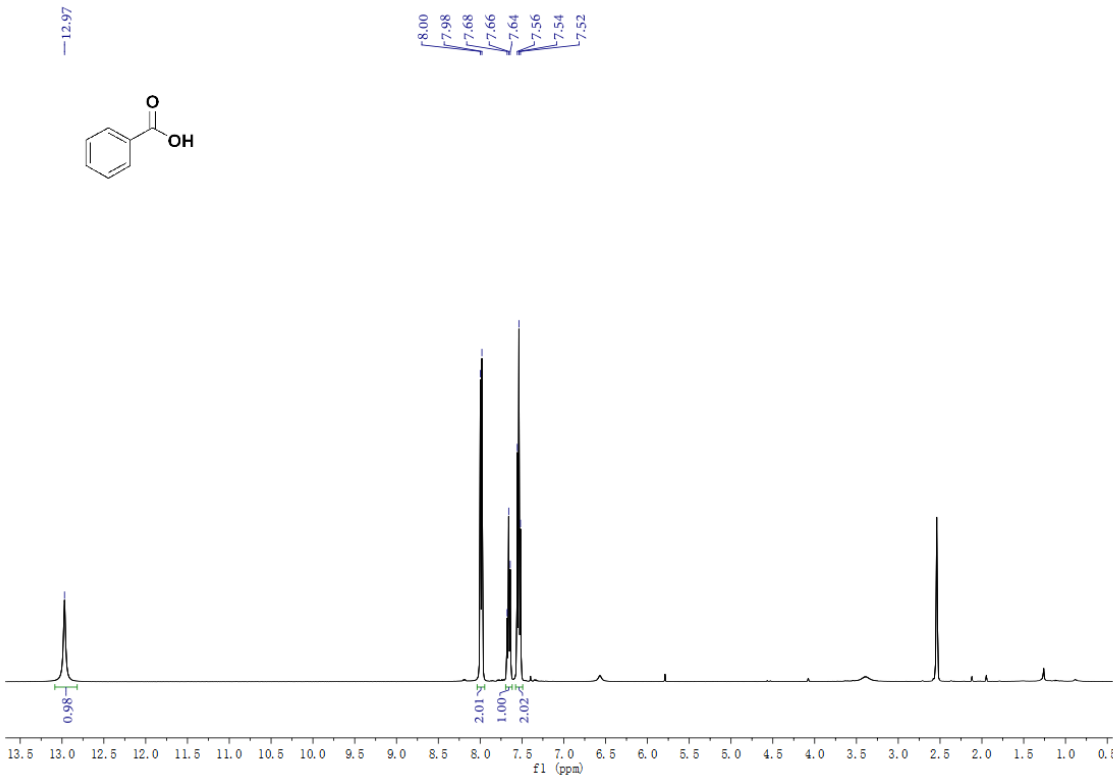

¹³C NMR (101 MHz, DMSO)

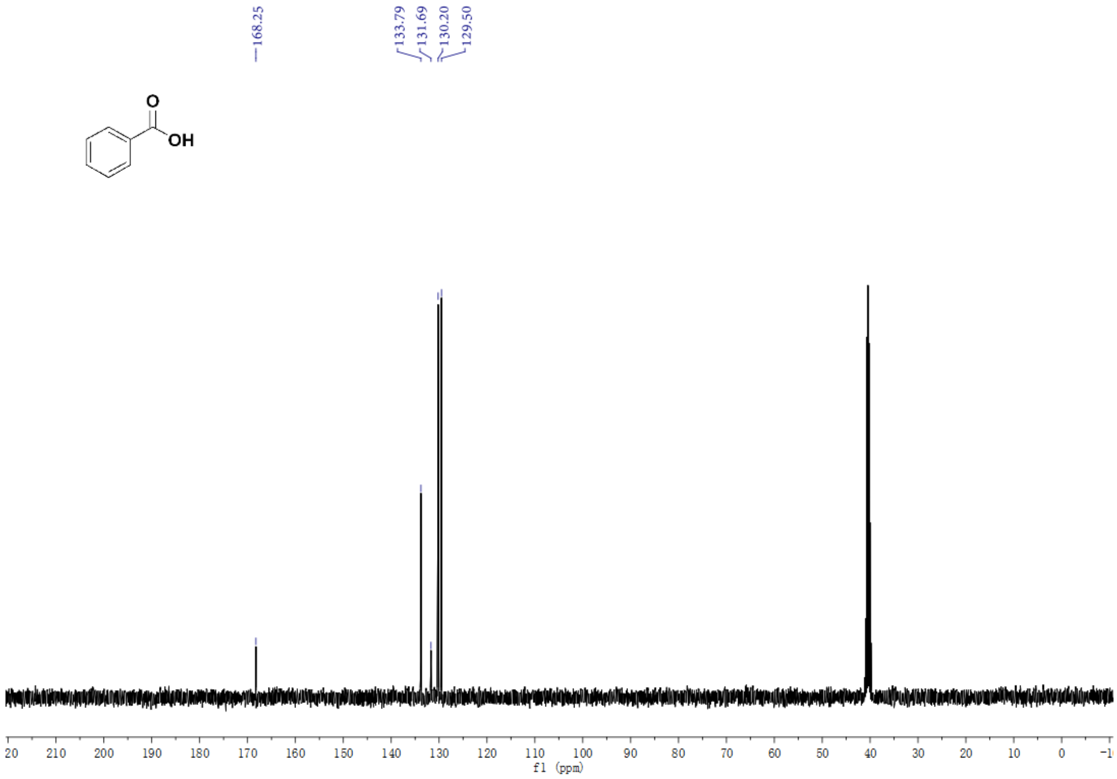

2u

^{1}H NMR (400 MHz, DMSO)

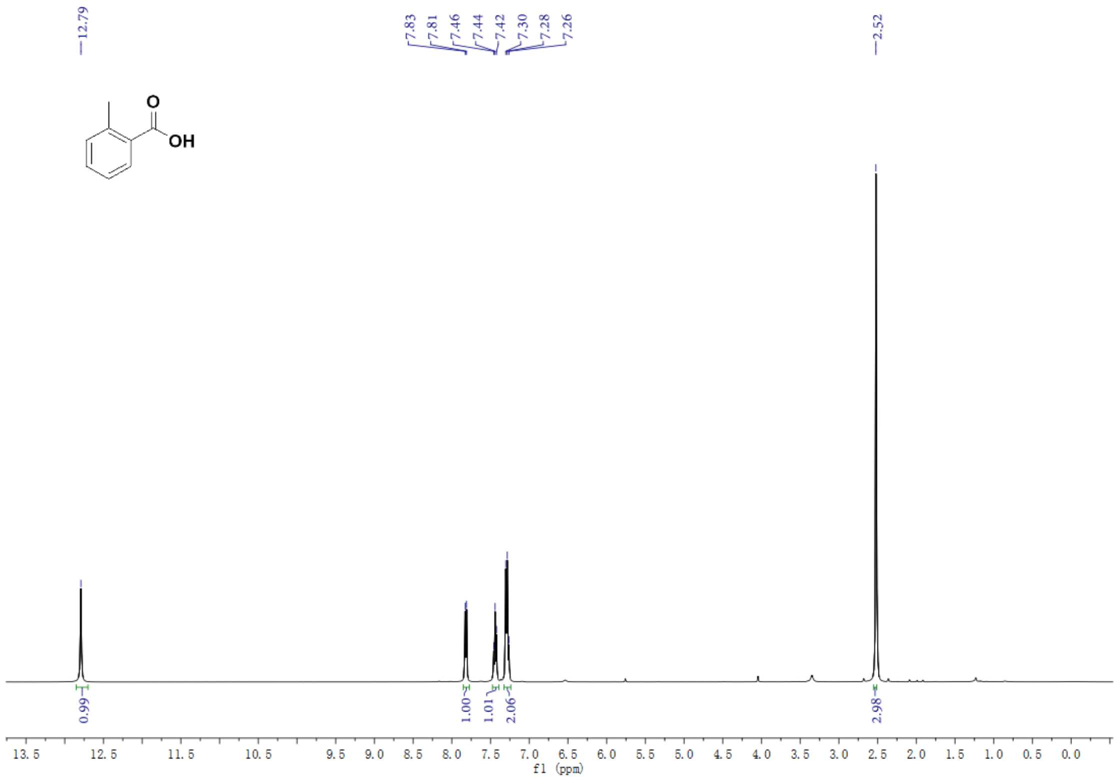

^{13}C NMR (101 MHz, DMSO)

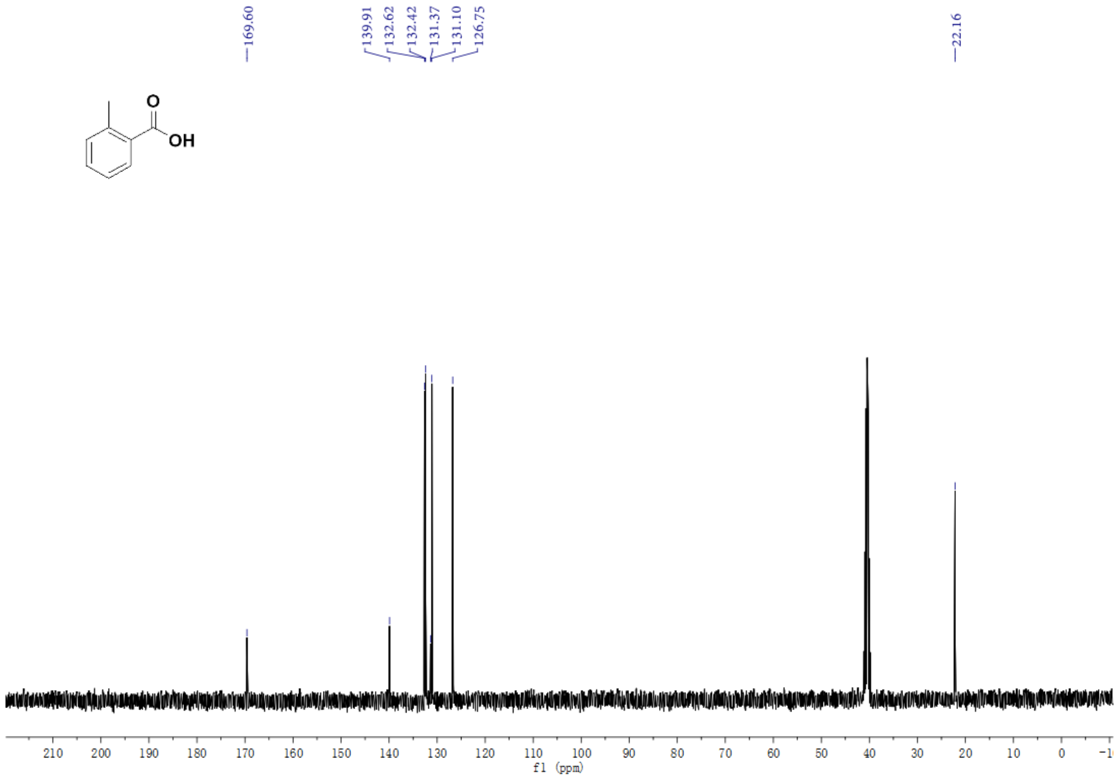

2v

^{1}H NMR (300 MHz, DMSO)

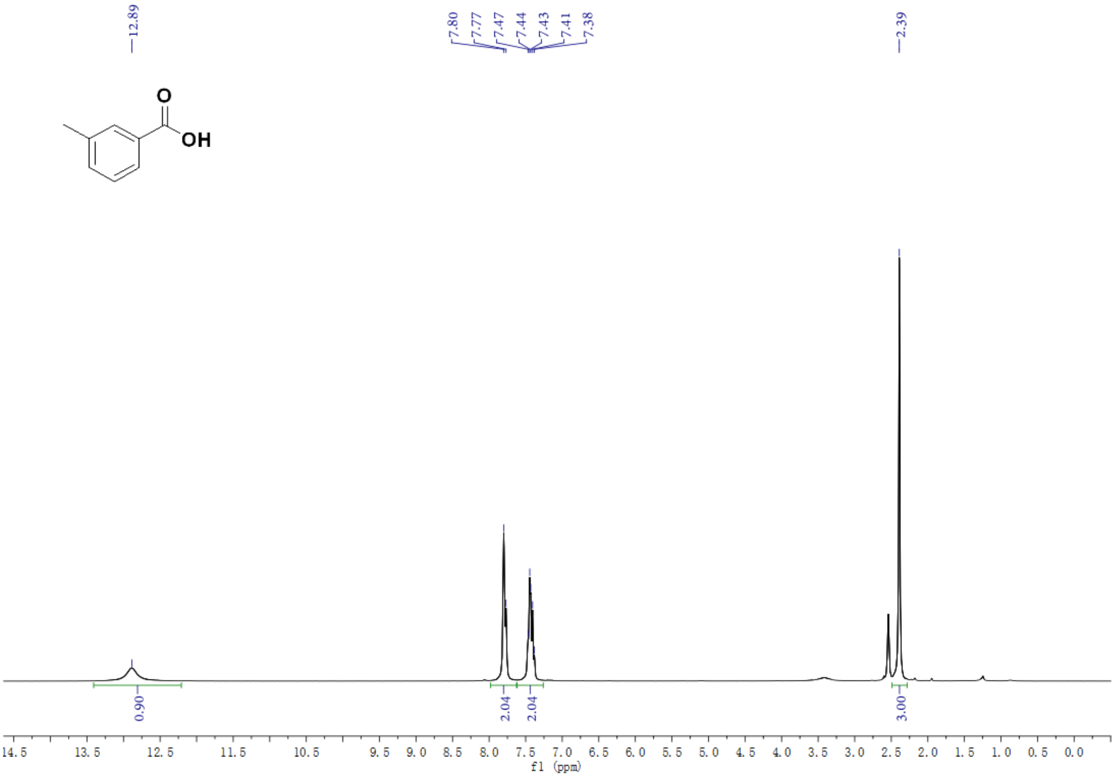

^{13}C NMR (75 MHz, DMSO)

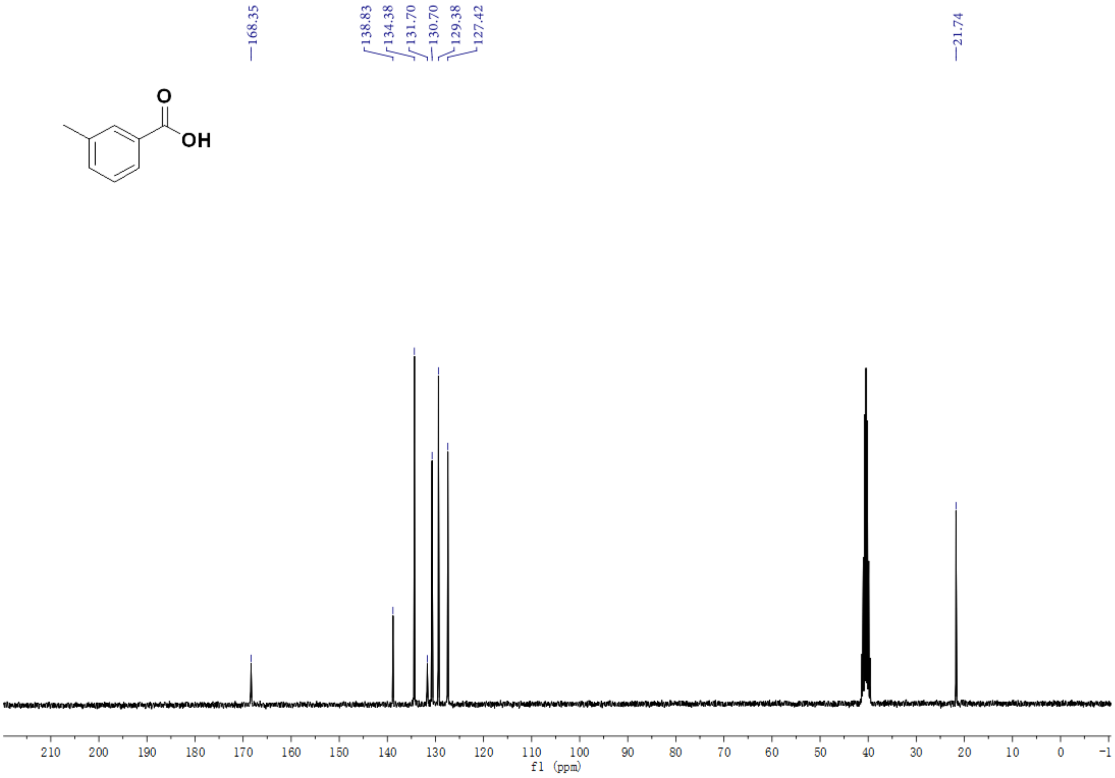

2w

^{1}H NMR (300 MHz, DMSO)

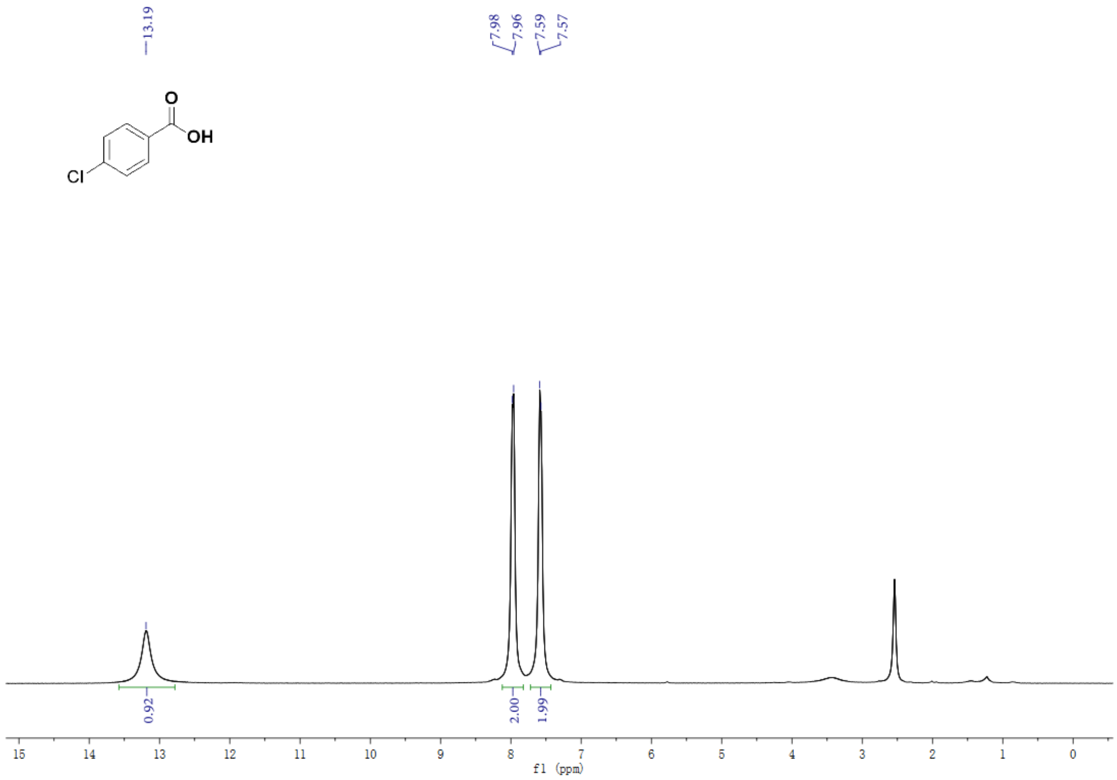

^{13}C NMR (75 MHz, DMSO)

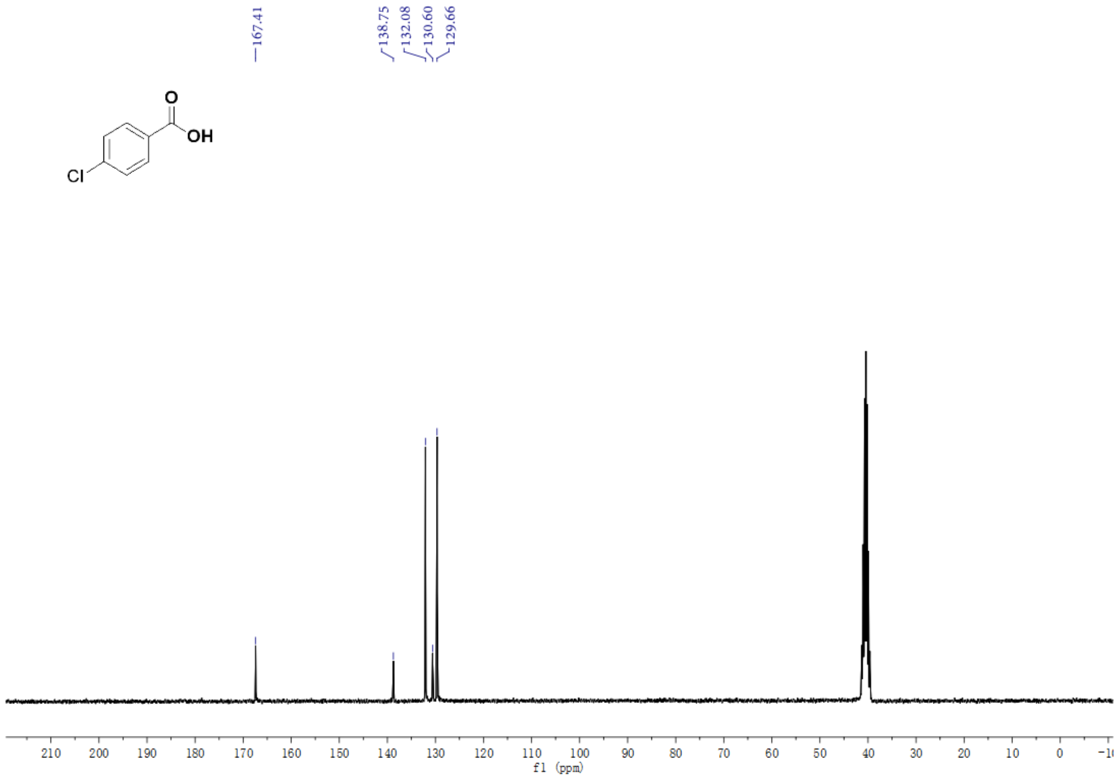

2x

^{1}H NMR (300 MHz, DMSO)

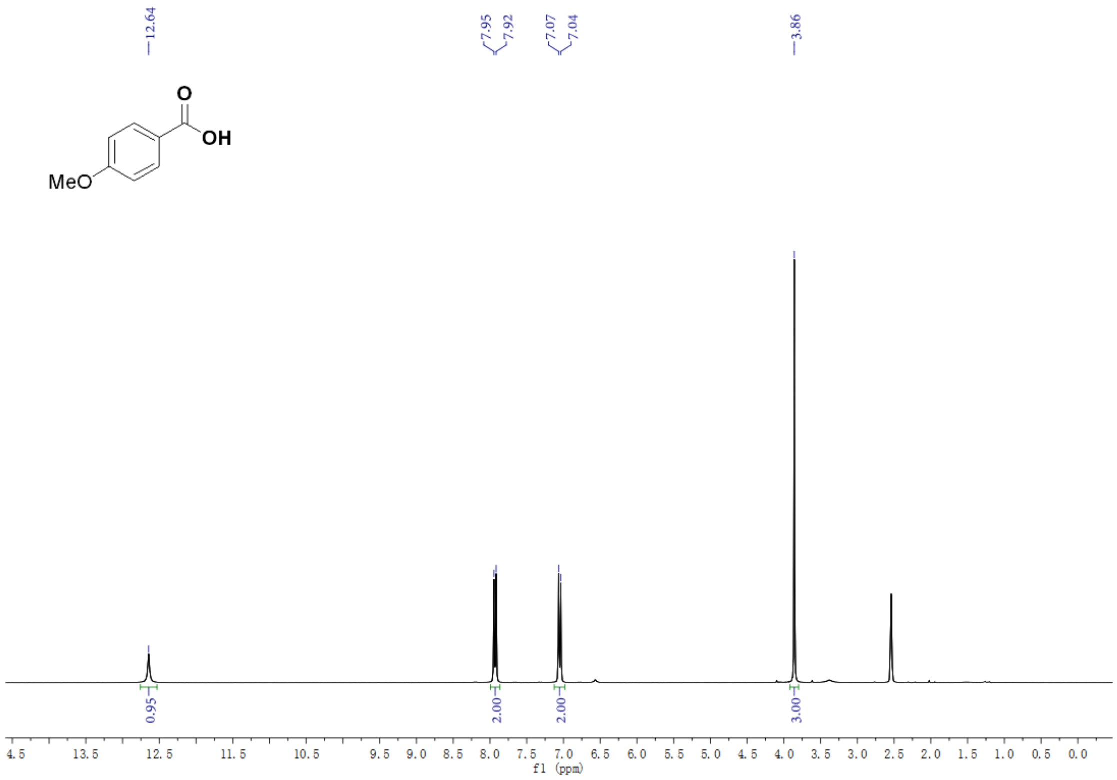

^{13}C NMR (75 MHz, DMSO)

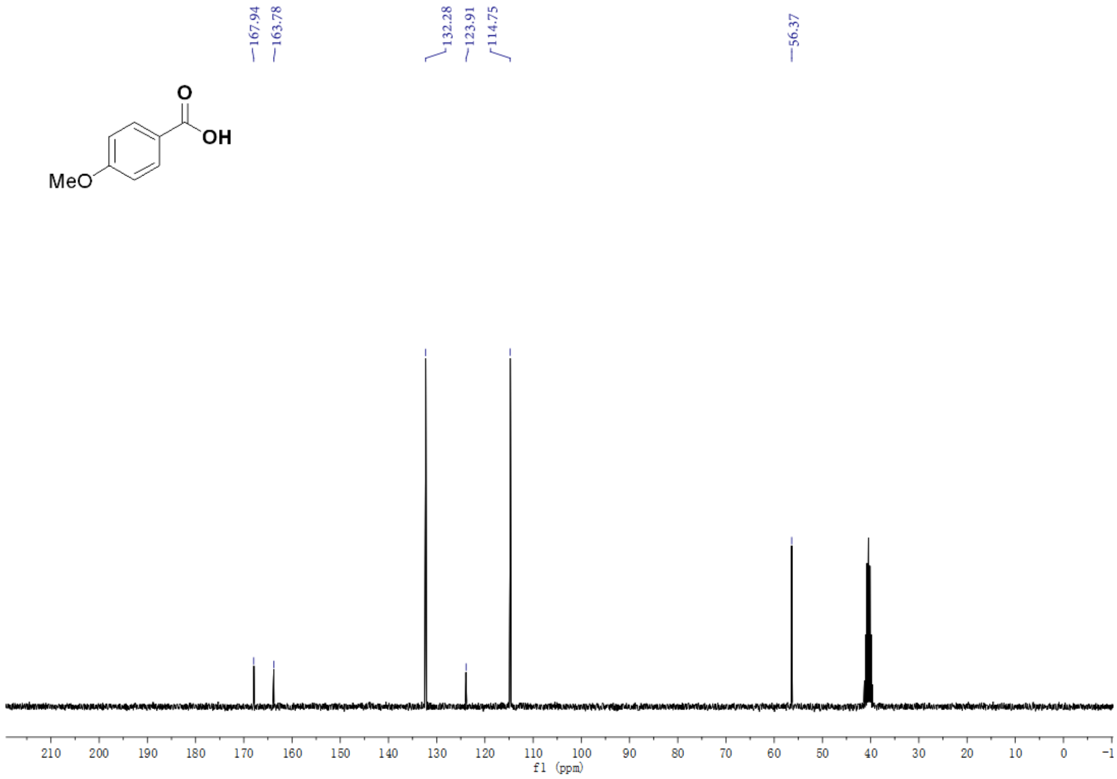

2y

^{1}H NMR (300 MHz, DMSO)

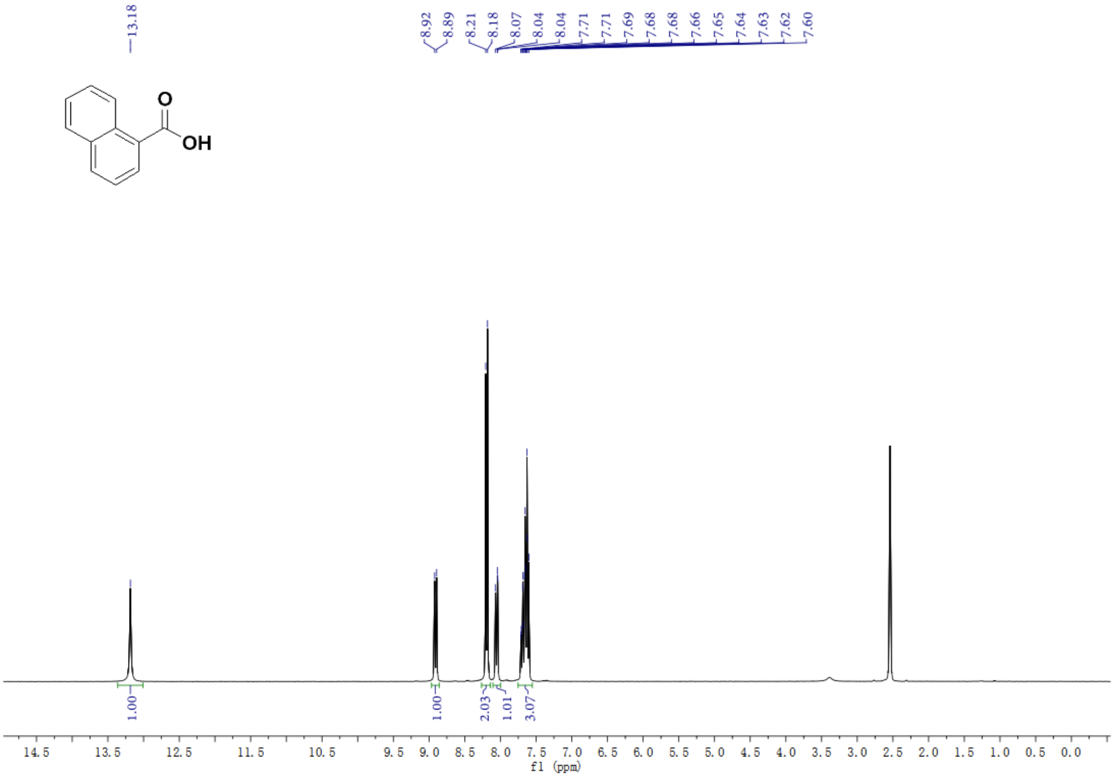

^{13}C NMR (75 MHz, DMSO)

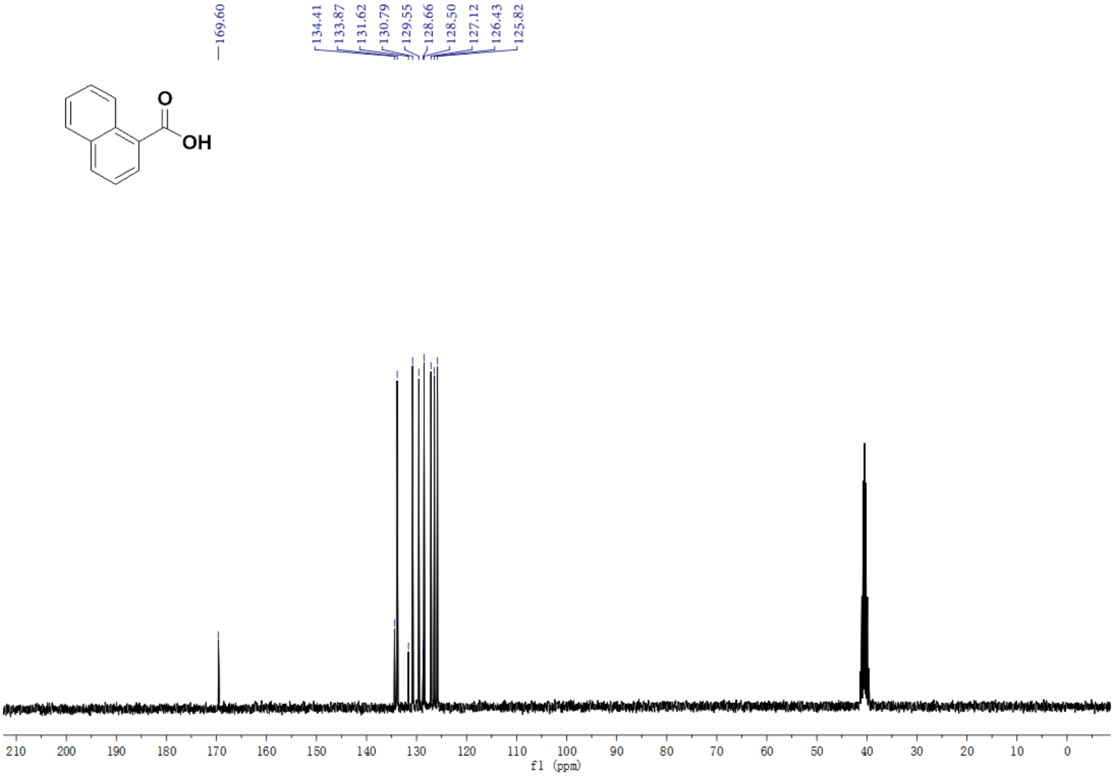

2z

^{1}H NMR (300 MHz, $CDCl_3$)

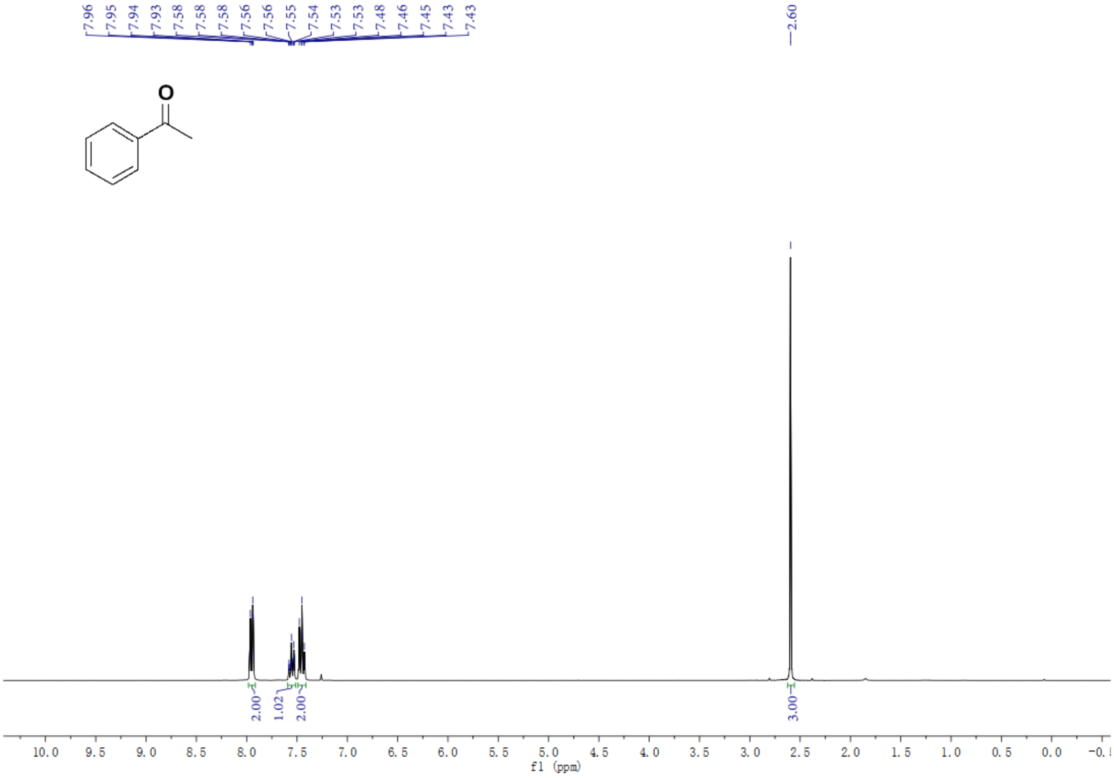

^{13}C NMR (75 MHz, $CDCl_3$)

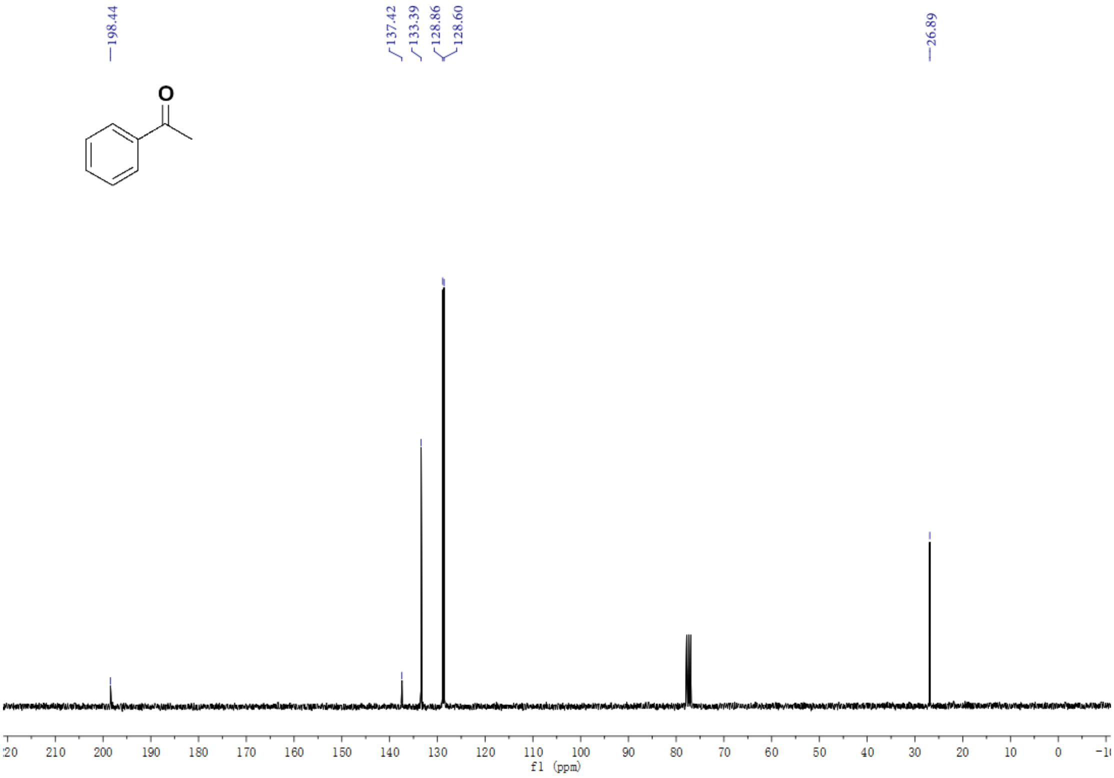

2aa

^{1}H NMR (300 MHz, $CDCl_3$)

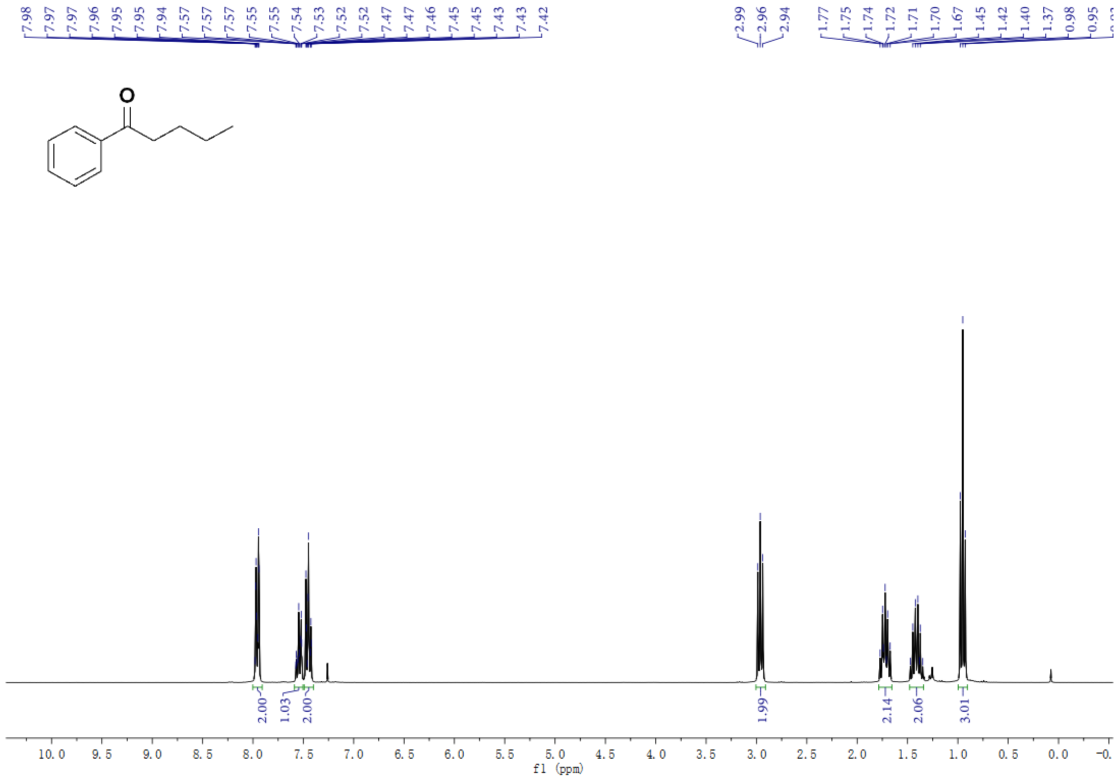

^{13}C NMR (75 MHz, $CDCl_3$)

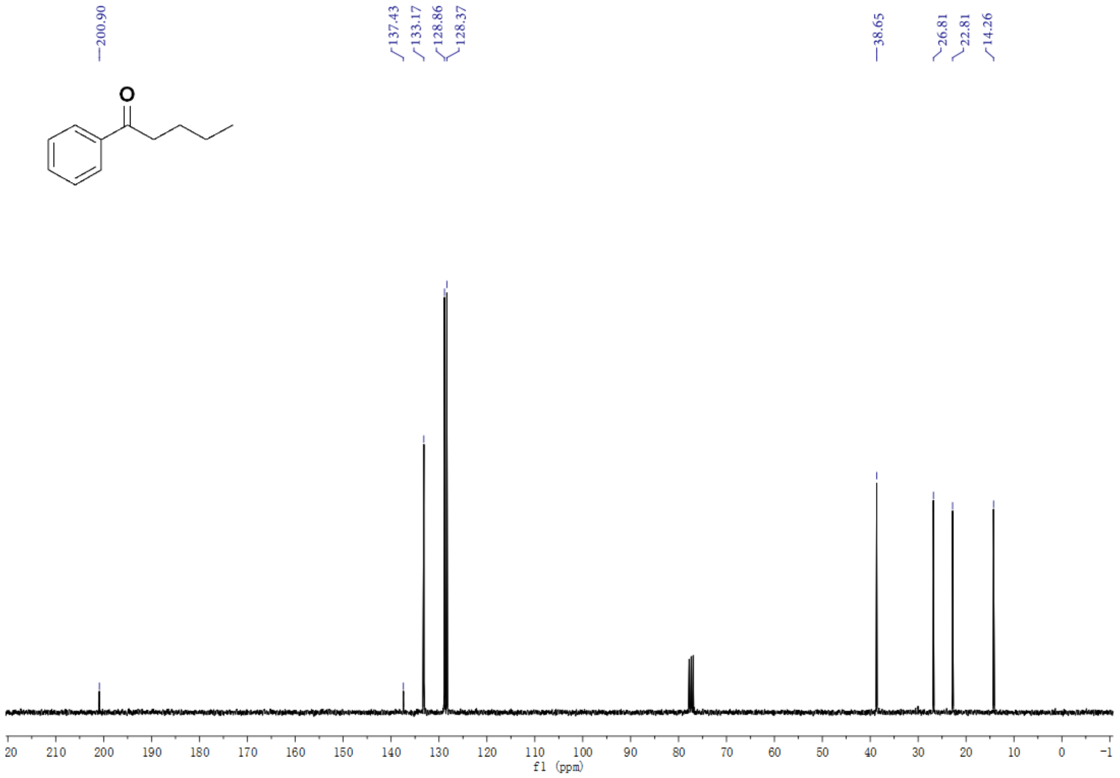

2ab

^{1}H NMR (300 MHz, $CDCl_3$)

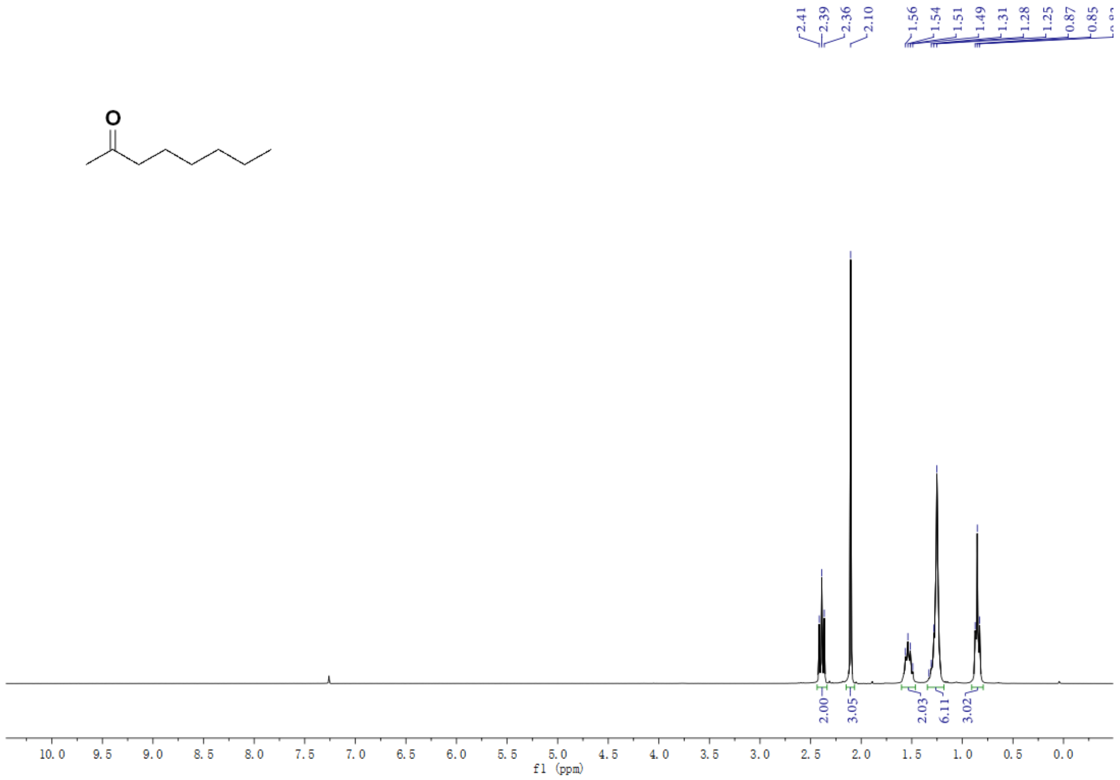

^{13}C NMR (75 MHz, $CDCl_3$)

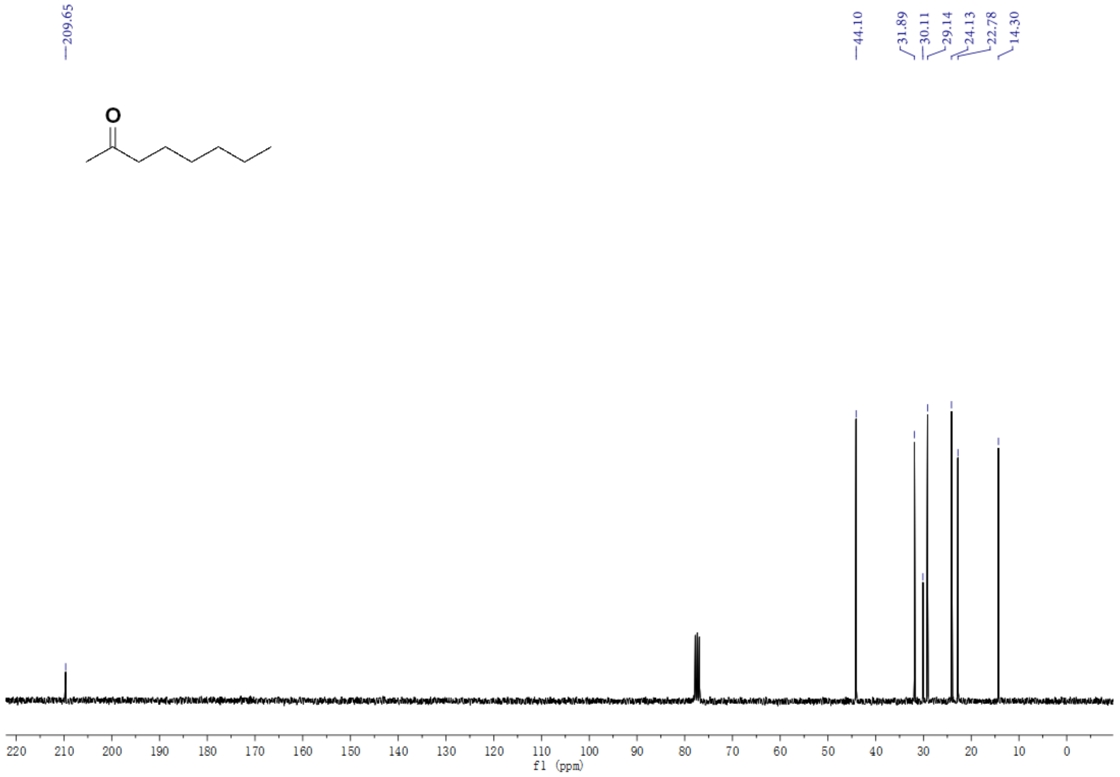

2ad

^{1}H NMR (300 MHz, $CDCl_3$)

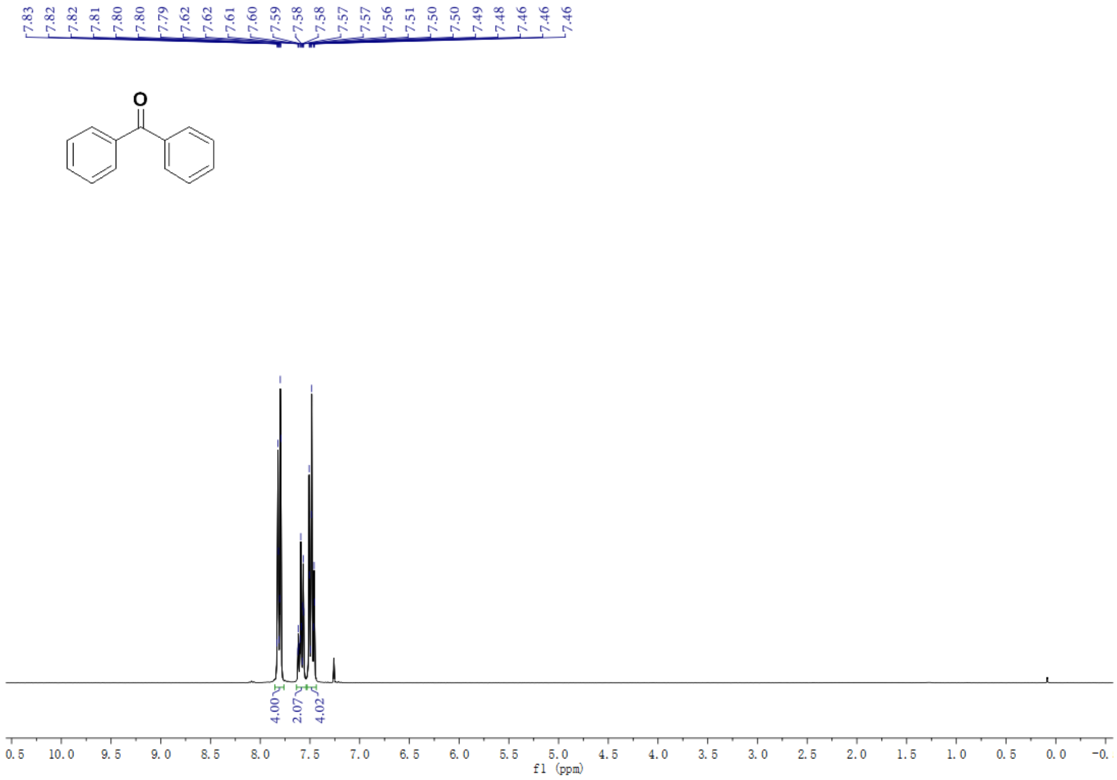

^{13}C NMR (75 MHz, $CDCl_3$)

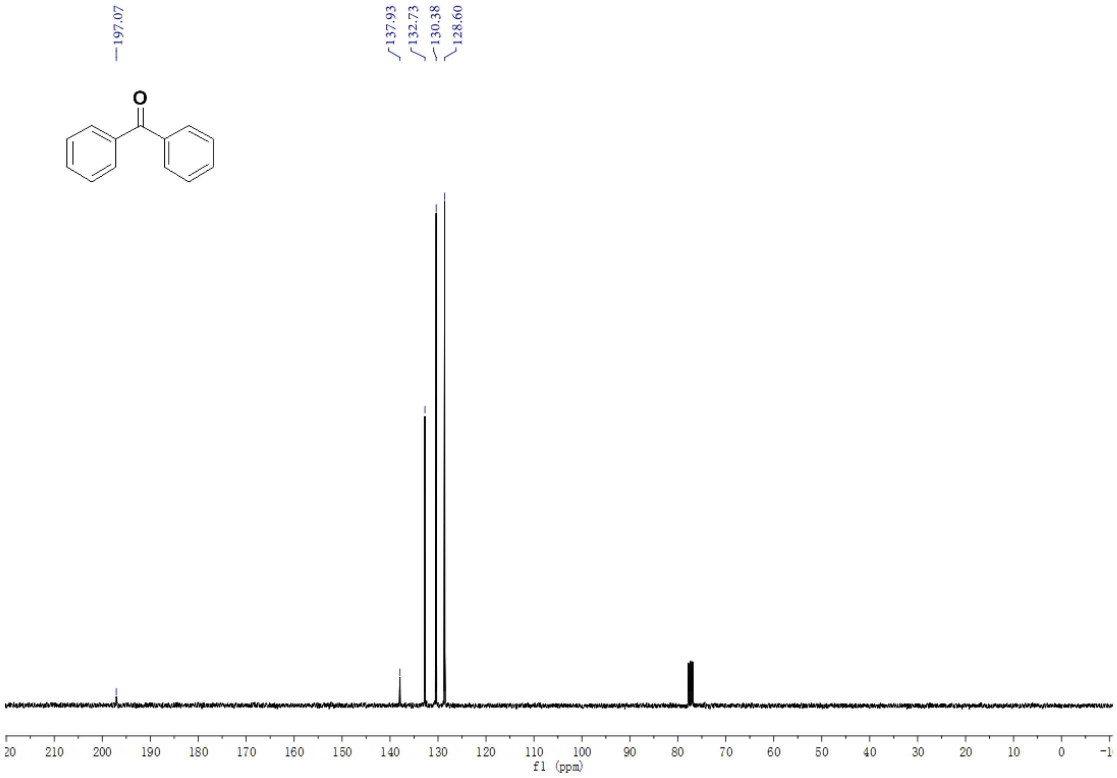

2ae

^{1}H NMR (300 MHz, DMSO)

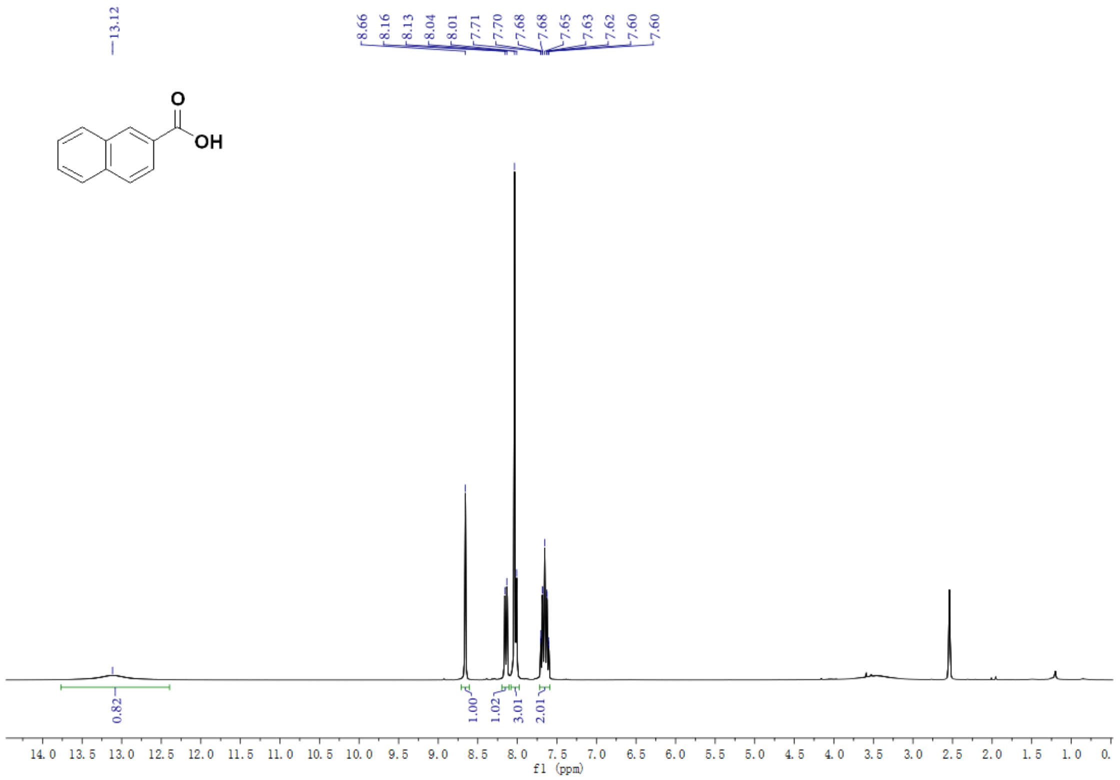

^{13}C NMR (75 MHz, DMSO)

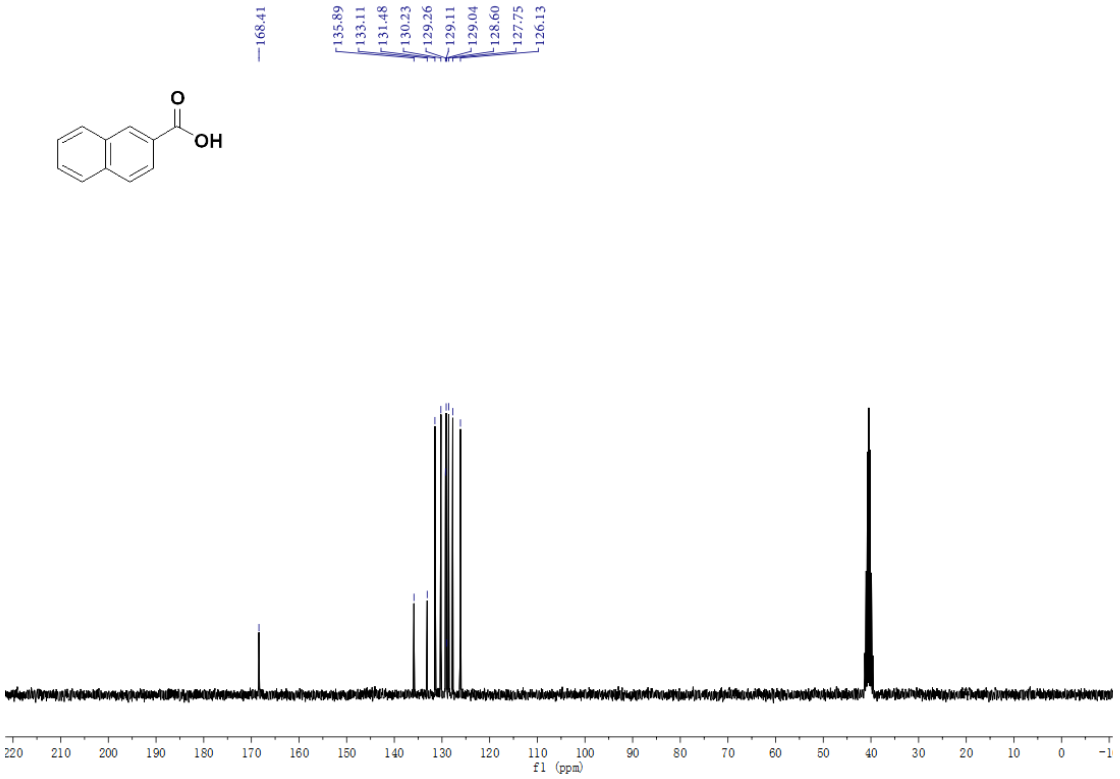

2af

^{1}**H NMR** (500 MHz, DMSO)

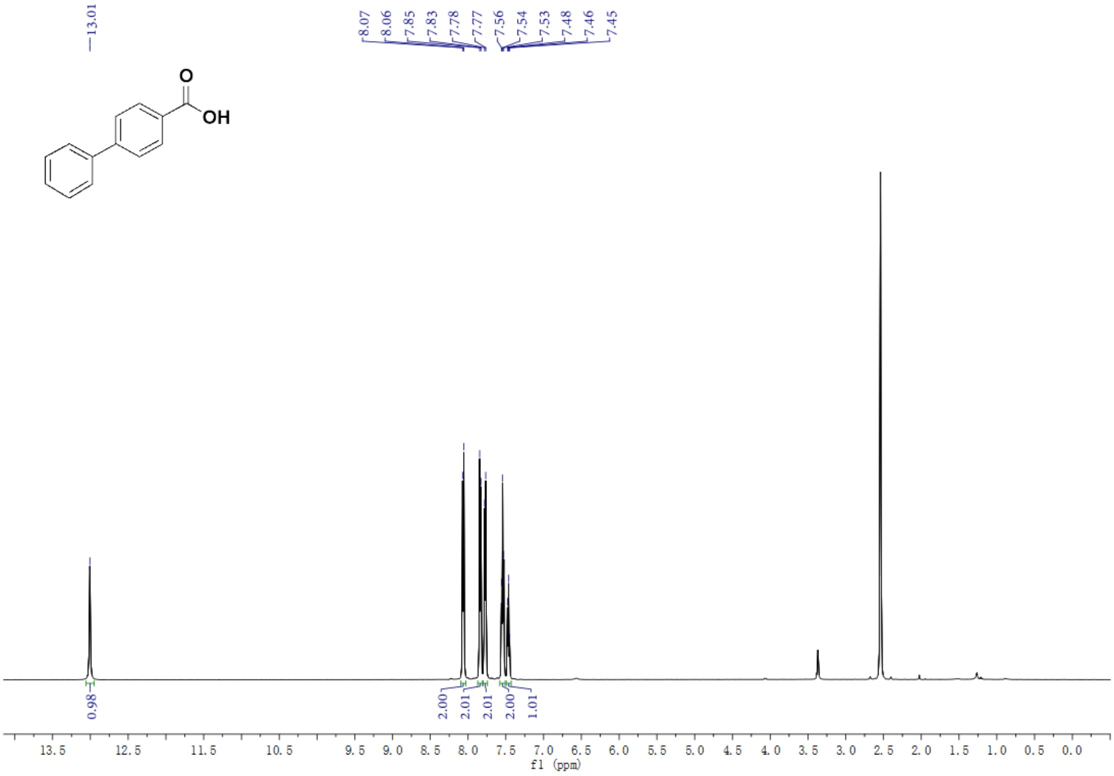

^{13}**C NMR** (126 MHz, DMSO)

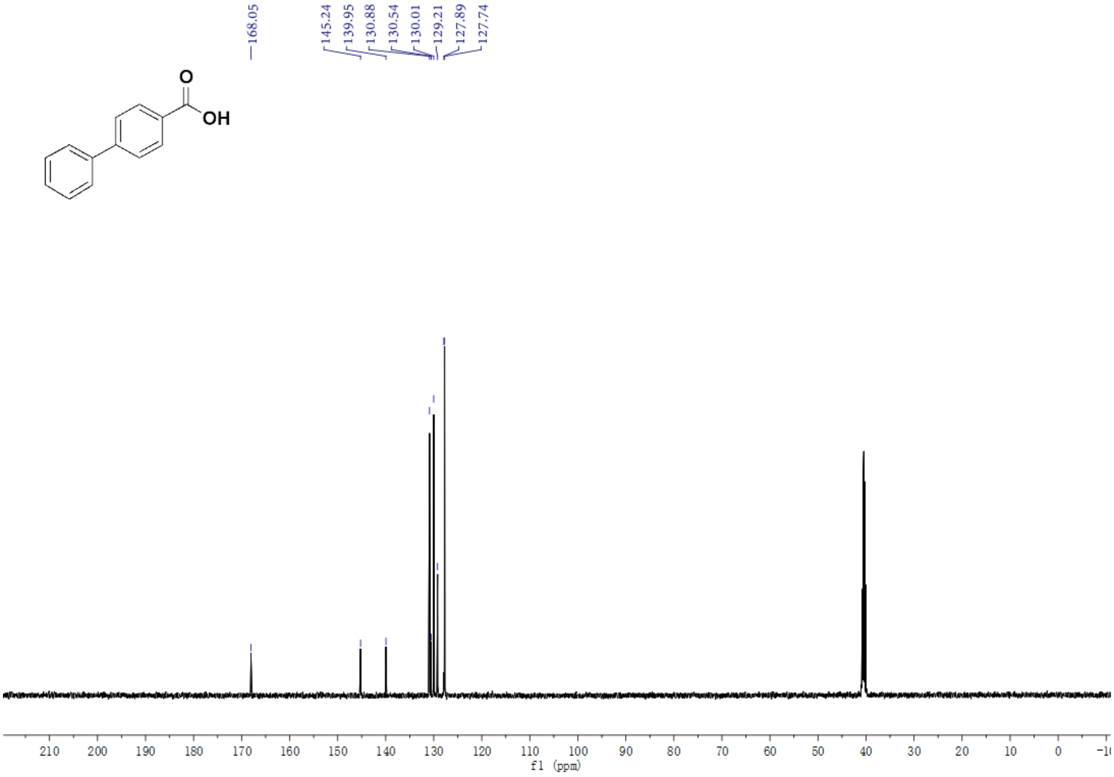

2ag

^{1}H NMR (300 MHz, $CDCl_3$)

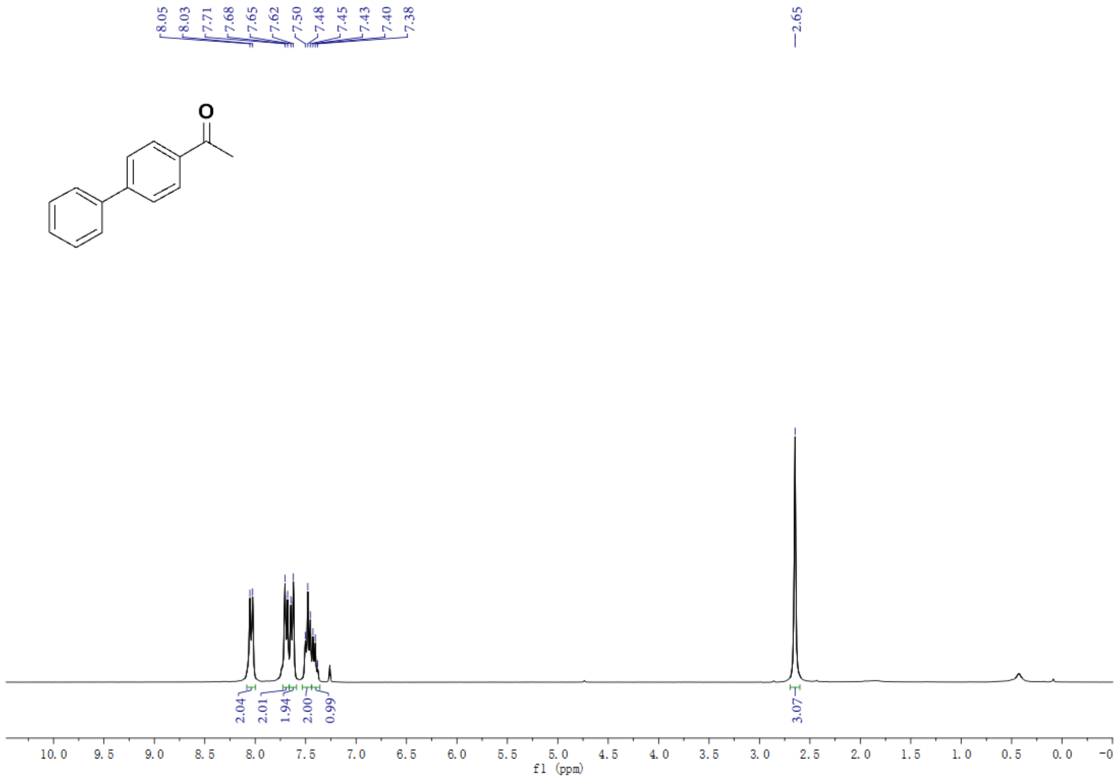

^{13}C NMR (75 MHz, $CDCl_3$)

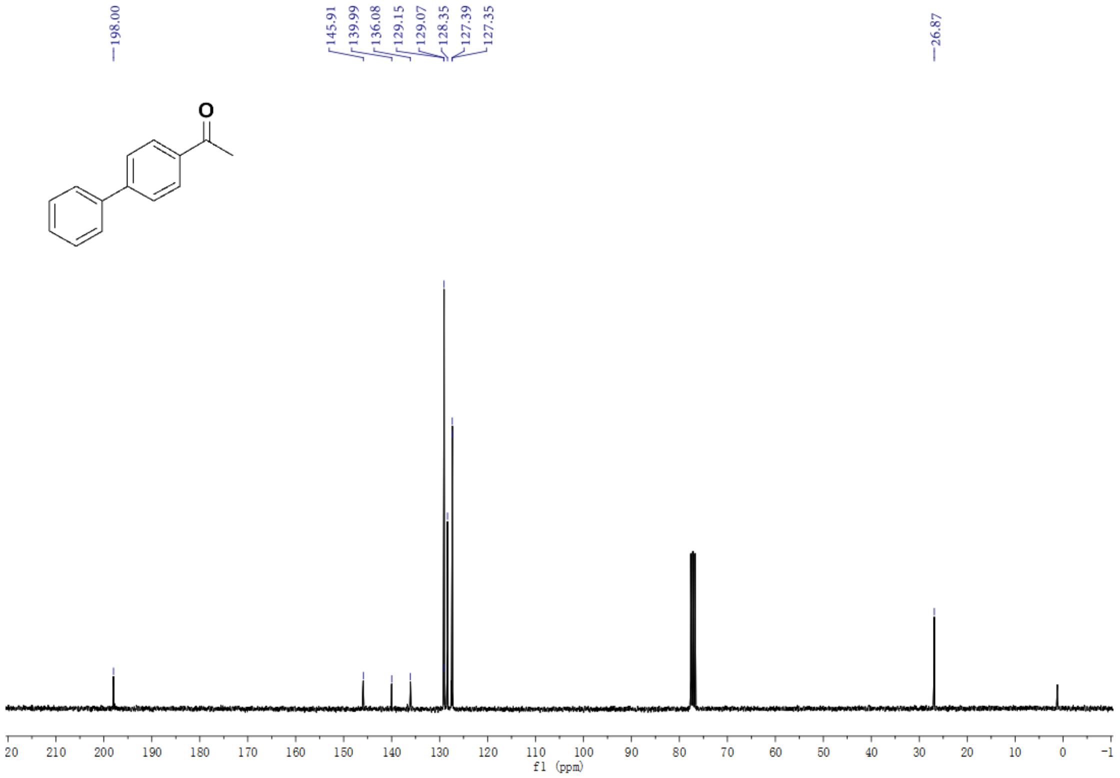

1ah

^{1}H NMR (300 MHz, $CDCl_3$)

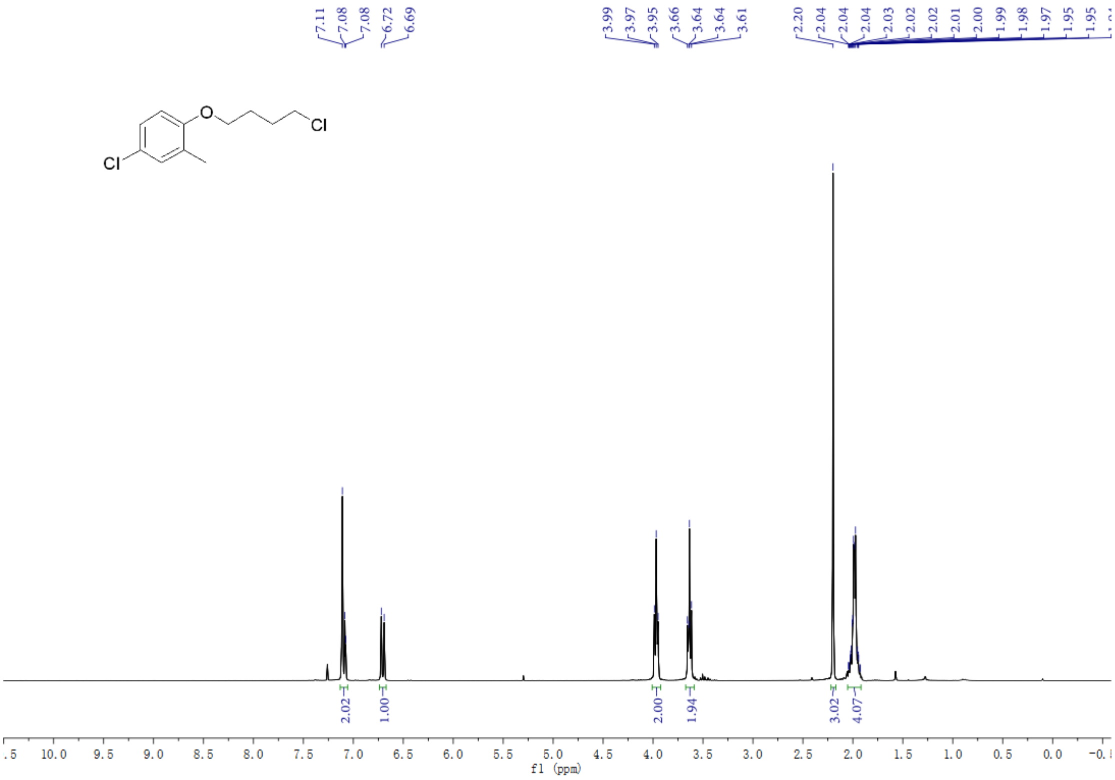

^{13}C NMR (75 MHz, $CDCl_3$)

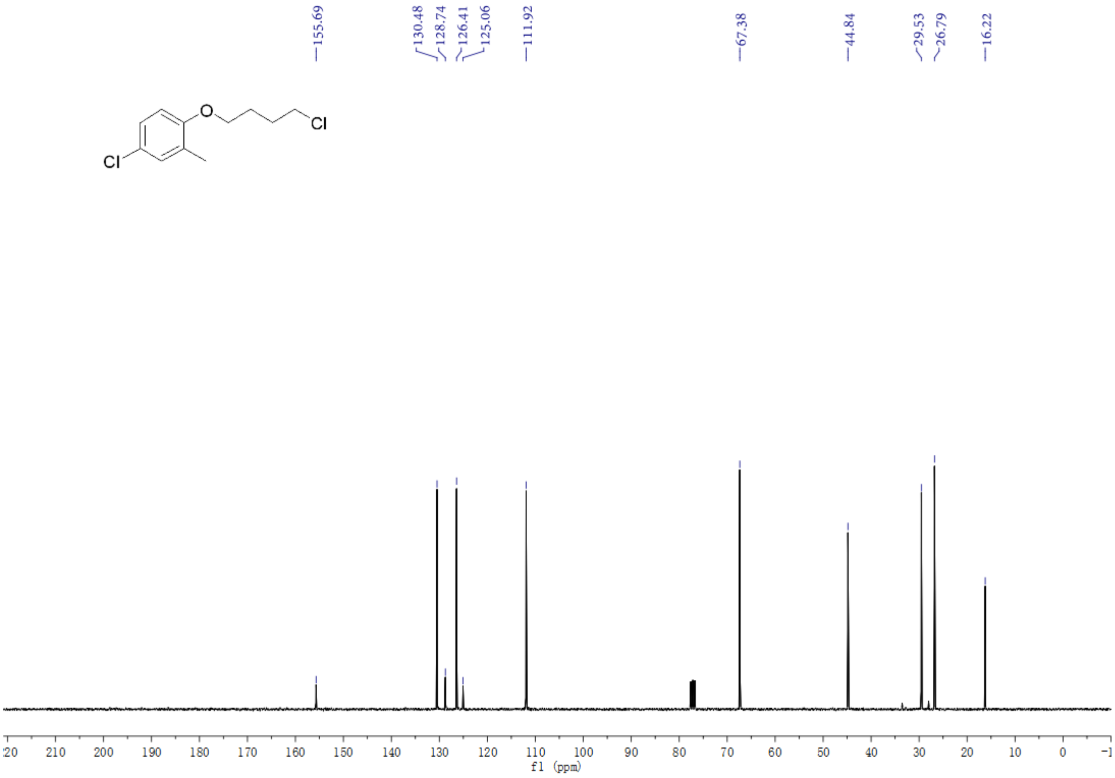

2ah

^{1}H NMR (300 MHz, $CDCl_3$)

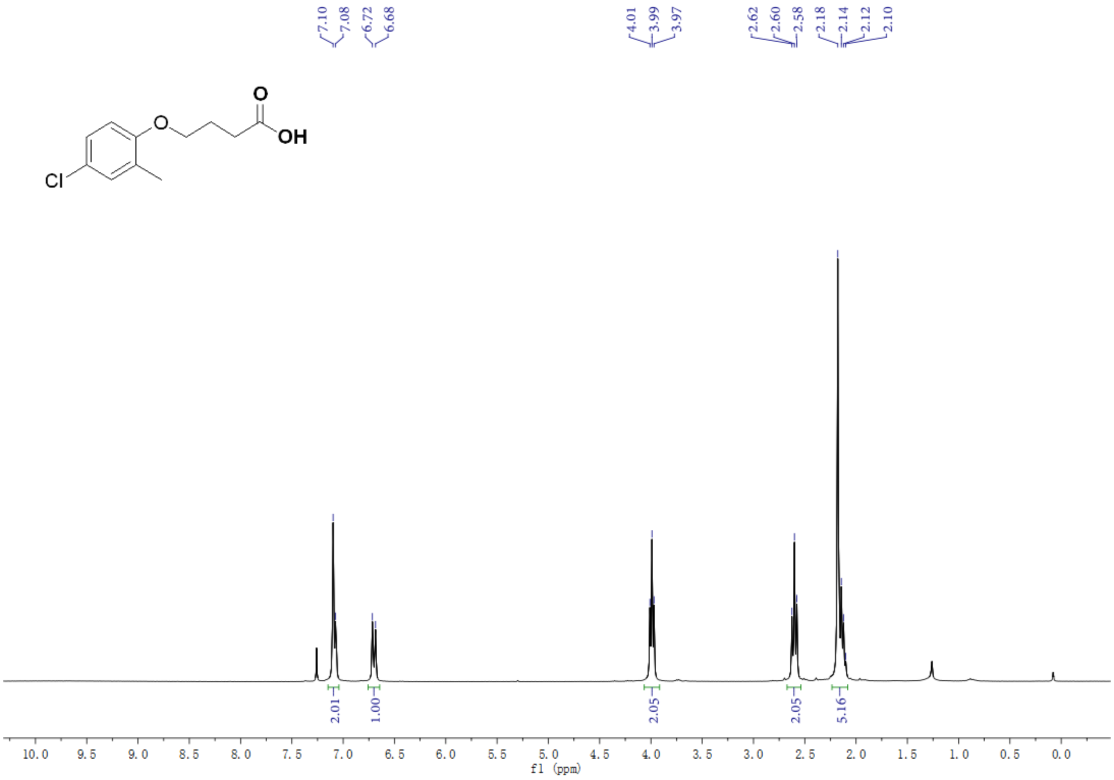

^{13}C NMR (75 MHz, $CDCl_3$)

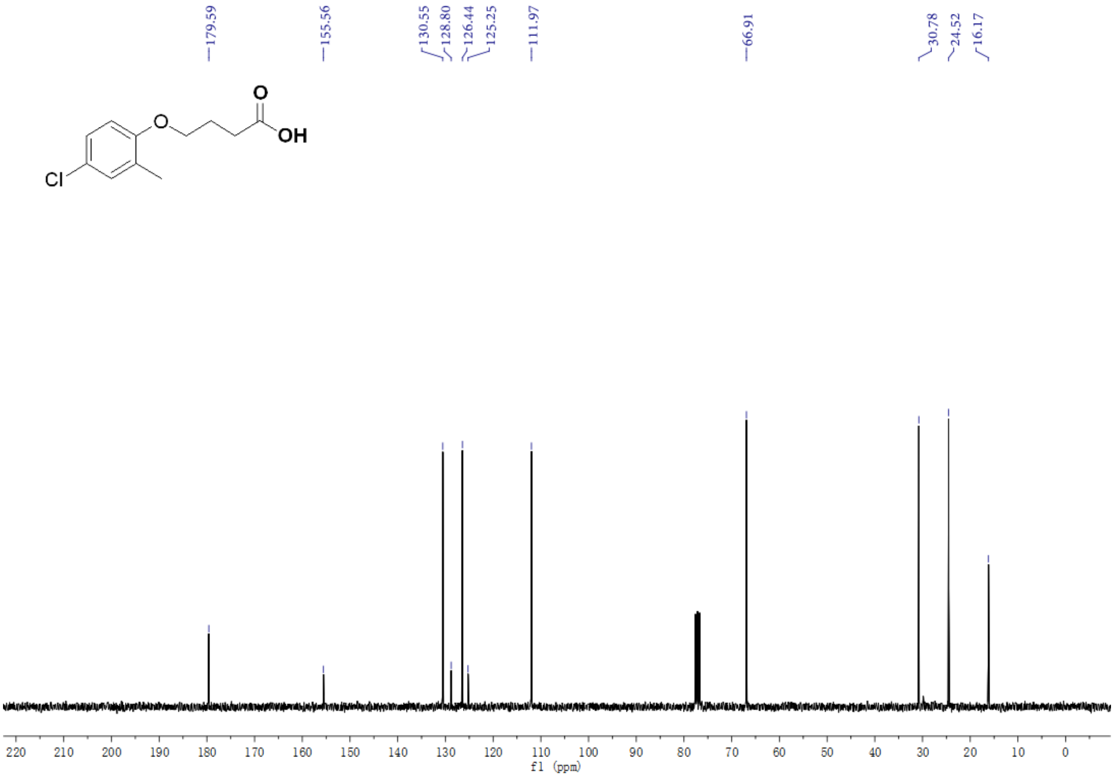

2ai

^{1}H NMR (400 MHz, $CDCl_3$)

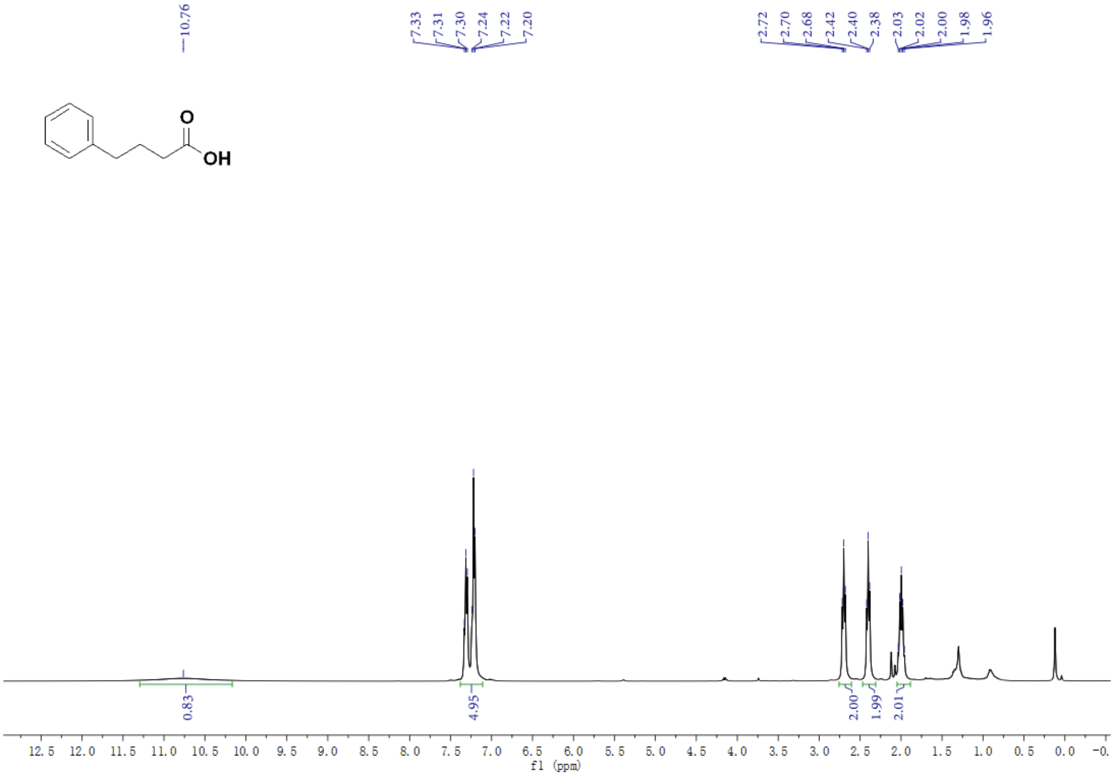

^{13}C NMR (101 MHz, $CDCl_3$)

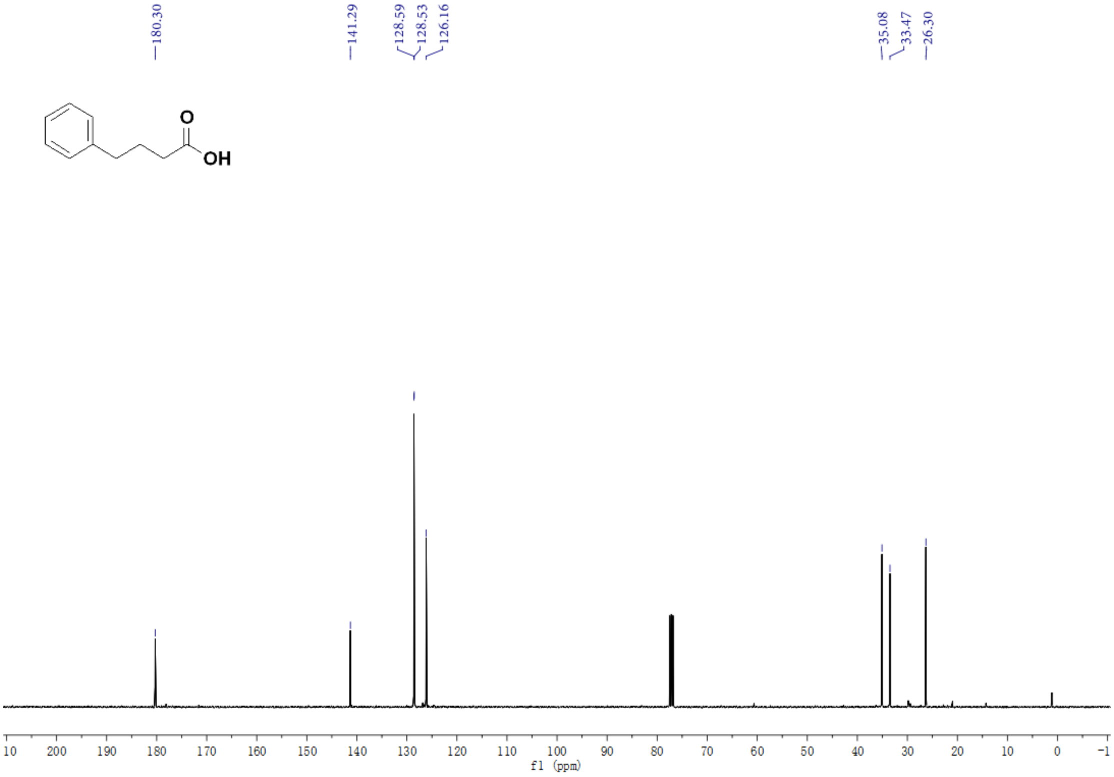

7. References

1. Zhang, J.; Leitus, G.; Ben-David, Y.; Milstein. D. Facile Conversion of Alcohols into Esters and Dihydrogen Catalyzed by New Ruthenium Complexes. *J. Am. Chem. Soc.* **2005**, *127*, 10840-10841.
2. Balaraman, E.; Gnanaprakasam, B.; Shimon, L. J. W.; Milstein, D. Direct Hydrogenation of Amides to Alcohols and Amines under Mild Conditions. *J. Am. Chem. Soc.* **2010**, *132*, 16756-16758.
3. Gunanathan, C.; Milstein, D. Selective Synthesis of Primary Amines Directly from Alcohols and Ammonia. *Angew. Chem. Int. Ed.* **2008**, *47*, 8661-8664.
4. Tang, S.; Rauch, M.; Montag, M.; Diskin-Posner, Y.; Ben-David, Y.; Milstein. D. Catalytic Oxidative Deamination by Water with H_2 Liberation. *J. Am. Chem. Soc.* **2020**, *142*, 20875-20882.
5. Leicht, H.; Göttker-Schnetmann, I.; Mecking, S. Synergetic Effect of Monomer Functional Group Coordination in Catalytic Insertion Polymerization. *J. Am. Chem. Soc.* **2017**, *139*, 6823-6826.
6. Venning, A. R. O.; Bohan, P. T.; Alexanian, E. J. Palladium-Catalyzed, Ring-Forming Aromatic C-H Alkylations with Unactivated Alkyl Halides. *J. Am. Chem. Soc.* **2015**, *137*, 3731-3734.
7. Tyrol, C. C.; Yone, N. S.; Gallin. C. F.; Byers, J. A. Iron-catalysed enantioconvergent Suzuki-Miyaura cross-coupling to afford enantioenriched 1,1-diarylalkanes. *Chem. Commun.* **2020**, *56*, 14661-14664.
8. Zhou, J.; Zhao, Z.; Mori, S.; Yamamoto, K.; Shibata, N. Cross-coupling of organic fluorides with allenes: a silyl-radical-relay pathway for the construction of α-alkynyl-substituted all-carbon quaternary centres. *Chem. Sci.* **2024**, *15*, 5113-5122.
9. He, T.; Klare, H. F. T.; Oestreich, M. Silylium-Ion Regeneration by Protodesilylation Enables Friedel-Crafts Alkylation with Less Isomerization and No Defunctionalization. *ACS Catal.* **2021**, *11*, 12186-12193.
10. Wang, S.-J.; Zhai, J.-J.; Wang, L.; Tang, X.-Y. Consecutive regulation of catalytic activities

of $B(C_6F_5)_3 \cdot H_2O$: direct nucleophilic substitution of benzyl fluorides with alcohol via dual activation, *Org. Chem. Front.* **2023**, *10*, 3849-3855.

11. Li, X.-L.; Zhang, K.; Jiang, J.-L.; Zhu, R.; Wu, W.-P.; Deng, J.; Fu, Y. Synthesis of medium-chain carboxylic acids or α,ω-dicarboxylic acids from cellulose-derived platform chemicals. *Green Chem.* **2018**, 20, 362-368.
12. Jamieson A. G.; Sutherland, A. Ether-directed palladium(II)-catalysed aza-Claisen rearrangements: studies on the origin of the directing effect. *Tetrahedron* **2007**, *63*, 2123-2131.
13. Meng, Q.-Y.; Wang, S.; Huff, G.; König, B. Ligand-Controlled Regioselective Hydrocarboxylation of Styrenes with CO_2 by Combining Visible Light and Nickel Catalysis. *J. Am. Chem. Soc.* **2018**, *140*, 3198-3201.
14. Adusumalli, S. R.; Rawale, D. G.; Singh, U.; Tripathi, P.; Paul, R.; Kalra, N.; Mishra, R. K.; Shukla, S.; Rai, V. Single-Site Labeling of Native Proteins Enabled by a Chemoselective and Site-Selective Chemical Technology. *J. Am. Chem. Soc.* **2018**, *140*, 15114-15123.
15. Nishimoto, Y.; Okita, A.; Yasuda, M.; Baba, A. Synthesis of a Wide Range of Thioethers by Indium Triiodide Catalyzed Direct Coupling between Alkyl Acetates and Thiosilanes. *Org. Lett.* **2012**, *14*, 1846-1849.
16. Benson, S. C.; Li, J.-H.; Snyder, J. K. Indole as a Dienophile in Inverse Electron Demand Diels-Alder Reactions. 3. Intramolecular Reactions with 1,2,4-Triazines To Access the Canthine Skeleton. *J. Org. Chem.* **1992**, *57*, 5285-5287.
17. Meng, Q.-Y.; Wang, S.; König, B. Carboxylation of Aromatic and Aliphatic Bromides and Triflates with CO_2 by Dual Visible-Light–Nickel Catalysis. *Angew. Chem. Int. Ed.* **2017**, *56*, 13426-13430.
18. Mao, Y.; Liu, Y.; Hu, Y.; Wang, L.; Zhang, S.; Wang, W. Pd-Catalyzed Debenzylation and Deallylation of Ethers and Esters with Sodium Hydride. *ACS Catal.* **2018**, *8*, 3016-3020.
19. Correa, K.; León, T.; Martin, R. Ni-Catalyzed Carboxylation of $C(sp^2)$- and $C(sp^3)$-O Bonds with CO_2. *J. Am. Chem. Soc.* **2014**, *136*, 1062-1069.
20. Deng, Y.; Wei, X.-J.; Wang, H.; Sun, Y.; Noël, T.; Wang, X. Disulfide-Catalyzed Visible-Light-Mediated Oxidative Cleavage of C=C Bonds and Evidence of an Olefin-Disulfide Charge-Transfer Complex. *Angew. Chem. Int. Ed.* **2017**, *56*, 832-836.

21. Wu, J.; Yang, X.; He, Z.; Mao, X.; T. Hatton, T. A.; Jamison, T. F. Continuous Flow Synthesis of Ketones from Carbon Dioxide and Organolithium or Grignard Reagents. *Angew. Chem. Int. Ed.* **2014**, *53*, 8416-8420.

22. Wang, Y.-F.; Gao, Y.-R.; Mao, S.; Zhang, Y.-L.; Guo, D.-D.; Yan, Z.-L.; Guo, S.-H.; Wang, Y.-Q. Wacker-Type Oxidation and Dehydrogenation of Terminal Olefins Using Molecular Oxygen as the Sole Oxidant without Adding Ligand. *Org. Lett.* **2014**, *16*, 1610-1613.

23. Tang, S.; Ben-David, Y.; Milstein, D. Oxidation of Alkenes by Water with H_2 Liberation. *J. Am. Chem. Soc.* **2020**, *142*, 5980-5984.

24. Jiang, K.; Wang, H.; Xie, Y.; Jiang, H.; Lei, M.; Yin, B. Remote-Group-Assisted Facile Oxidative Arylation of Furans and Pyrroles. *ACS Catal.* **2023**, *13*, 3520-3531.

25. Sharma, K. S.; Tiwari, S. K.; Pandey, G. Intramolecular Cyclization of -COY (Y = OH, NHPh) to Photoredox Functionalized Benzylic C(sp3)-H Bond. Attractive Approach to Construct γ, δ-lactones and lactams Scaffolds. *Asian J. Org. Chem.* **2024**, *13*, e202400114.

26. Gracia, S.; Cazorla, C.; Métay, E.; Pellet-Rostaing, S.; Lemaire, M. Synthesis of 3-Aryl-8-oxo-5,6,7,8-tetrahydroindolizines via a Palladium-Catalyzed Arylation and Heteroarylation. *J. Org. Chem.* **2009**, *74*, 3160-3163.

27. Lellouche, J.-P.; Pomerantz, Z.; Ghosh, S. Towards hybrid carbazole/pyrrole-based carboxylated monomers: chemical synthesis, characterisation and electro-oxidation properties. *Tetrahedron Lett.* **2011**, *52*, 6903-6907.

28. Takeuchi, R.; Ishii, N.; Sugiura, M.; Sato, N. The Highly Regioselective Carbonylation of Vinylsilanes. *J. Org. Chem.* **1992**, *57*, 4189-4194.

29. Li, L.; Yan, Z.-X.; Ran, C.-K.; Liu, Y.; Zhang, S.; Gao, T.-Y.; Dai, L.-F.; Liao, L.-L.; Ye, J.-H.; Yu, D.-G. Electro-reductive carboxylation of C-Cl bonds in unactivated alkyl chlorides and polyvinyl chloride with CO_2. *Chinese Chem. Lett.* **2024**, *35*, 110104.

30. Galli, M.; Fletcher, C. J.; del Pozob, M.; Goldup, S. M. Scalable anti-Markovnikov hydrobromination of aliphatic and aromatic olefins. *Org. Biomol. Chem.* **2016**, *14*, 5622-5626.

31. Li, Z.-Q.; Fu, Y.; Deng, R.; Tran, V. T.; Gao, Y.; Liu, P.; Engle, K. M. Ligand-Controlled Regiodivergence in Nickel-Catalyzed Hydroarylation and Hydroalkenylation of Alkenyl Carboxylic Acids. *Angew. Chem. Int. Ed.* **2020**, *59*, 23306-23312.
